# New phenolic bisabolane sesquiterpenoids discovered from the marine-derived fungus *Aspergillus sydowii* assisted by molecular networking and SMART strategies

**DOI:** 10.1080/21501203.2025.2547630

**Published:** 2025-09-14

**Authors:** Baodan Zhang, Meiyan Bao, Ling Liu

**Affiliations:** a State Key Laboratory of Microbial Diversity and Innovative Utilization, Institute of Microbiology, Chinese Academy of Sciences, Beijing, China; b College of Life Sciences, University of Chinese Academy of Sciences, Beijing, China

**Keywords:** Molecular networking, SMART, phenolic bisabolane sesquiterpenoids, marine associated fungus, antifungal assays, *Cryptococcus* spp.

## Abstract

Three previously undescribed phenolic bisabolane sesquiterpenoids (PBS) including a pair of new enantiomers (±)-aspersydonol A (**1a**/**1b**) and aspersydonol B (**2**), along with 12 known analogs (**3**–**14**) were isolated from the marine-associated fungus *Aspergillus sydowii* LF51 guided by a combined application of molecular networking and Small Molecule Accurate Recognition Technology (SMART) approaches. Their structures were elucidated by spectroscopic analyses and electronic circular dichroism (ECD) calculations. Compound **2** represents the first example of natural PBS with a hexahydrodibenzo[*b,d*]furan skeleton. Antifungal assays revealed that compounds **3**, **6**, **9**, and **11** exhibited moderate activities against *Cryptococcus* spp. (MICs = 32–64 μg/mL), with additive effects against *Cryptococcus gattii* R265 combined with Amphotericin B (AmB), reducing MICs to 4–32 μg/mL. Compound **9** showed an additive effect with fluconazole (FLC) against *C. gattii* R265, lowering its MIC to 2 μg/mL. Mechanistic studies revealed that compound **9** inhibited *C. gattii* R265 by suppressing urease activity, disrupting membrane integrity, and inducing oxidative damage *via* ROS accumulation. Furthermore, compound **3** displayed moderate antibacterial efficacy against *Mycobacterium smegmatis*.

## GRAPHICAL ABSTRACT

**Figure:**
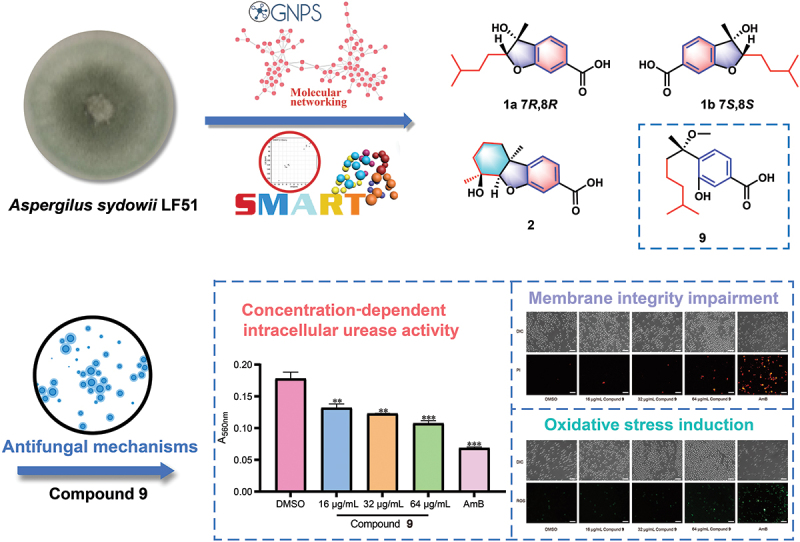

## 1. Introduction

Cryptococcosis, a globally distributed invasive fungal infection primarily caused by *Cryptococcus neoformans* and *C. gattii*, poses a significant global health burden (Ferreira et al. 2015; Perfect and Bicanic 2015; Ibe et al. 2023). Current estimates indicate approximately one million annual cases worldwide, resulting in roughly 625,000 deaths (Bagheri-Josheghani et al. 2025). Standard therapeutic regimens primarily rely on AmB, 5-fluorocytosine (5-FC), and FLC, administered as monotherapy or in combination (Donlin et al. 2021; Sousa et al. 2023; Qureshi et al. 2024). However, treatment efficacy remains suboptimal due to high cost, toxicity, and drug resistance (Carvajal et al. 2023; Leocádio et al. 2025). While newer antifungals such as ibrexafungerp, oteseconazole, and isavuconazole have recently gained clinical approval, they lack indications for cryptococcosis (Freitas et al. 2023). The high incidence and mortality of this disease, combined with the inadequacy of current therapies, underscore the critical need for novel antifungal drug development (Alves et al. 2023; Luo et al. 2024).

Bisabolane-type sesquiterpenoids are a class of monocyclic natural compounds defined by a six-membered carbon-based cyclic structure and an eight-carbon side chain (Li et al. 2023). When the six-membered ring in these molecules adopts a benzene ring structure, this specific group of compounds is formally termed phenolic bisabolane-type sesquiterpenoids (PBS) due to its unique phenolic aromatic system (Chen et al. 2021; Salman et al. 2023). The structural diversity of PBS emerges from chemical modifications to its side chain—specifically oxidation (electron loss), reduction (electron gain), esterification (ester bond formation), and cyclisation (ring closure) (Li et al. 2022). These reactions generate functional groups like hydroxyls and double bonds, as well as heterocyclic frameworks such as furan, pyran, and oxepane rings, ultimately boosting molecular complexity (Niu et al. 2020). PBS derivatives demonstrate broad-spectrum bioactivities encompassing cytotoxic (Sun et al. 2012; Jiang et al. 2020), anti-inflammatory (Chung et al. 2013), antimicrobial effects (Li et al. 2015; Li et al. 2019), and antiviral activities (Du et al. 2025). In contrast, exploration of their antifungal efficacy is notably limited (Shu et al. 2021; Li et al. 2022). Therefore, it is essential to evaluate the antifungal capabilities of these derivatives.

Marine-derived fungi thrive in extreme environments characterised by high salinity, low pressure, and oxygen scarcity, resulting in the production of structurally unique bioactive compounds (Ren et al. 2021; Virués-Segovia et al. 2023; Zheng et al. 2025). However, traditional random isolation methods often fail to efficiently uncover these molecules (Louwen et al. 2023). To address this limitation, advanced tools like SMART (which clusters related compounds for rapid family identification) and molecular networking (a computational strategy enabling targeted analysis of natural products in complex mixtures) offer transformative solutions (Zhang et al. 2017; Wang et al. 2025). By combining SMART’s clustering capabilities with molecular networking’s mixture deconvolution power, researchers can prioritise novel compound families from marine fungi, accelerating their directed discovery and isolation.

**Figure 1.**
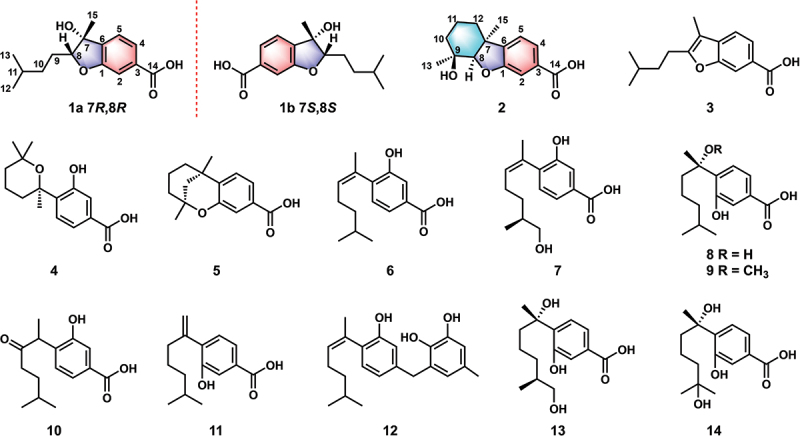
The chemical structures for compounds **1**–**14**.

During our exploration of new secondary metabolites from fungi, the strain *Aspergillus sydowii* LF51, isolated from South Atlantic sediments, was screened for chemical investigation. Guided by an integrated approach combining SMART and molecular networking, the crude extract of this fungus was fractionated to afford 15 PBS, including a pair of new enantiomers (±)-aspersydonol A (**1a**/**1b**) and aspersydonol B (**2**), as well as 12 known analogues (**3**–**14**) (Figure 1). Herein, we report the purification, structural elucidation, and bioactivity assessment of the isolated compounds, accompanied by preliminary antifungal mechanism studies.

## 2. Materials and methods

### 2.1. General experimental procedures

In this study, all instruments and reagents are offered in Table S1.

### 2.2. Strain and fermentation

Isolated from South Atlantic deep-sea sediments in November 2012, the fungal strain *A. sydowii* LF51 is preserved at the Institute of Microbiology, Chinese Academy of Sciences (IMCAS). The strain was identified through phylogenetic analysis of ITS (NMDCN00097QG), CaM (NMDCN00098PI), BenA (NMDCN00098PH), and RPB2 (NMDCN00098PG) sequences combined with morphological observation.

To obtain a broader diversity of compounds, following the OSMAC strategy, the fungus was fermented in 10 distinct media (Table S2). Each medium was tested with and without sea salt and different incubation times (Table S3) as controls to compare their metabolic profiles. HPLC-UV profiles showed that the rice medium extract (no sea salt, long-term fermentation) had distinct differences (Figures S1–S4), so large-scale fermentation was based on this condition. Scaling-up utilised solid-state fermentation in 500 mL flasks containing 100 g rice and 110 mL of water, inoculated with 10 mL seed culture, then maintained statically at 28 °C for 40 d.

### 2.3. Extraction and isolation

The fermented rice substrate underwent triple extraction with ethyl acetate (EtOAc), yielding 65.0 g of crude extract. This extract was subjected to silica gel column chromatography (CC) using a petroleum ether (PE)/EtOAc gradient (15:1 to 1:2, v/v), generating seven major fractions (Fr.1–Fr.7). Fr.4 was further fractionated through silica gel CC with sequential solvent systems of PE/dichloromethane (DCM) (10:1 to 0:1) followed by DCM/MeOH (500:1 to 30:1), producing eight subfractions (Fr.4.1–Fr.4.8). Purification of Fr.4.3 *via* octadecylsilyl (ODS) CC with a MeOH/H₂O gradient (3:7 to 1:0, v/v) afforded five refined subfractions (Fr.4.3.1–Fr.4.3.5). Subsequent reversed phase high performance liquid chromatography (RP-HPLC; Kromasil 5-CelluCoat column, 250 × 4.6 mm, 5 μm) analysis for Fr.4.3.2 (MeOH/H₂O, 79:21, 2.0 mL/min) afforded compounds **3** (2.9 mg, *t*_R_ 36.0 min), **4** (4.0 mg, *t*_R_ 14.8 min), **5** (3.5 mg, *t*_R_ 18.0 min), and **9** (15.0 mg, *t*_R_ 24.8 min). Similarly, Fr.4.3.3 (MeOH/H₂O 76:24, 2.0 mL/min) yielded compounds **10** (3.5 mg, *t*_R_ 31.5 min) and **6** (3.0 mg, *t*_R_ 26.2 min), while Fr.4.3.4 (MeOH/H₂O 65:35, 2.0 mL/min) produced compounds **1** (3.0 mg, *t*_R_ 38.0 min) and **2** (2.0 mg, *t*_R_ 12.5 min). Fr.4.4 delivered compounds **11** (4.0 mg, *t*_R_ 57.0 min), **13** (5.6 mg, *t*_R_ 23.0 min), **14** (11.0 mg, *t*_R_ 26.0 min), and **8** (50.0 mg, *t*_R_ 29.0 min) under RP-HPLC conditions (MeOH/H₂O, 68:32, 2.0 mL/min).

Fr.5 was separated using silica gel CC with a petroleum PE/EtOAc gradient (15:1 to 1:2, v/v), resulting in eight subfractions (Fr.5.1–5.8). Fr.5.3 was further purified by RP-HPLC with a MeOH/H₂O (70:30) mobile phase, yielding compound **12** (2.0 mg, *t*_R_ 25.7 min). Fr.7 was separated by silica gel CC using a petroleum PE/EtOAc gradient (15:1 to 1:2, v/v), yielding six subfractions (Fr.7.1–7.6). Further purification of Fr.7.5 *via* RP-HPLC (MeOH/H₂O 55:45, 2.0 mL/min) afforded compound **7** (3.5 mg, *t*_R_ 47.5 min).

(+)-Aspersydonol A (**1a**): pale yellow oil; ${\left[\alpha \right]}_{D}^{25}$ = +70.0 (*c* 0.1, CH_3_OH); UV (CH_3_OH) *λ*_max_ (log *ε*) 225 (3.26), 274 (0.82), 301 (1.90) nm; IR (neat) *ν*_max_ 3,368, 2,955, 2,870, 1,693, 1,592, 1,435, 1,288 cm^−1^; ECD (7.58 × 10^−4^ M, CH_3_OH) *λ*_max_ (Δ*ε*) 216 (+22.45), 244 (−6.83), 269 (+0.21) nm; positive HRESIMS at *m*/*z* 247.1333 [M – H_2_O + H]^+^ (calcd for C_15_H_19_O_3_ *m*/*z* 247.1329), corresponding to the molecular formula C_15_H_20_O_4_.

(–)-Aspersydonol A (**1b**): pale yellow oil; ${\left[\alpha \right]}_{D}^{25}$ = −65.0 (*c* 0.1, CH_3_OH); UV (CH_3_OH) *λ*_max_ (log *ε*) 225 (3.26), 274 (0.82), 301 (1.90) nm; IR (neat) *ν*_max_ 3,368, 2,955, 2,870, 1,693, 1,592, 1,435, 1,288 cm^−1^; ECD (5.68 × 10^−4^ M, CH_3_OH) *λ*_max_ (Δ*ε*) 216 (−16.84), 244 (+5.12), 269 (−0.16) nm; positive HRESIMS at *m*/*z* 247.1333 [M – H_2_O + H]^+^ (calcd for C_15_H_19_O_3_ *m*/*z* 247.1329), corresponding to the molecular formula C_15_H_20_O_4_.

Aspersydonol B (**2**): pale yellow oil; ${\left[\alpha \right]}_{D}^{25}$ = +24.0 (*c* 0.1, CH_3_OH); UV (CH_3_OH) *λ*_max_ (log *ε*) 237 (3.27), 271 (1.25), 298 (3.17) nm; IR (neat) *ν*_max_ 2,935, 1,689, 1,589, 1,434, 1,259, 988, 767 cm^−1^; ECD (1.14 × 10^−3^ M, CH_3_OH) *λ*_max_ (Δ*ε*) 209 (+6.54), 244 (−1.54), 300 (+0.74) nm; positive HRESIMS at *m*/*z* 263.1276 [M + H]^+^ (calcd for C_15_H_19_O_4_ *m*/*z* 263.1278).

### 2.4. ECD calculation

ECD calculation method was referred to the previous studies (Zhang et al. 2022; Lu et al. 2024).

### 2.5. Antimicrobial assays

Antimicrobial assay protocols are detailed in the supplemental material.

### 2.6. Drug combination testing

The checkerboard assay was performed to assess synergistic interactions between test compounds and antifungal agents (AmB and FLC). Horizontally, 100 μL of compound solutions at varying concentrations were added, while vertically, 100 μL of AmB or FLC solutions at different concentrations were dispensed. Equivalent aliquots of *C. gattii* R265 fungal suspensions were introduced into individual culture wells. Post 72 h aerobic incubation at a physiologic temperature (37 °C), fungal proliferation within the microtiter plate was quantitatively evaluated. The pharmacological interaction profile was quantitatively characterised through the computation of the Fractional Inhibitory Concentration Index (FICI), employing the standardised formula (Wang et al. 2024):

$$\mathrm{FICI}=\frac{\mathrm{MI}C_{A}(\mathrm{combined})}{\mathrm{MI}C_{A}(\mathrm{alone})}+\frac{\mathrm{MI}C_{B}(\mathrm{combined})}{\mathrm{MI}C_{B}(\mathrm{alone})}$$

FICI interpretive criteria: FICI ≤ 0.5, synergy; 0.5 < FICI ≤ 1, additivity; 1 < FICI ≤ 4, indifference; FICI > 4, antagonism (Mhlongo et al. 2023).

## 3. Results and discussion

### 3.1. Strain identification

**Figure 2.**
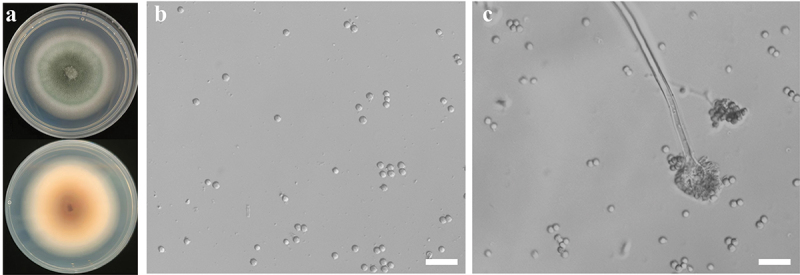
The morphological characters of *Aspergillus sydowii* LF51. (a) Colony morphology. (b) Spore morphology. (c) Sporophore morphology. Scale bars: 10 µm.

**Figure 3.**
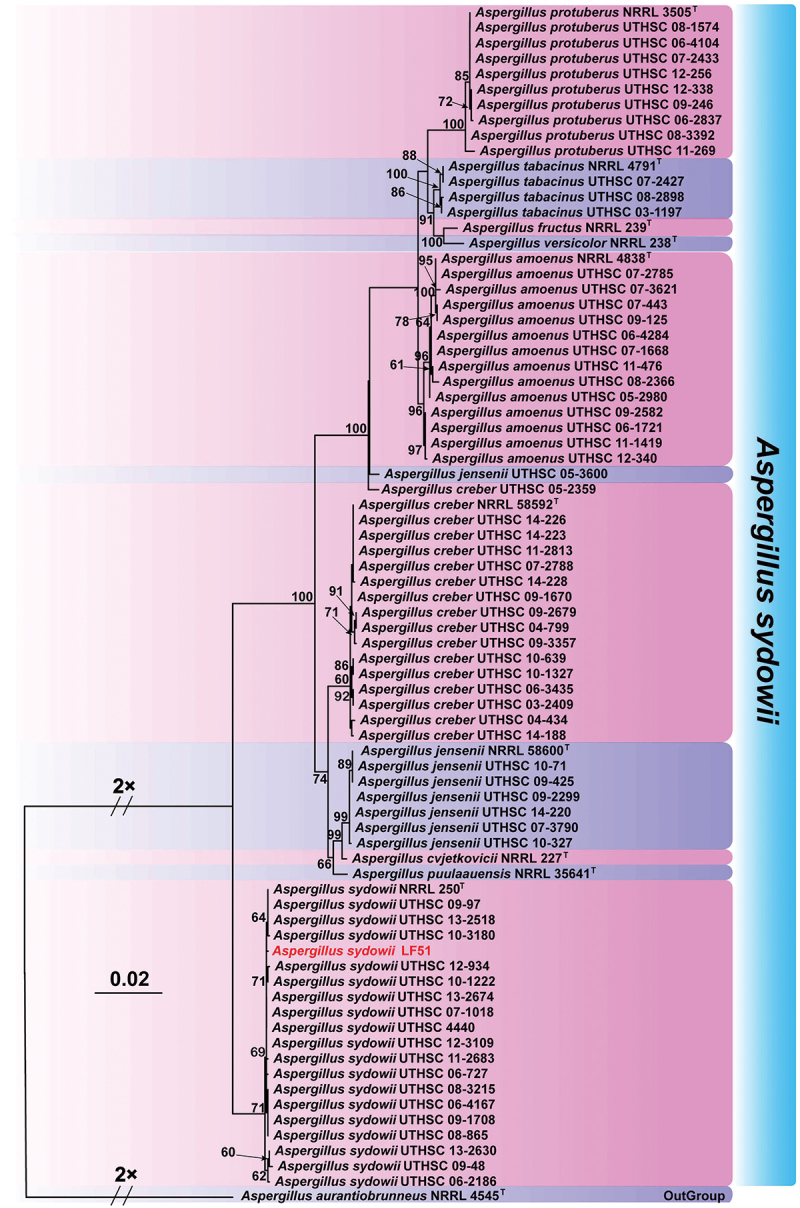
Maximum likelihood tree of *Aspergillus* section *sydowii* based on ITS, CaM, BenA, and RPB2 sequences. Maximum likelihood bootstrap (MLBP) support values above 50% are presented at the nodes and type strains are shown as ^T^. The tree was rooted to *A. aurantiobrunneus* NRRL 4545. The strain LF51 is printed in red font.

Based on morphological analysis (Figure 2), the stipes of strain LF51 observed under an optical microscope ranged from several tens of micrometres to 500 μm in length, with relatively thick and smooth walls. The vesicles appeared small and nearly spherical or slightly elliptical, while the conidia were spherical or elliptical with smooth surfaces. These morphological characteristics exhibited close alignment with those documented for *A. sydowii* (Ein-Gil et al. 2009). The ITS sequence of strain LF51 was subjected to a BLASTn search in the NCBI database, confirming its classification within the genus *Aspergillus*. A phylogenetic tree constructed from a combination of four genes (ITS, CaM, BenA, and RPB2) placed strain LF51 in a clade with other *A. sydowii* strains without distinct branching (Figure 3). Therefore, based on both morphological and molecular evidence, strain LF51 was conclusively identified as *A. sydowii*.

### 3.2. SMART

**Figure 4.**
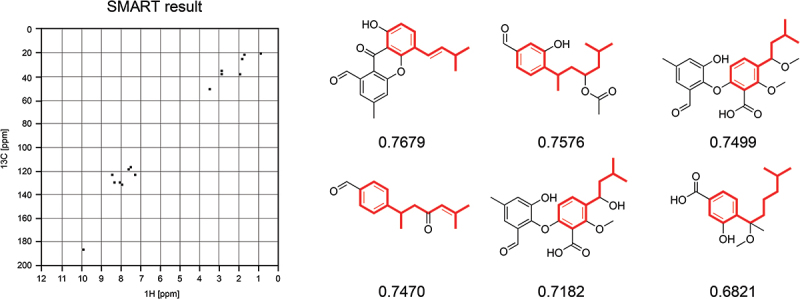
The result of analysis utilized SMART.

The HSQC spectrum of the crude extract from *A. sydowii* LF51 was acquired and subsequently exported as a CSV file for analysis using the SMART system (https://smart.ucsd.edu/classic) for preliminary structural classification. Analysis by SMART generated structural hypotheses, indicating that the top six compounds, ranked by cosine similarity scores, featured the characteristic partial structure of bisabolane-type sesquiterpenoids (Figure 4). These results suggested that the crude extract is enriched in bisabolane-type sesquiterpenoid derivatives.

### 3.3. Molecular networking

**Figure 5.**
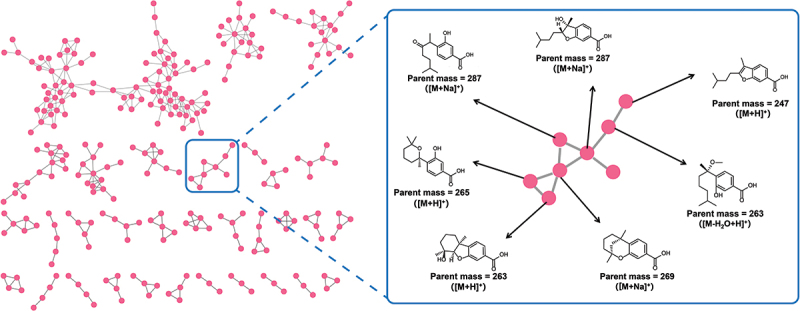
The result of analysis utilized molecular networking.

The secondary mass spectrometry data of the crude extract of *A. sydowii* LF51 was obtained, and a visual molecular network was constructed according to the GNPS website and Cytoscape software to guide the isolation of compounds. As shown in Figure 5, the annotated node was identified as a sesquiterpenoid, from which we isolated a series of analogs containing three new compounds.

### 3.4. Structural elucidation

**Figure 6.**
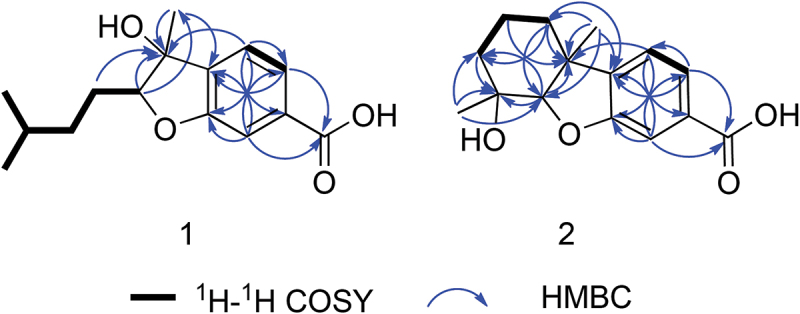
Structures, HMBC, and ^1^H-^1^H COSY correlations for compounds **1** and **2**.

The racemic form of aspersydonol A (**1**), isolated as a pale yellow oil, exhibited high-resolution electrospray ionisation mass spectrometry (HRESIMS) data with an [M – H_2_O + H]^+^ ion at *m*/*z* 247.1333 (calcd. C_15_H_19_O_3_ for 247.1329), corresponding to the molecular formula C_15_H_20_O_4_ (6 degrees of unsaturation). IR absorptions at *ν*_max_ 3,368 (O-H), 1,693 (C = O), and 1,592 cm^−1^ (aromatic C = C) indicated hydroxyl, carbonyl, and benzene functionalities. Analysis of the 1D NMR and HSQC data (Table 1) showed three singlet methyls (*δ*_C/H_ 22.9/0.96; 22.8/0.95; 25.6/1.62), two sp^3^ methylenes (*δ*_C/H_ 27.1/1.86; 36.3/1.56, 1.45), and five methines. The methines included three aromatic protons (*δ*_C/H_ 111.6/7.36; 124.3/7.43; 123.1/7.61), one oxygenated sp^3^ methine (*δ*_C/H_ 93.6/4.25), and one sp^3^ methine (*δ*_C/H_ 29.0/1.66). Additionally, five tertiary carbons including one carbonyl carbon (*δ*_C_ 167.5), three aromatic carbons (*δ*_C_ 133.1, 139.8, and one oxygenated carbon at *δ*_C_ 160.1), and one oxygenated sp^3^ carbon (*δ*_C_ 77.1) were observed. The NMR data accounted for all signals, with the exception of two hydroxyl groups that were not observed. The six degrees of unsaturation in compound **1** indicated a bicyclic architecture containing a benzene ring. The planar structure of compound **1** was definitively resolved through a comprehensive 2D NMR analysis (Figure 6). HMBC correlations of H-2 to C-1, C-4, C-6, and the carbonyl carbon C-14 (*δ*_C_ 167.5), from H-4 to C-2, C-6, and C-14, and from H-5 to C-1, C-3, and C-4, together with ^1^H-^1^H COSY correlation of H-4/H-5 revealed the presence of trisubstituted benzene ring with the carbonyl carbon C-14 positioned at C-3. Furthermore, HMBC correlations from H-5 and H-9 to the tertiary sp^3^ carbon C-7 (*δ*_C_ 77.1), from H-8 to C-7 and C-15, and from H_3_-15 to C-6, C-7, and C-8, combined with the ^1^H-^1^H COSY correlation of H-8/H_2_-9/H_2_-10/H-11/H_3_-13 and of H-11/H_3_-12 permitted the construction of the methylheptan-2-yl group located at the C-6 position of the benzene ring. Considering the chemical shifts of C-1 (*δ*_C_ 160.1) and C-8 (*δ*_C_ 93.6) and the requirement of unsaturation, C-1 and C-8 should be connected to the same oxygen atom to form the benzofuran core structure. Two unobserved hydroxyl groups were located at C-7 and C-14 by default, which was supported by the chemical shifts of C-7 (*δ*_C_ 77.1) and C-14 (*δ*_C_ 167.5) and the molecular formula of **1**. Thus, the planar structure of **1** was established as a phenolic bisabolane-type sesquiterpenoid with the benzofuran subunit as shown (Figure 1).

**Figure 7.**
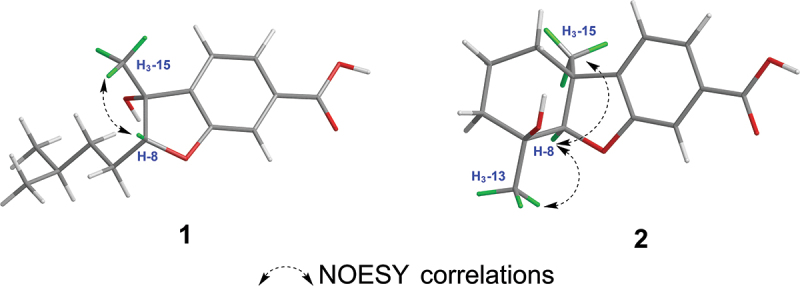
NOESY correlations for compounds **1** and **2**.

**Figure 8.**
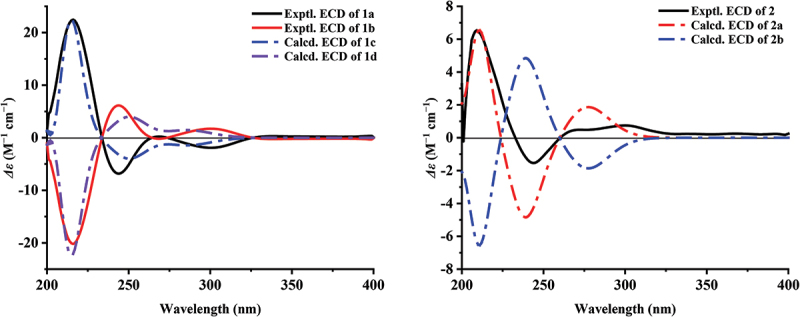
Calculated and experimental ECD spectra for compounds **1** and **2**.

NOESY correlation of H-8 with H_3_-15 (Figure 7) revealed these protons were cofacial, and the relative configuration was determined as shown. The optical inactivity (${\left[\alpha \right]}_{D}^{25}$ = 0, *c* 0.1, CH_3_OH) and the lack of distinct Cotton effects in its ECD spectrum revealed the racemic nature of compound **1**. Subsequently, compound **1** was separated *via* chiral HPLC (Kromasil 5-CelluCoat column, 250 × 4.6 mm, 5 μm) under isocratic conditions (53% acetonitrile, 0.35 mL/min) to obtain one pair of enantiomers with opposing optical rotations: **1a** (*t*_R_ = 12.5 min; ${\left[\alpha \right]}_{D}^{25}$ = +70.0, *c* 0.1, CH_3_OH) and **1b** (*t*_R_ = 13.5 min; ${\left[\alpha \right]}_{D}^{25}$ = −65.0, *c* 0.1, CH_3_OH). In addition, compounds **1a** and **1b** show mirror-image Cotton effects in the ECD spectra. The absolute configurations were determined by comparing the experimental ECD spectra with those of time-dependent density functional theory (TDDFT)-calculated spectra for two possible stereoisomers (7*R*,8*R*)-**1** (**1c**) and (7*S*,8*S*)-**1** (**1d**) (Figure 8). The experimental spectrum of **1a** matched well with the calculated spectrum of (7*R*,8*R*)-**1** (**1c**), while the experimental spectrum of **1b** aligned well with that of (7*S*,8*S*)-**1** (**1d**), establishing the absolute configurations of **1a** and **1b** as 7*R*,8*R* and 7*S*,8*S*, respectively.

Aspersydonol B (**2**) was also obtained as a pale yellow oil. Its molecular formula was assigned to C_15_H_18_O_4_ based on the HRESIMS data at *m*/*z* 263.1276 [M + H]^+^ (calcd. for C_15_H_19_O_4_ *m*/*z* 263.1278), suggesting seven degrees of unsaturation. IR absorption bands at 1,589 cm^−1^ and 1,689 cm^−1^ indicated the presence of aromatic and carbonyl functionalities. Analysis of its 1D NMR (Table 1) data revealed the following structural moietys, two methyls, three methylenes, one oxygenated methine, one sp^3^ quaternary carbon, one oxygenated sp^3^ tertiary carbon, six aromatic carbons (three protonated), and one carbonyl carbon (*δ*_C_ 167.9), accounting for five degrees of unsaturation. These data corresponded for all NNR signals except for two unobserved hydroxyl groups, suggesting the tricyclic nature of **2**. Interpretation of 2D NMR data (Figure 6) also established the same benzofuran ring as that of **1**. However, the remaining structure was varied. ^1^H-^1^H COSY correlation of H₂-10/H₂-11/H₂-12, along with HMBC correlations from H-11 to C-7, from H-12 to C-6, C-7, C-8, and C-15, and from H_3_-15 to C-6, C-7, C-8, and C-12 constructed the propan-1-yl moiety (C-10–C-12) with both C-12 and C-15 attached directly to C-7 of benzofuran ring. Furthermore, HMBC cross-peaks from H-8 to C-9, C-10, and C-13, from H-10 to C-8 and C-9, and from H_3_-13 to C-8, C-9, and C-10 suggested that all three carbons C-8, C-10, and C-13 linked to C-9, establishing a cyclohexane ring fused to the benzofuran scaffold at the C-7 and C-8 positions to form the hexahydrodibenzo[*b,d*]furan skeleton. Considering the chemical shifts of C-9 (*δ*_C_ 94.6) and C-14 (*δ*_C_ 167.9), the unobserved hydroxyl groups were located at C-9 and C-14 by default. Therefore, the planar structure of **2** was established as shown (Figure 1). Compound **2** represents the first example of natural PBS with a hexahydrodibenzo[*b,d*]furan skeleton.

The relative configuration of compound **2** was established through NOESY analysis (Figure 7). NOESY correlations of H-8 with H₃-13 and H₃-15 placed these protons on the same side of the ring system. The absolute configuration of **2** was assigned by comparison of the experimental ECD spectra of **2** with those of calculated ECD spectra for one pair of enantiomers (7*S*,8*R*,9*S*)-**2** (**2a**) and (7*R*,8*S*,9*R*)-**2** (**2b**) (Figure 8). The experimental spectrum of **2** matched well with the calculated ECD spectrum of (7*S*,8*R*,9*S*)-enantiomer (**2a**), defining the absolute configuration as 7*S*,8*R*,9*S*.

**Table 1.** ^1^H NMR and ^13^C NMR data for **1** and **2** (in acetone‑*d*_*6*_).

Pos.	1a/1b		2	
	*δ*_C_, mult.	*δ*_H_ (*J* in Hz)	*δ*_C_, mult.	*δ*_H_ (*J* in Hz)
1	160.1, qC		159.0, qC	
2	111.6, CH	7.36 (s, 1H)	111.2, CH	7.36 (s, 1H)
3	133.1, qC		131.5, qC	
4	123.1, CH	7.61 (d, 7.7, 1H)	123.8, CH	7.61 (d, 7.5, 1H)
5	124.3, CH	7.43 (d, 7.7, 1H)	122.4, CH	7.23 (d, 7.5, 1H)
6	139.8, qC		146.3, qC	
7	77.1, qC		44.3, qC	
8	93.6, CH	4.25 (t, 6.8, 1H)	94.9, CH	3.96 (s, 1H)
9	27.1, CH_2_	1.86 (dt, 8.0, 5.4, 2H)	69.6, qC	
10	36.3, CH_2_	1.56 (m, 1H)	34.8, CH_2_	1.56 (td, 11.7, 4.4, 1H)
		1.45 (m, 1H)		1.65 (dt, 13.6, 4.0, 1H)
11	29.0, CH	1.66 (m, 1H)	17.7, CH_2_	1.31 (dt, 13.1, 4.3, 1H)
				1.84 (m, 1H)
12	22.8, CH_3_	0.95 (d, 6.8, 3H)	36.8, CH_2_	1.23 (td, 12.6, 4.0, 1H)
				1.71 (dt, 13.4, 4.2, 1H)
13	22.9, CH_3_	0.96 (d, 6.6, 3H)	29.2, CH_3_	1.40 (s, 3H)
14	167.5, qC		167.9, qC	
15	25.6, CH_3_	1.62 (s, 3H)	22.7, CH_3_	1.53 (s, 3H)

500 MHz for ^1^H NMR, 125 MHz for ^13^C NMR.

Spectroscopic comparison with previously reported data enabled the identification of the known compounds: aspergillusene B (**3**) and (+)-(7*S*)-7-*O*-methylsydonic acid (**9**) (Trisuwan et al. 2011); (±)-sydowic acid (**4**) (Hamasaki et al. 1975); (+)-penispidin B (**5**) (Yin et al. 2021); 7-deoxy-7,8-didehydrosydonic acid (**6**) and 7-deoxy-7,14-didehydrosydonic acid (**11**) (Wei et al. 2010); (*E*)-7-deoxy-7,8-didehydro-12-hydroxysydonic acid (**7**) (Wang et al. 2018), (+)-sydonic acid (**8**) (Sun et al. 2021), (–)-phomoterpene A (**10**) (Qu et al. 2020), (*E*)-3-(3-hydroxy-4-(6-methylhept-2-en-2-yl)benzyl)-5-methylbenzene-1,2-diol (**12**) (Wu et al. 2018), (7*S*,11*S*)-(+)-12-hydroxysydonic acid (**13**) (Zhang et al. 2020), and (–)-hydroxysydonic acid (**14**) (Bunbamrung et al. 2015).

### 3.5. Antifungal activities

All compounds were tested for their antifungal effects against six strains of *C. gattii* (3284G14, R265, 3291, 3271G1, R272, and WM276) and *C. neoformans* H99, as summarised in Table 2. Compounds **3**, **6**, and **11** demonstrated moderate inhibitory activity against *C. gattii* variants 3284G14, R265, and 3271G1, with the same MIC value of 64 μg/mL. Meanwhile, compound **6** also exhibited antifungal activity against *C. gattii* R272 (MIC = 64 μg/mL). Additionally, compound **9** displayed moderate antifungal activities against *C. gattii* R265, 3291, and 3271G1 with MICs of 32, 64, and 64 μg/mL, respectively. In comparison, the reference antifungals fluconazole and amphotericin B (AmB) showed comparatively lower MICs at 8 and 1 μg/mL, correspondingly.

**Table 2.** Antifungal activities for compounds **1**−**14**.

Compounds	*Cryptococcus gattii*						*C. neoformans*
	3284G14	R265	3291	3271G1	R272	WM276	H99
**MIC (μg/mL)**							
**1**, **2**	>64	>64	>64	>64	>64	>64	>64
**3**	64	64	>64	64	>64	>64	>64
**4**, **5**	>64	>64	>64	>64	>64	>64	>64
**6**	64	64	>64	64	64	>64	>64
**7**, **8**	>64	>64	>64	>64	>64	>64	>64
**9**	>64	32	64	64	>64	>64	>64
**10**	>64	>64	>64	>64	>64	>64	>64
**11**	64	64	>64	64	>64	>64	>64
**12**–**14**	>64	>64	>64	>64	>64	>64	>64
Fluconazole	8	8	8	8	8	8	8
Amphotericin B	1	1	1	1	1	1	1

**Figure 9.**
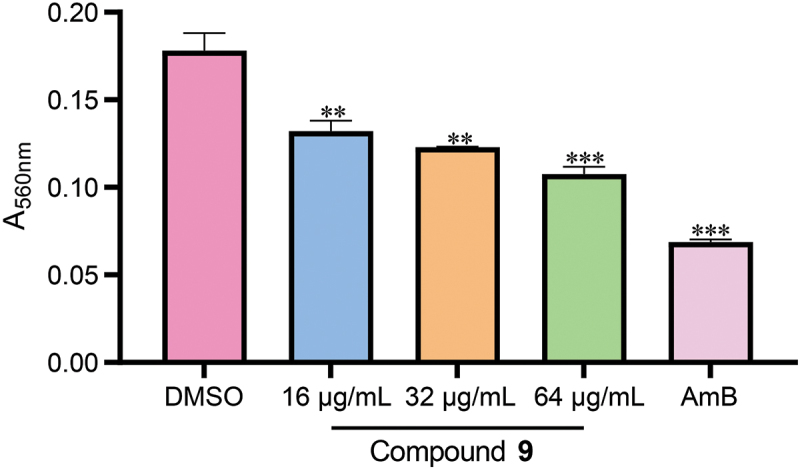
Inhibition of *Cryptococcus gattii* R265 urease activity by compound **9** (**p < 0.01 vs. DMSO group, ***p < 0.001 vs. DMSO group).

Chequerboard assays were performed to evaluate the synergistic interactions of compounds **3**, **6**, **9**, and **11** with AmB or FLC against *C*. *gattii* R265. All compounds exhibited additive effects when combined with AmB, with fractional inhibitory concentration index (FICI) values of 0.75, 0.56, 1.0, and 0.56, respectively (Table 3). In addition, compound **9** also showed an additive interaction with FLC (FICI = 0.56) (Table 3). Previous studies suggest that urease-derived ammonia may promote *C. gattii* migration into the central nervous system by damaging host endothelial cells (Singh et al. 2013). To evaluate the inhibitory potential of compound **9** on urease activity, intracellular urease assays were performed on *C. gattii* R265. The results revealed a concentration-dependent inhibition of urease activity by compound **9**, with progressive suppression observed as its concentration increased (Figure 9).

**Table 3.** Combined use of AmB or FLC with the compounds (MIC, μg/mL).

Compounds (A)	Antifungal agents (B)	MIC_A_ (combined)	MIC_B_ (combined)	FICI	Mode
**3**	AmB	32	0.25	0.75	Additivity
	FLC	8	16	2.12	Indifference
**6**	AmB	4	0.5	0.56	Additivity
	FLC	8	8	1.12	Indifference
**9**	AmB	16	0.5	1.00	Additivity
	FLC	2	4	0.56	Additivity
**11**	AmB	4	0.5	0.56	Additivity
	FLC	4	8	1.06	Indifference

**Figure 10.**
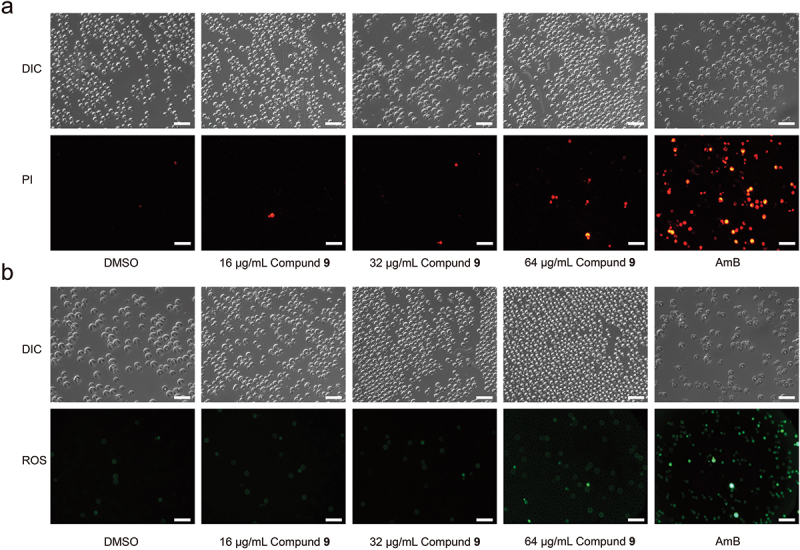
Antifungal mechanism of compound **9** against *Cryptococcus gattii* R265. (a) Compound **9** increases cell membrane permeability in *C. gattii* R265 (DIC microscopy; PI staining). (b) Compound **9** induces intracellular ROS production in *C. gattii* R265 (400× magnification). Scale bars: 10 μm.

To further elucidate the antifungal mechanism of compound **9**, membrane integrity and reactive oxygen species (ROS) levels were analysed using propidium iodide (PI) and ROS-specific fluorescent dyes. Fluorescence microscopy revealed dose-dependent increases in red fluorescence (PI staining) after treatment with 32 μg/mL and 64 μg/mL of compound **9**, indicating compromised membrane permeability and leakage of cellular contents (Figure 10). Concurrently, a significant increase in green fluorescence (ROS staining) was detected at 64 μg/mL, suggesting intracellular oxidative stress induction (Figure 10). These results collectively demonstrate that compound **9** exhibits its antifungal activity against *C. gattii* R265 by suppressing urease activity, disrupting membrane integrity and inducing oxidative damage *via* ROS accumulation. It exhibited additive effects when combined with AmB or FLC against *C*. *gattii* R265. These additive effects could be attributed to the multi-target action of compound **9**, which complements the distinct mechanisms of both AmB and FLC to enhance antifungal activity.

### 3.6. Antibacterial activities

Furthermore, the antimicrobial screening encompassed compounds **1**−**14** against three strains of human pathogen, including *Escherichia coli*, *Mycobacterium smegmatis*, and methicillin-resistant *Staphylococcus aureus* (MRSA), along with four strains of plant pathogen, including *Pseudomonas syringae* pv. lachrymans (Psl), *Bacillus subtilis*, *Xanthomonas campestris* pv. campestris (Xcc), and *Ralstonia solanacearum* (Table 4). Among the tested compounds, compound **3** displayed selective growth inhibition against *M. smegmatis* (MIC = 64 μg/mL), while the reference antibiotic vancomycin exhibited superior potency with an MIC of 8 μg/mL.

**Table 4.** Antibacterial activities for compounds **1**−**14**.

Compounds	*Escherichia coli*	MRSA	*Mycobacterium smegmatis*	*Bacillus subtilis*	Psl	*Bacillus solanacearum*	Xcc
**MIC (μg/mL)**							
**1**, **2**	>64	>64	>64	>64	>64	>64	>64
**3**	>64	>64	64	>64	>64	>64	>64
**4**–**14**	>64	>64	>64	>64	>64	>64	>64
Vancomycin	>64	4	8	2	>64	1	8

## 4. Conclusions

In conclusion, three new PBS derivatives (±)-aspersydonol A (**1a**/**1b**) and aspersydonol B (**2**), along with 12 known analogues, were obtained from the fungus *A. sydowii* LF51 using a combination of molecular networking and SMART approaches. These PBS compounds feature a bisabolane core adopting monocyclic (e.g. compounds **6**−**14**), bicyclic (e.g. compounds **1**, **3**−**5**), or tricyclic (compound **2**) core skeletons, accompanied by distinct phenolic hydroxylation patterns. The phenolic moieties originate from direct hydroxylation of aromatic rings, forming classical phenolic architectures. Oxidative post-modifications, including epoxide groups, carbonyl functionalities (ketones, carboxylic acids, and aldehydes), and *α,β*-unsaturated ketone systems, further amplify their structural complexity. In particular, compound **2** represents the first example of natural PBS with a hexahydrodibenzo[*b,d*]furan structural framework. Bioactivity evaluation revealed that compounds **3**, **6**, **9**, and **11** exhibited moderate anti-*Cryptococcus* activity with additive effects when combined with AmB against *C. gattii* R265. Furthermore, compound **9** inhibited *C. gattii* R265 by inhibition of virulence-associated urease, disruption of membrane integrity, and ROS accumulation. In the future, structure-activity relationship studies and structural optimisation should be conducted to improve antifungal effects. Detailed mechanistic investigations to identify molecular targets and exploration of combination mechanisms with antifungal drugs should also be performed. These findings not only expand the chemical space of PBS compounds but also broaden their scope of activity, providing promising lead compounds for the development of anti-*Cryptococcus* drugs.

## Supplementary Material

0810-Supplemental material.docx
