# Supplementary material for: New phenolic bisabolane sesquiterpenoids discovered from the marine-derived fungus Aspergillus sydowii assisted by molecular networking and SMART strategies
Source: Mycology. 2025 Sep 14;17(2):552–64. doi: 10.1080/21501203.2025.2547630 (PMC13267026; doi:10.1080/21501203.2025.2547630)
Supplement: 0810-Supplemental material.docx [file TMYC_A_2547630_SM8886.docx]

Supplemental material

**New** **phenolic bisabolane sesquiterpenoids discovered from the marine-derived fungus** ***Aspergillus sydowii* assisted by molecular networking and SMART strategies**

Baodan Zhang^a,b, #^, Meiyan Bao^a,b, #^, Ling Liu^a,b,*^

^a^State Key Laboratory of Microbial Diversity and Innovative Utilization, Institute of Microbiology, Chinese Academy of Sciences, Beijing, China

^b^University of Chinese Academy of Sciences, Beijing, China

^#^These authors contributed equally to this work.

^*^ Corresponding author:

Ling Liu liul@im.ac.cn

State Key Laboratory of Microbial Diversity and Innovative Utilization, Institute of Microbiology, Chinese Academy of Sciences, Beijing 100101, China

**Table of contents**

| 1. **Supplementary methods** |  |
| --- | --- |
| 1.1. Antibacterial assay | S1 |
| 1.2. Anti-fungal assay | S1 |
| 1.3. Urease activity assay | S1 |
| 1.4. Cell membrane permeability assay | S1 |
| 1.5. Reactive oxygen species (ROS) generation assay | S2 |
| 1. **Supplementary tables** |  |
| **Table S1.** The reagents and instruments used in the present study. | S3 |
| **Table S2.** The culture media used for the fermentation of *Aspergillus sydowii* LF51. | S4 |
| **Table S3.** The fermentation condition for the fermentation of *Aspergillus sydowii* LF51. | S5 |
| 1. **Supplementary figures** |  |
| **Figure S1.** The HPLC profilings of EtOAc extracts from different culture media for *Aspergillus* *sydowii* LF51 at 254 nm (no sea salt, long-term fermentation). | S6 |
| **Figure S2.** The HPLC profilings of EtOAc extracts from different culture media for *Aspergillus sydowii* LF51 at 254 nm (no sea salt, short-term fermentation). | S6 |
| **Figure** **S3.** The HPLC profilings of EtOAc extracts from different culture media for *Aspergillus sydowii* LF51 at 254 nm (with sea salt, short-term fermentation). | S7 |
| **Figure** **S4.** The HPLC profilings of EtOAc extracts from different culture media for *Aspergillus sydowii* LF51 at 254 nm (with sea salt, long-term fermentation). | S7 |
| **Figure S5.** ^1^H NMR spectrum of aspersydonol A (**1**; 500 MHz, acetone-*d*_6_). | S8 |
| **Figure S6.** ^13^C NMR spectrum of aspersydonol A (**1**; 125 MHz, acetone-*d*_6_). | S8 |
| **Figure S7.** ^1^H-^1^H COSY spectrum of aspersydonol A (**1**; 500 MHz, acetone-*d*_6_). | S9 |
| **Figure S8.** HSQC spectrum of aspersydonol A (**1**; 500 MHz, acetone-*d*_6_). | S9 |
| **Figure S9.** HMBC spectrum of aspersydonol A (**1**; 500 MHz, CDCl_3_). | S10 |
| **Figure S10.** ROESY spectrum of aspersydonol A (**1**; 500 MHz, acetone-*d*_6_). | S10 |
| **Figure S11.** ^1^H NMR spectrum of aspersydonol B (**2**; 500 MHz, acetone-*d*_6_). | S11 |
| **Figure S12.** ^13^C NMR spectrum of aspersydonol B (**2**; 125 MHz, acetone-*d*_6_). | S11 |
| **Figure S13.** ^1^H-^1^H COSY spectrum of aspersydonol B (**2**; 500 MHz, acetone-*d*_6_). | S12 |
| **Figure S14.** HSQC spectrum of aspersydonol B (**2**; 500 MHz, acetone-*d*_6_). | S12 |
| **Figure S15.** HMBC spectrum of aspersydonol B (**2**; 500 MHz, acetone-*d*_6_). | S13 |
| **Figure S16.** ROESY spectrum of aspersydonol B (**2**; 500 MHz, DMSO-*d*_6_). | S13 |
| **Figure S17.** ECD conformers of aspersydonol A (**1**). | S14 |
| **Figure S18.** ECD conformers of aspersydonol B (**2**). | S20 |
|  |  |

| **1. Supplementary methods**  ***1.1. Antibacterial assay***  For antibacterial activity, bacterial inocula were prepared from colonies grown on LB (1.0% tryptone, 0.5% yeast extract, 1.0% tryptone, and 2.0% agar) plates, at final concentrations of 5 × 10^4^ to 1 × 10^5^ CFU/mL. Briefly, 100 μL of compounds (5 to 160 μg/mL in LB liquid medium with DMSO concentration of 1%) were added to a 96-well plate containing 100 μL of bacterial suspension, and incubated at 37 ℃ for 24 h. Minimal inhibitory concentration (MIC) values were defined as the lowest drug concentration that showed no bacterial growth. These experiments were performed in triplicate and repeated three times. Vancomycin (Mreda, Beijing, Lot 02105281) was used as the positive control. DMSO and LB liquid medium were used as the negative and blank controls.  ***1.2. Anti-fungal assay***  For anti-fungal activity, fungal inocula were prepared from colonies grown on YPD (2.0% glucose, 2.0% malt extract, 1.0% yeast extract, and 2.0% agar) plates, at final concentrations of 2 × 10^3^ to 5 × 10^3^ cfu/mL. Briefly, 100 μL of compounds (2 to 64 μg/mL in RPMI 1640 medium with DMSO concentration of 1%) were added to a 96-well plate containing 100 μL of fungal suspension, and incubated at 37 ℃ for 72 h. MIC values were defined as the lowest drug concentration that showed no yeast growth. These experiments were performed in triplicate and repeated three times. Amphotericin B (AmB, Mreda, Beijing, Lot 02105280) was used as the positive control. DMSO and RPMI 1640 medium were used as the negative and blank controls, respectively.  ***1.3. Urease activity assay***  First, prepare urea medium (containing tryptone, NaCl, KH2PO4, glucose, 0.2% phenol red, pH adjusted to 7.2) and sterilize it by autoclaving at 115 °C for 20 min. Separately, prepare a 10% urea solution (filter-sterilized). Add 2 mL of the 10% urea solution to the sterilized urea medium and mix thoroughly to form the complete urea medium. Adjust the concentration of *Cryptococcus gattii* R265 cells to 1 × 10^4^ cfu/mL using the urea medium, and add the test compounds. Culture the mixture at 37 °C with 220 r/min shaking for 8 h. Collect an appropriate volume of the culture, centrifuge it, and measure the optical density (OD_560_). Use amphotericin B as the positive control and DMSO as the negative control. The experiment is performed in triplicate.  ***1.4. Cell membrane permeability assay***  Adjust the cell concentration of *C. gattii* R265 to 1 × 10^7^ cfu/mL using culture medium and add compound solutions at corresponding concentrations. Culture the mixture at 37°C with 220 r/min shaking for 8 h. Centrifuge the culture and discard the supernatant. Wash the cell pellets with PBS and centrifuge again (6,500 r/min, 5 min), then discard the supernatant. Resuspend the cells in 2 μg/mL propidium iodide (PI) staining solution and incubate for 15 min. After staining, centrifuge (6,500 r/min, 5 min) to remove the supernatant and wash the cells three times with PBS. Capture images using a fluorescence microscope. Amphotericin B was used as the positive control, and DMSO as the negative control. The experiment was performed in triplicate.  ***1.5. Reactive oxygen species (ROS) generation assay***  Adjust the cell concentration of *C. gattii* R265 to 1 × 10^7^ cfu/mL using culture medium and add compound solutions at corresponding concentrations. Culture the mixture at 37 °C with 220 r/min shaking for 8 h. Centrifuge the culture and discard the supernatant. Wash the cell pellets with PBS and centrifuge again (6,500 r/min, 5 min), then discard the supernatant. Resuspend the cells in DCFH-DA fluorescent probe solution and incubate at 37 °C for 30 min in the dark. During staining, invert the tubes gently every 5 min to ensure full contact between the probe and cells. After incubation, centrifuge (6,500 r/min, 5 min) to remove the supernatant and wash the cells three times with PBS to eliminate residual dye. Capture fluorescence images using a fluorescence microscope. Amphotericin B was used as the positive control, and DMSO as the negative control. The experiment was performed in triplicate. |  |
| --- | --- |

**2. Supplementary tables**

**Table S1.** The reagents and instruments used in the present study.

| **Reagents/Instruments** | **Specification/Company** |
| --- | --- |
| Anton Paar MCP 200 Automatic Polarimeter | Anton Paar, Graz, Austria |
| Thermo Genesys-10S UV/Vis spectrophotometer | Thermo Fisher Scientific, Waltham, MA, USA |
| JASCO J-815 spectropolarimeter | JASCO, Tsukuba, Japan |
| Nicolet IS5 FT-IR spectrophotometer | Thermo Fisher Scientific, Waltham, MA, USA |
| Bruker Avance-500 spectrometer | Bruker, Bremen, Germany |
| Agilent Accurate-Mass-Q-TOF LC/MS 6520 | Agilent Technologies, Santa Clara, CA, USA |
| Agilent 1260 instrument | Agilent Technologies Inc., CA, USA |
| YMC-pack ODS-A | 10 × 250 mm, 5 µm, 2 mL/min, YMC CO., LTD., Kyoto, Japan |
| Sephadex LH-20 | GE Healthcare, Uppsala, Sweden |
| Silica gel | Qingdao Marine Chemical Factory, Qingdao, China |
| Acetone-*d*_6_: *δ*_H_ 2.05/*δ*_C_ 29.8/206.3 | Cypress Technology Co., LTD (Beijing, China) |
| DMSO-*d*_6_: *δ*_H_ 2.50/*δ*_C_ 39.5 | Cypress Technology Co., LTD (Beijing, China) |

**Table S2.** The culture media used for the fermentation of *Aspergillus sydowii* LF51.

| **Media** | **Phase** | **Component** |
| --- | --- | --- |
| Rice | Solid | Rice 100 g, distilled H_2_O 105 mL |
| Oatmeal | Solid | Oatmeal 100 g, distilled H_2_O 105 mL |
| Fungus II | Liquid | Maltose extract 20 g, MSG 10 g, KH_2_PO_4_∙3H_2_O 0.5 g, MgSO_4_·7H_2_O 0.3 g, glucose 10 g, yeast extract 3 g, corn pulp 1 g, mannitol 20 g, distilled water 1 L |
| SDA | Liquid | Peptone 10 g, glucose 40 g, distilled water 1 L |
| CDA | Liquid | Sodium nitrate 3 g, K_2_HPO_4_ 1 g, MgSO_4_ 0.5 g, KCl 0.5 g, FeSO_4_ 0.01 g, sucrose 30 g, distilled water 1 L |
| YM | Liquid | Yeast extract 4 g, maltose extract 10 g, distilled water 1 L |
| YMG | Liquid | Glucose 4 g, yeast extract 4 g, maltose extract 10 g, distilled water 1 L |
| YPS | Liquid | Glucose 20 g, yeast extract 2 g, peptone 5 g, MgSO_4_ 0.5 g, KH_2_PO_4_ 1 g, distilled water 1 L |
| Martin | Solid | Glucose 10 g, peptone 5 g, MgSO_4_∙7H_2_O 0.5 g, KH_2_PO4∙3H_2_O 1 g, distilled water 1 L |
| GPY | Solid | Glucose 10 g, peptone 5 g, yeast extract 1 g, CaCO_3_ 1 g, distilled water 1 L |

**Table S3.** The fermentation condition for the fermentation of *Aspergillus sydowii* LF51.

| **Media** | **Culture condition** | **3% sea salt** | **Culture time** |
| --- | --- | --- | --- |
| Rice | 28 °C, away from light, let stand | Yes/no | 20 d/40 d |
| Oatmeal | 28 °C, away from light, let stand | Yes/no | 20 d/40 d |
| Fungus II | 28 °C, away from light, let stand | Yes/no | 20 d/40 d |
| SDA | 28 °C, away from light, let stand | Yes/no | 20 d/40 d |
| CDA | 28 °C, away from light, let stand | Yes/no | 20 d/40 d |
| YM | 28 °C, away from light, let stand | Yes/no | 20 d/40 d |
| YMG | 28 °C, away from light, let stand | Yes/no | 20 d/40 d |
| YPS | 28 °C, away from light, let stand | Yes/no | 20 d/40 d |
| Martin | 28 °C, away from light, let stand | Yes/no | 20 d/40 d |
| GPY | 28 °C, away from light, let stand | Yes/no | 20 d/40 d |

**3. Supplementary figures**

**
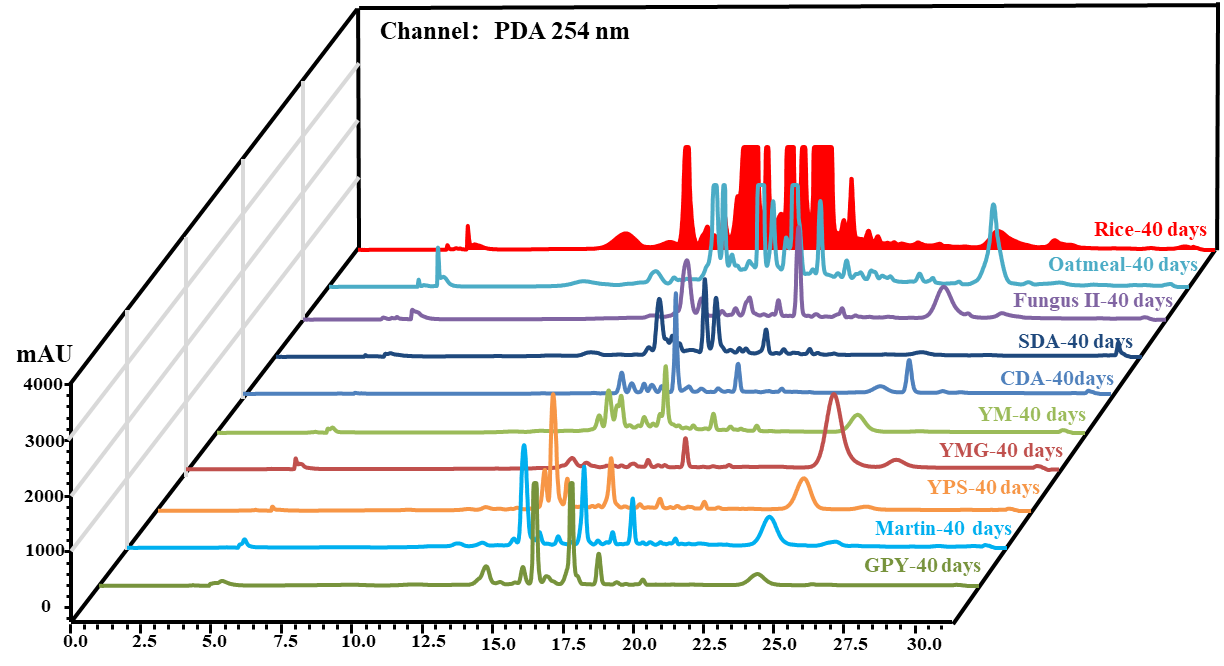
****Figure S1**. The HPLC profilings of EtOAc extracts from different culture media for *Aspergillus sydowii* LF51 at 254 nm (no sea salt, long-term fermentation).

**
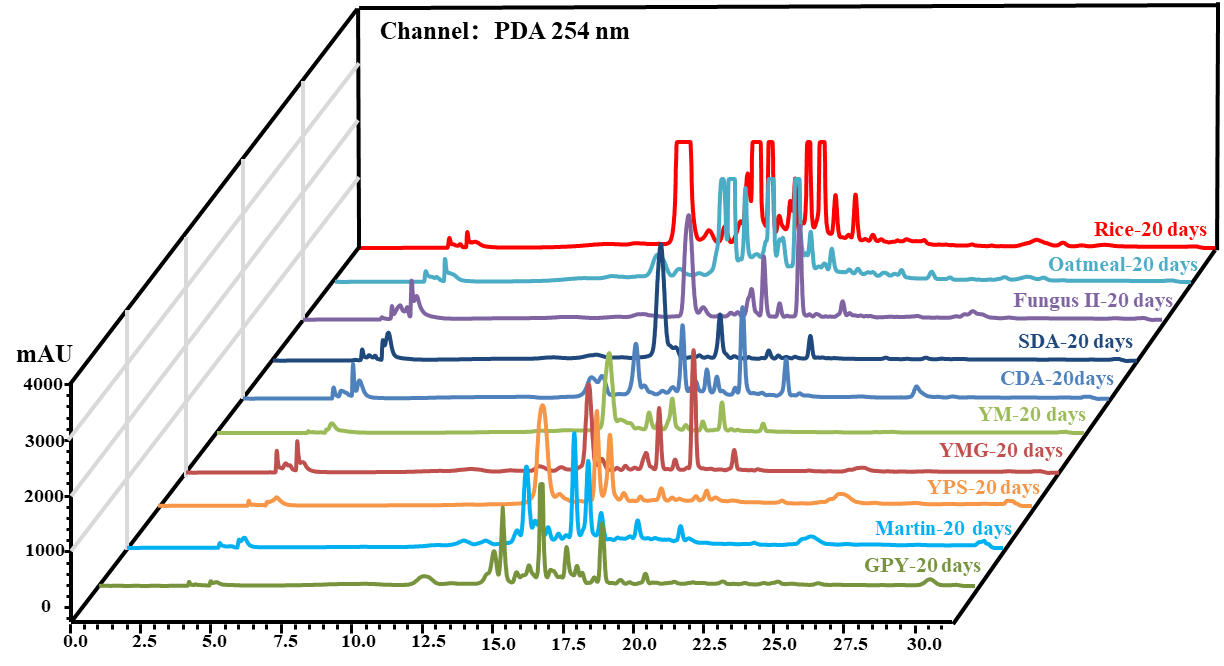
****Figure S2**. The HPLC profilings of EtOAc extracts from different culture media for *Aspergillus sydowii* LF51 at 254 nm (no sea salt, short-term fermentation).

**
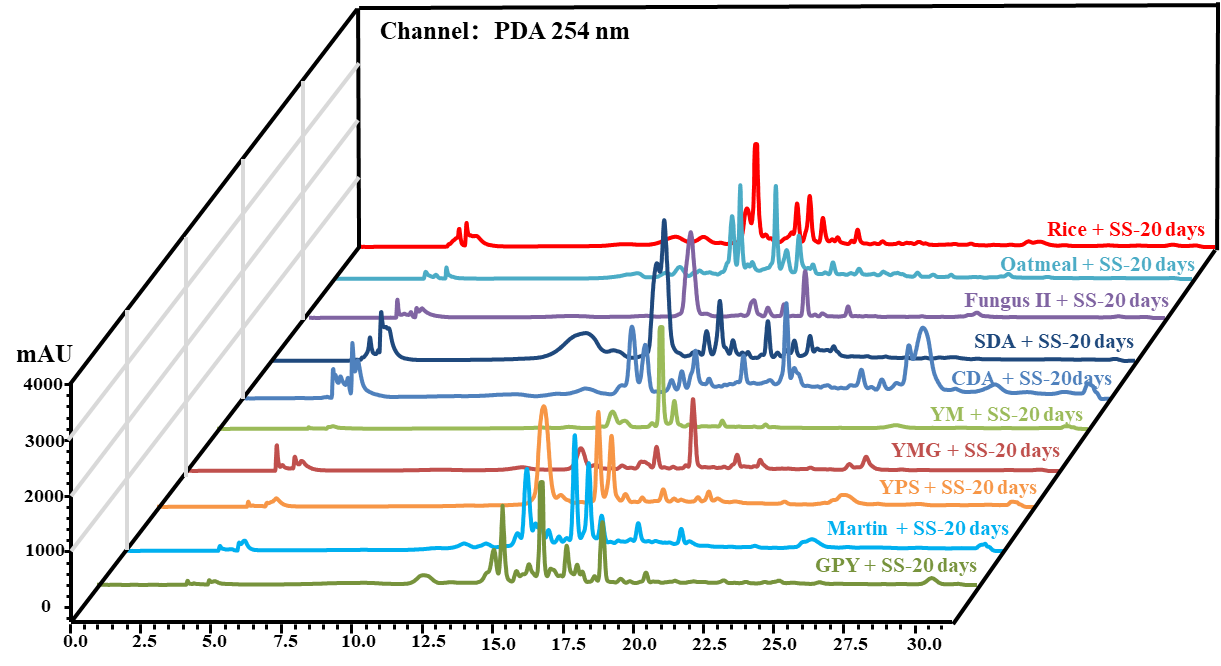
****Figure S3**. The HPLC profilings of EtOAc extracts from different culture media for *Aspergillus sydowii* LF51 at 254 nm (with sea salt, short-term fermentation).

**
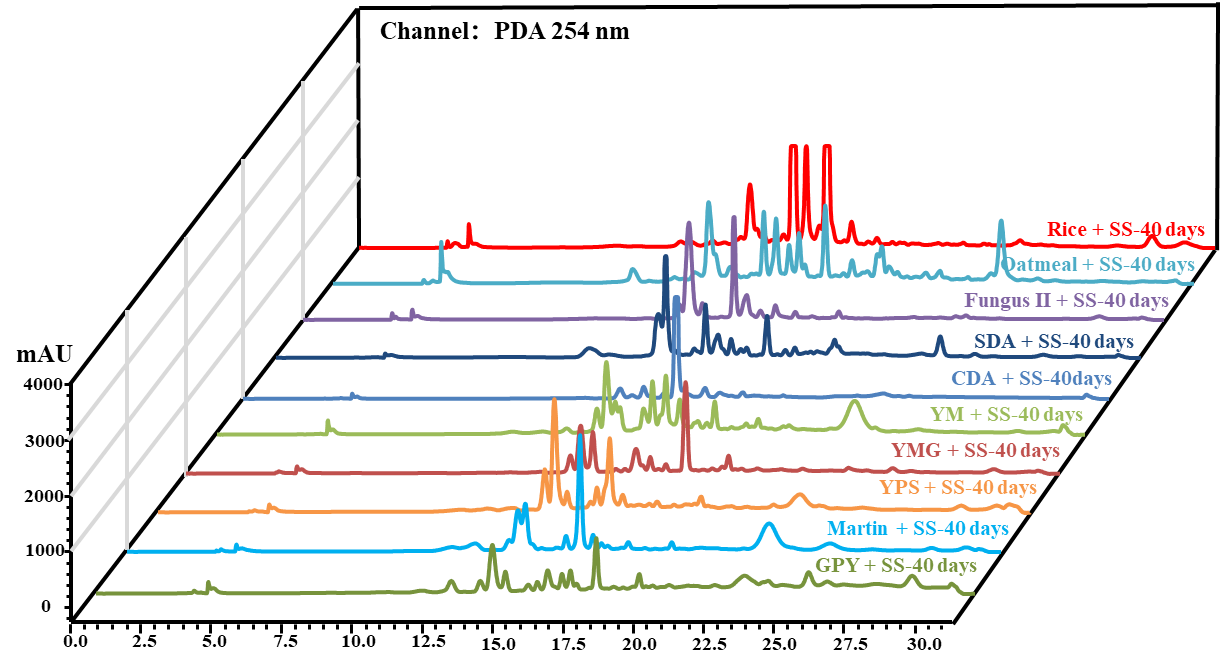
****Figure S4**. The HPLC profilings of EtOAc extracts from different culture media for *Aspergillus sydowii* LF51 at 254 nm (with sea salt, long-term fermentation).


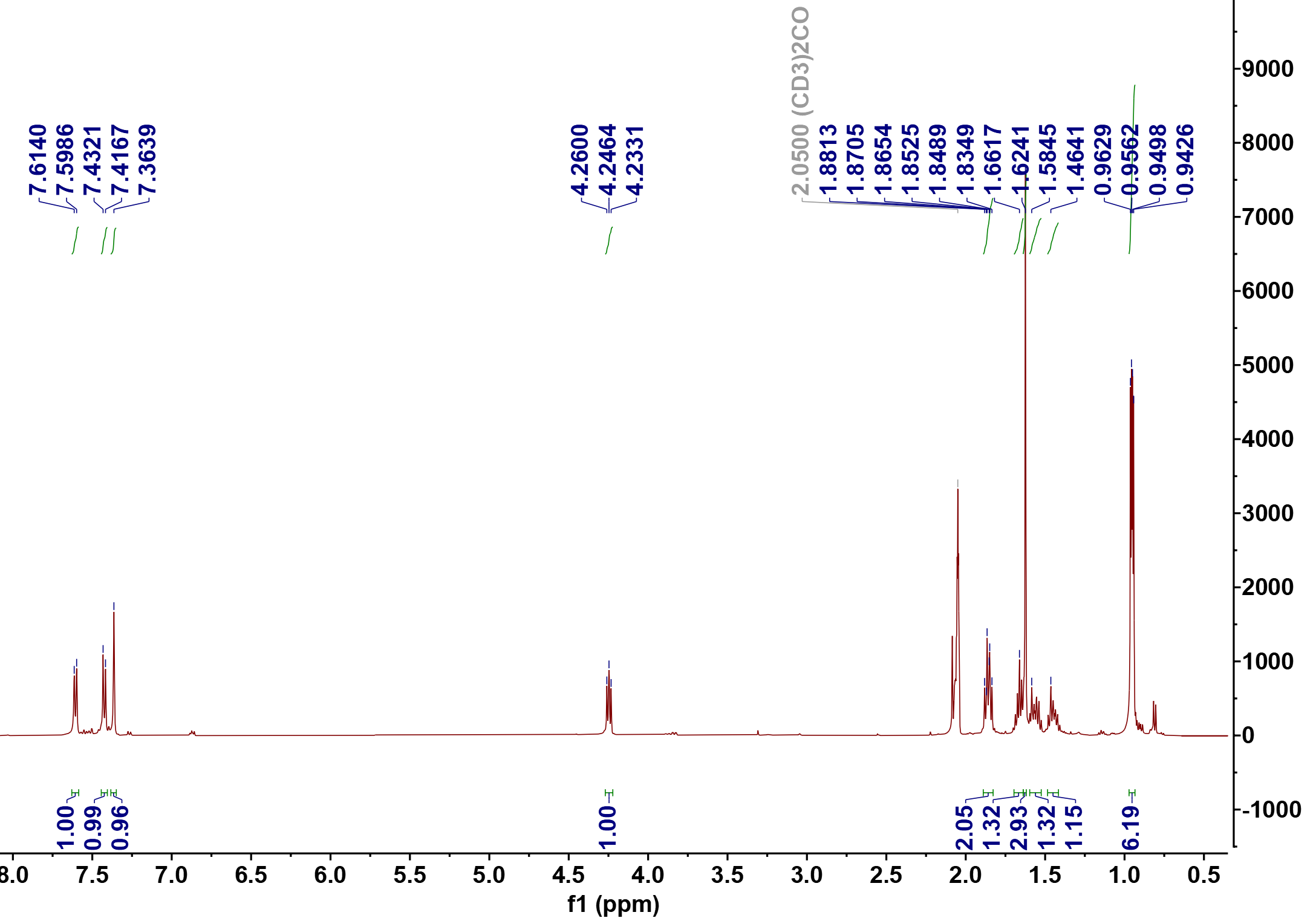


**Figure S5.** ^1^H NMR spectrum of aspersydonol A (**1**; 500 MHz, acetone-*d*_6_).


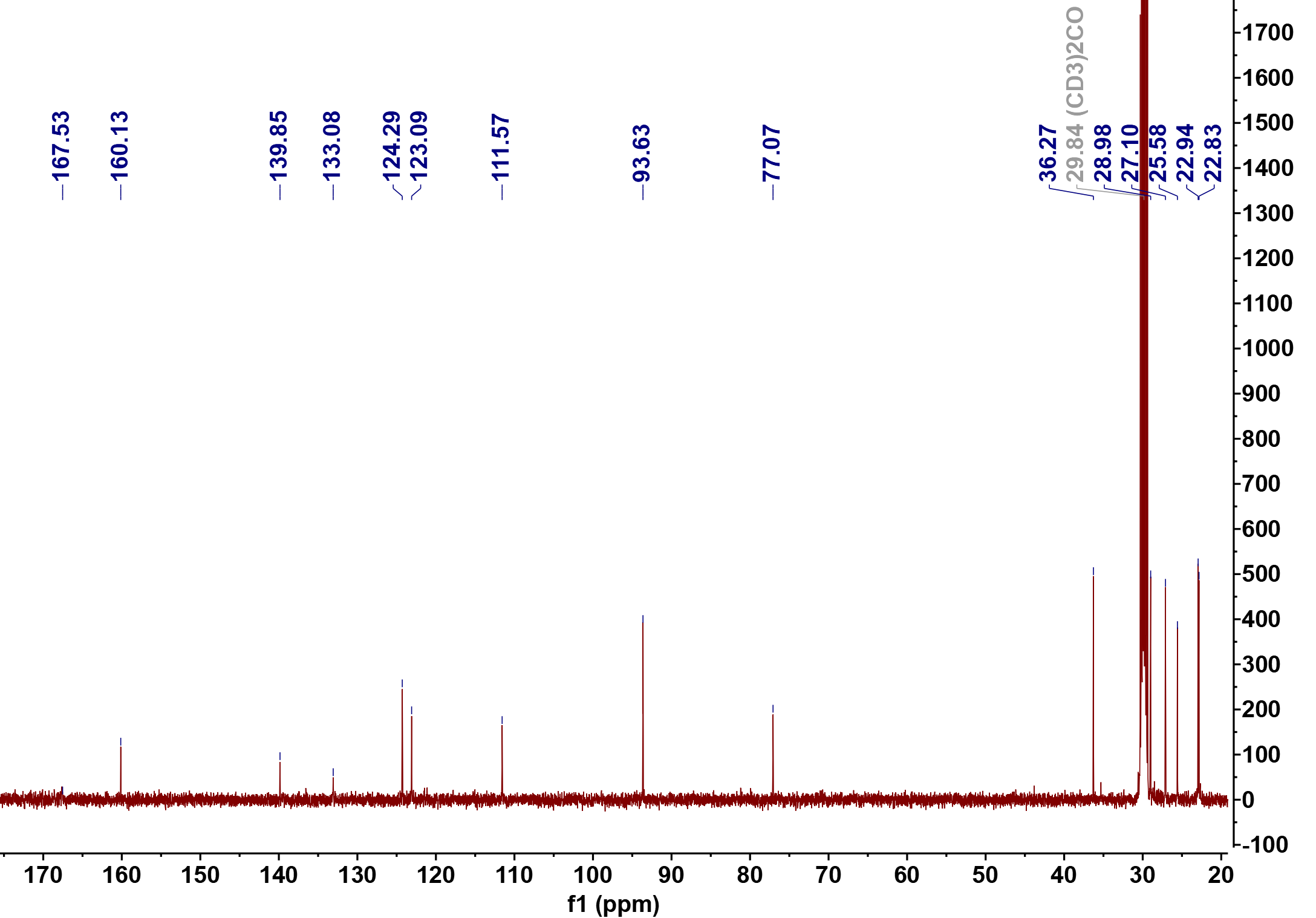


**Figure S6.** ^13^C NMR spectrum of aspersydonol A (**1**; 125 MHz, acetone-*d*_6_).


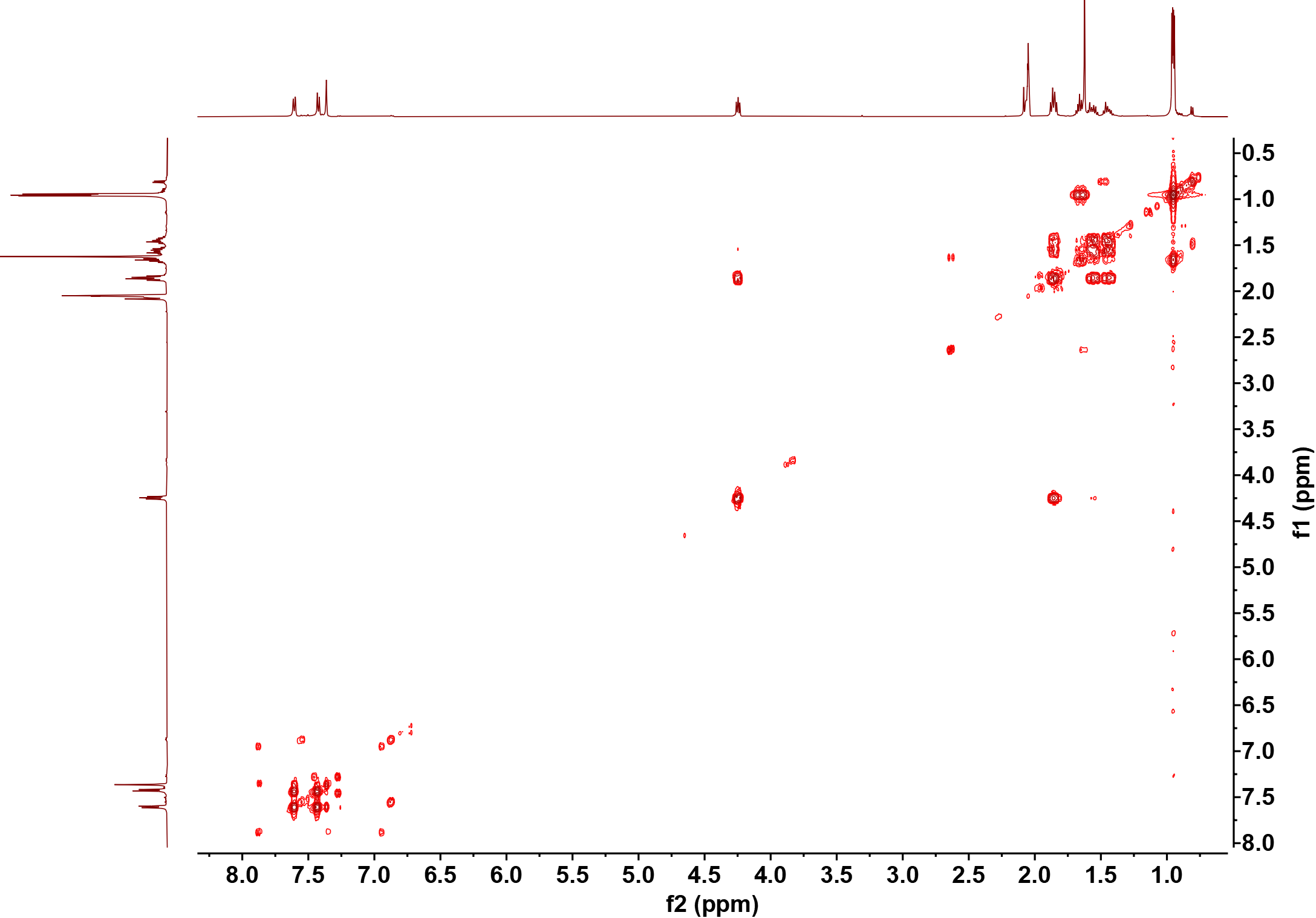


**Figure S7.** ^1^H-^1^H COSY spectrum of aspersydonol A (**1**; 500 MHz, acetone-*d*_6_).


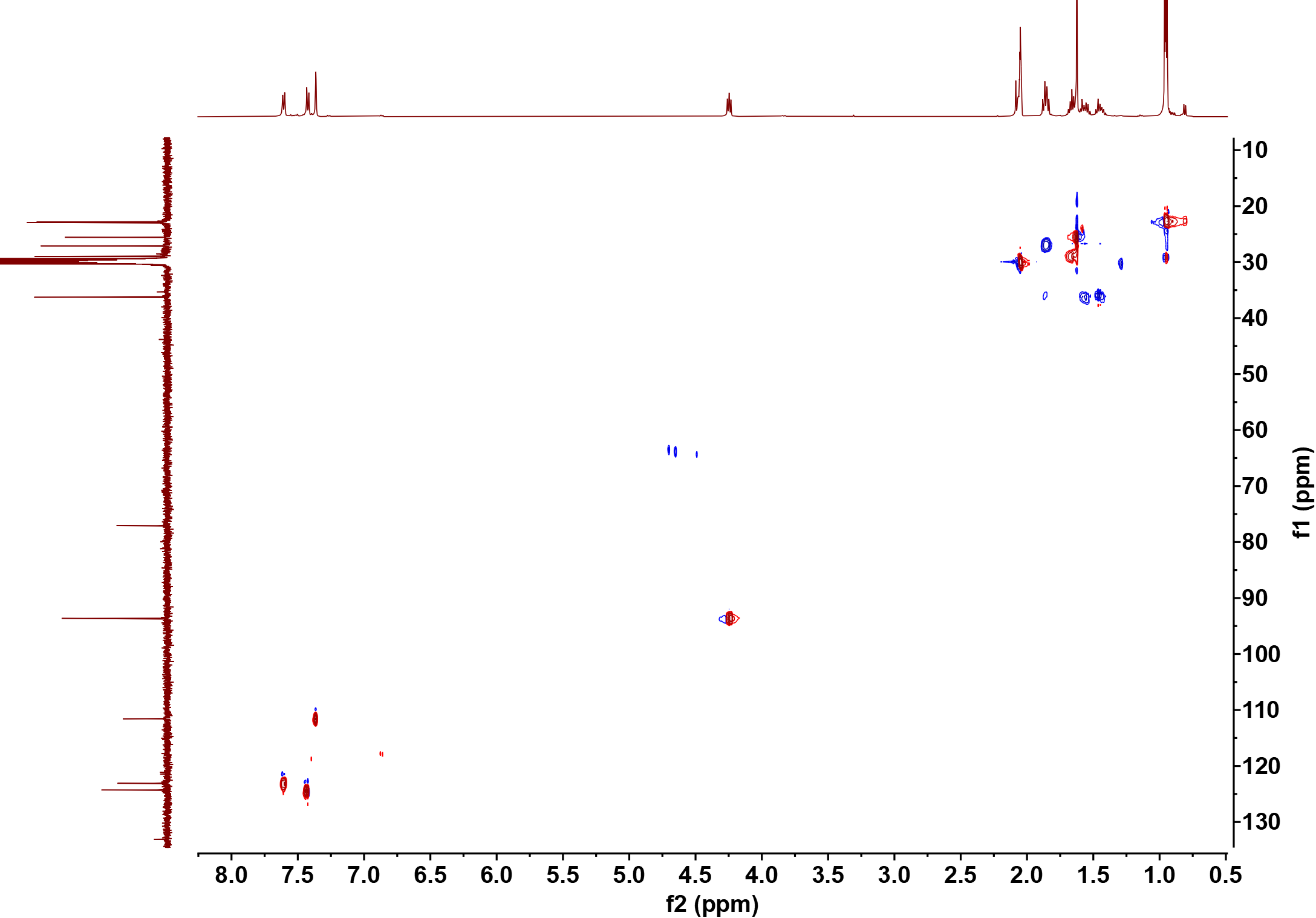


**Figure S8.** HSQC spectrum of aspersydonol A (**1**; 500 MHz, acetone-*d*_6_).


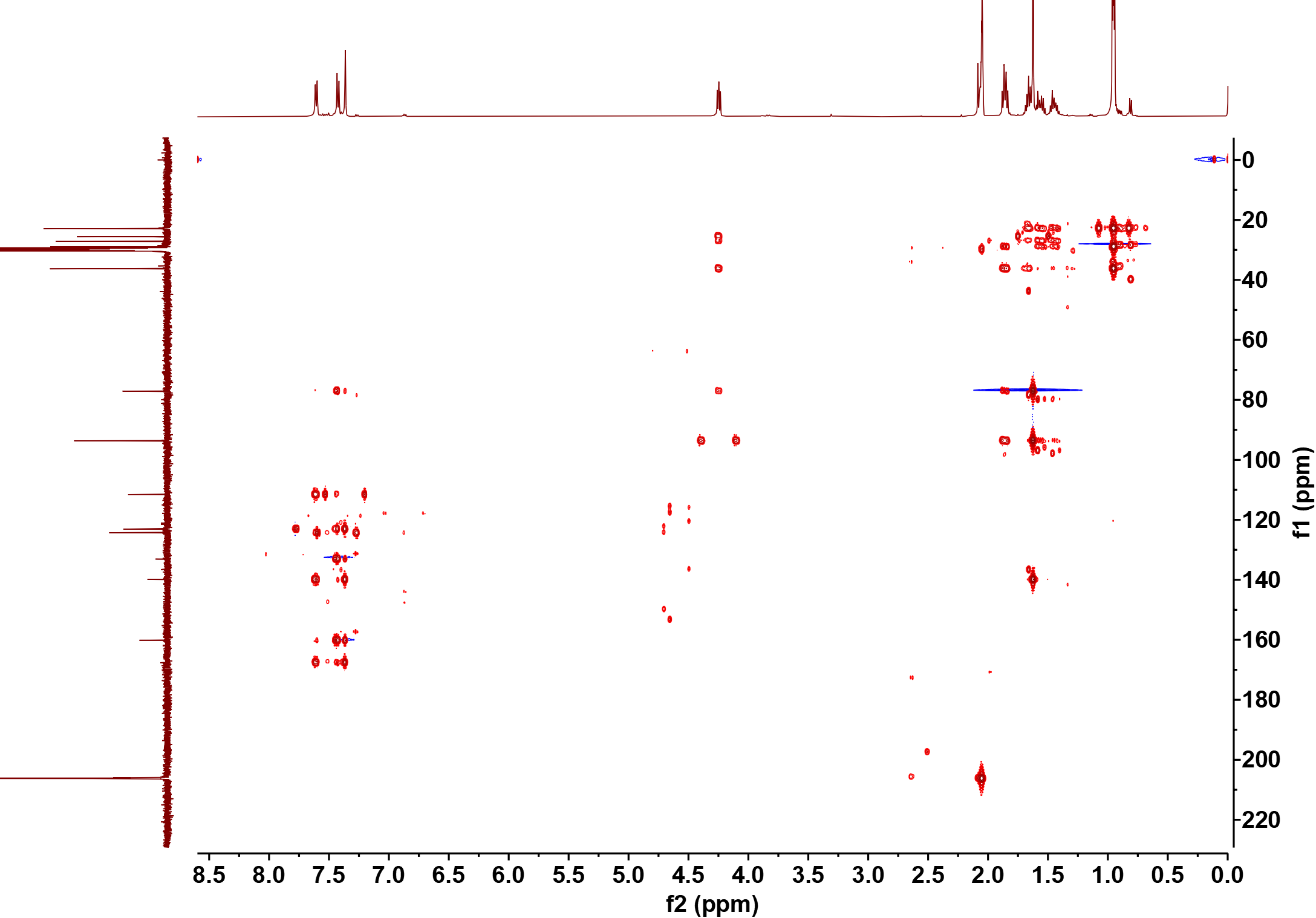


**Figure S9.** HMBC spectrum of aspersydonol A (**1**; 500 MHz, acetone-*d*_6_).


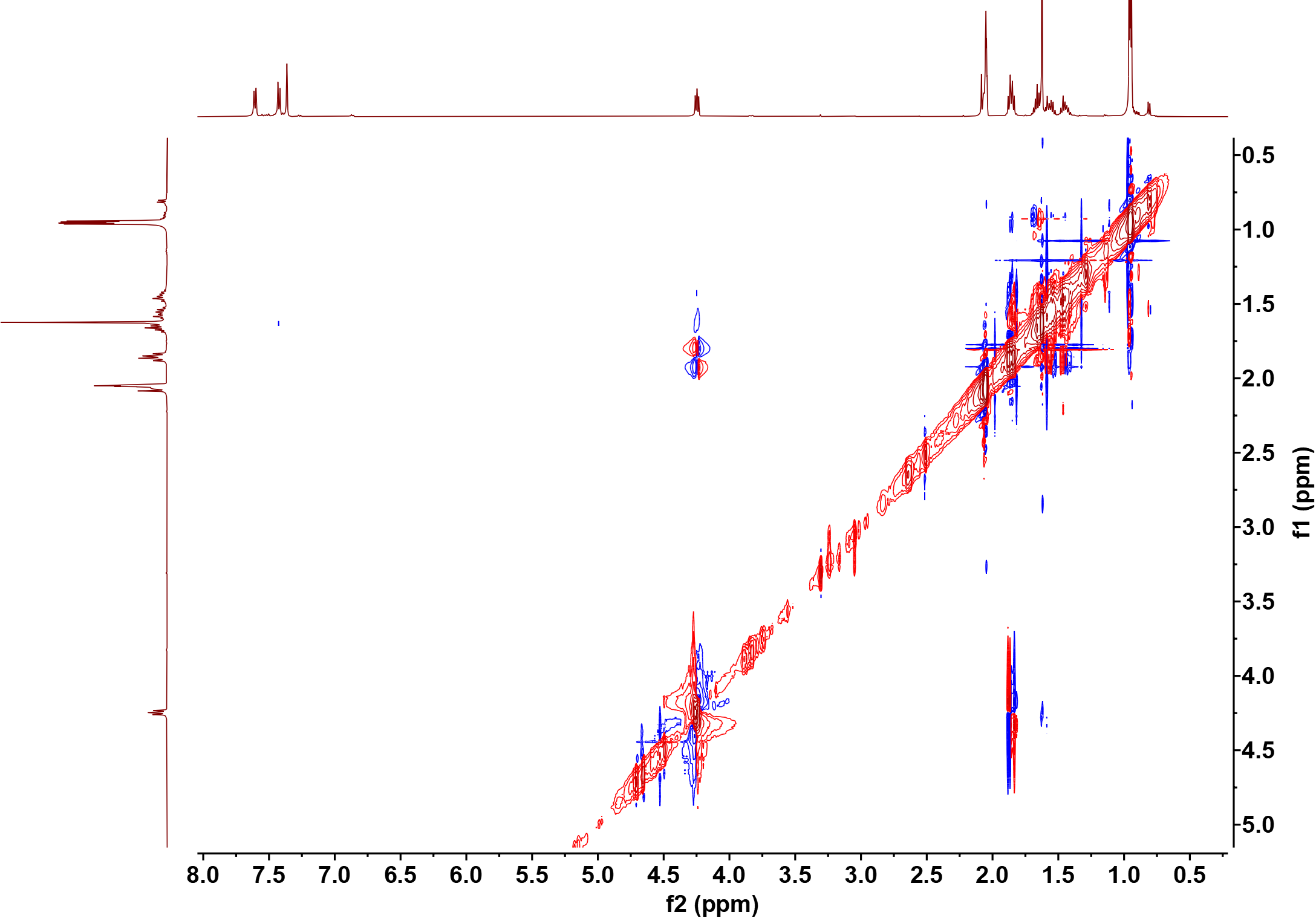


**Figure S10.** ROESY spectrum of aspersydonol A (**1**; 500 MHz, acetone-*d*_6_).


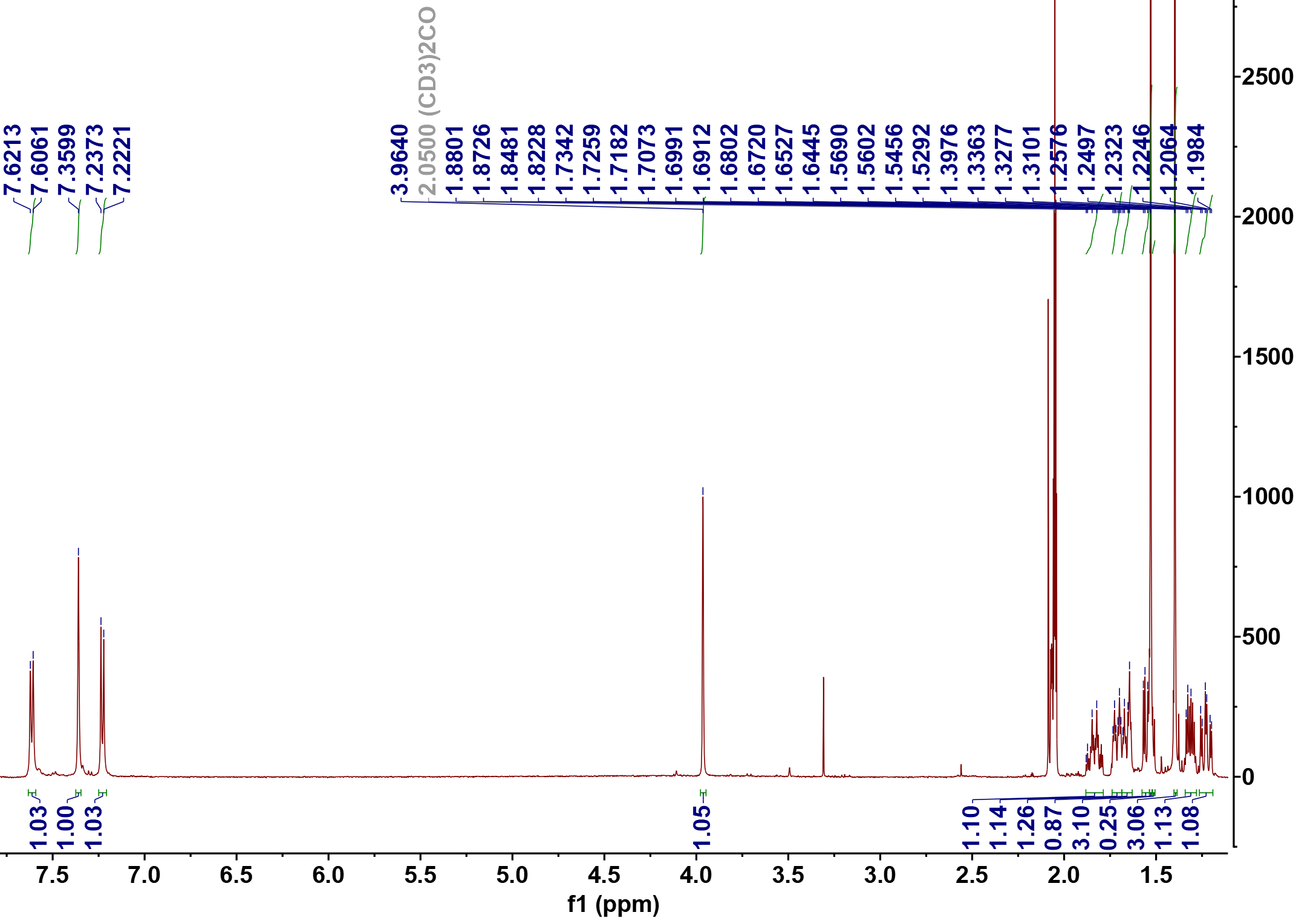


**Figure S11.** ^1^H NMR spectrum of aspersydonol B (**2**; 500 MHz, acetone-*d*_6_).


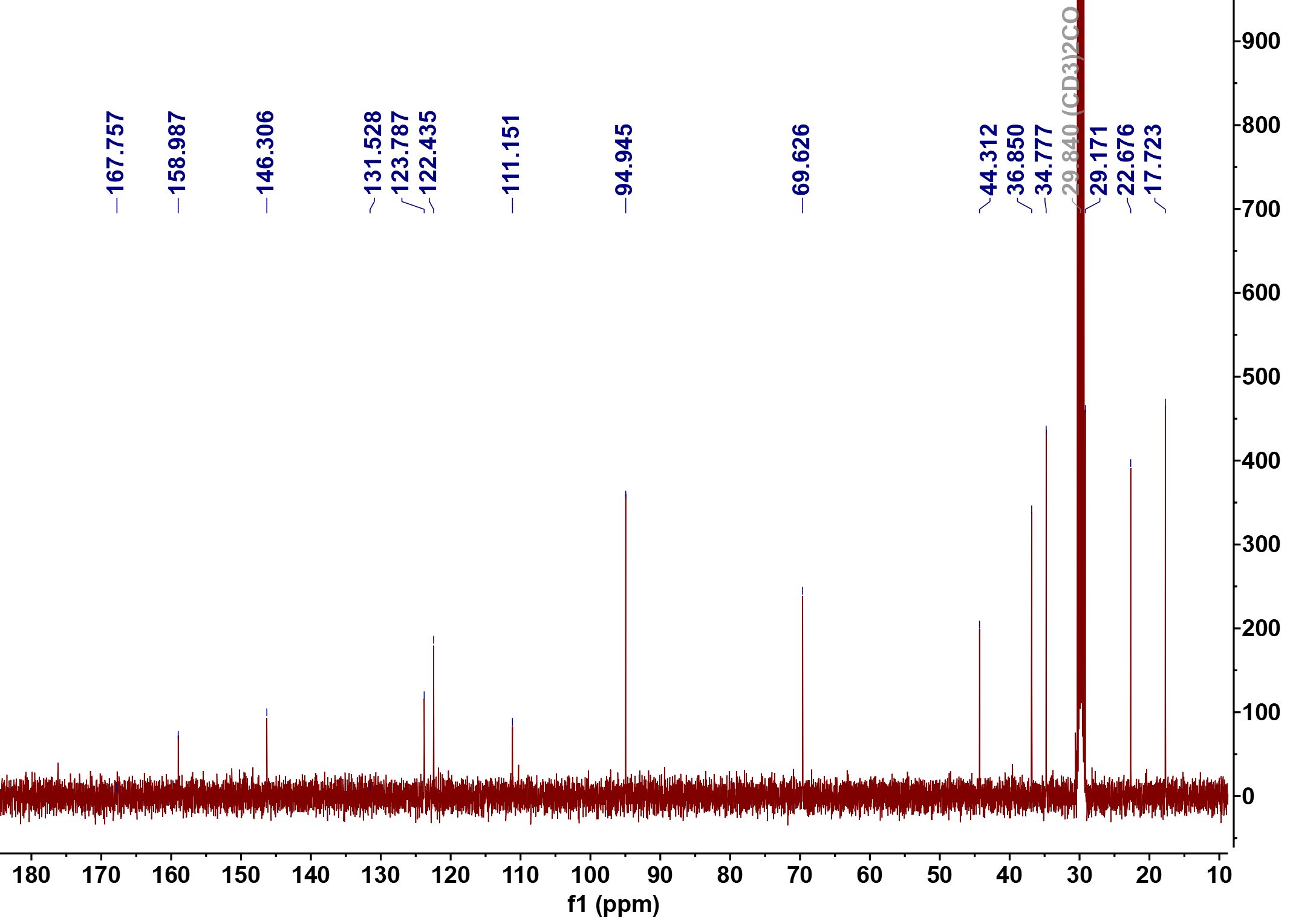


**Figure S12.** ^13^C NMR spectrum of aspersydonol B (**2**; 125 MHz, acetone-*d*_6_).


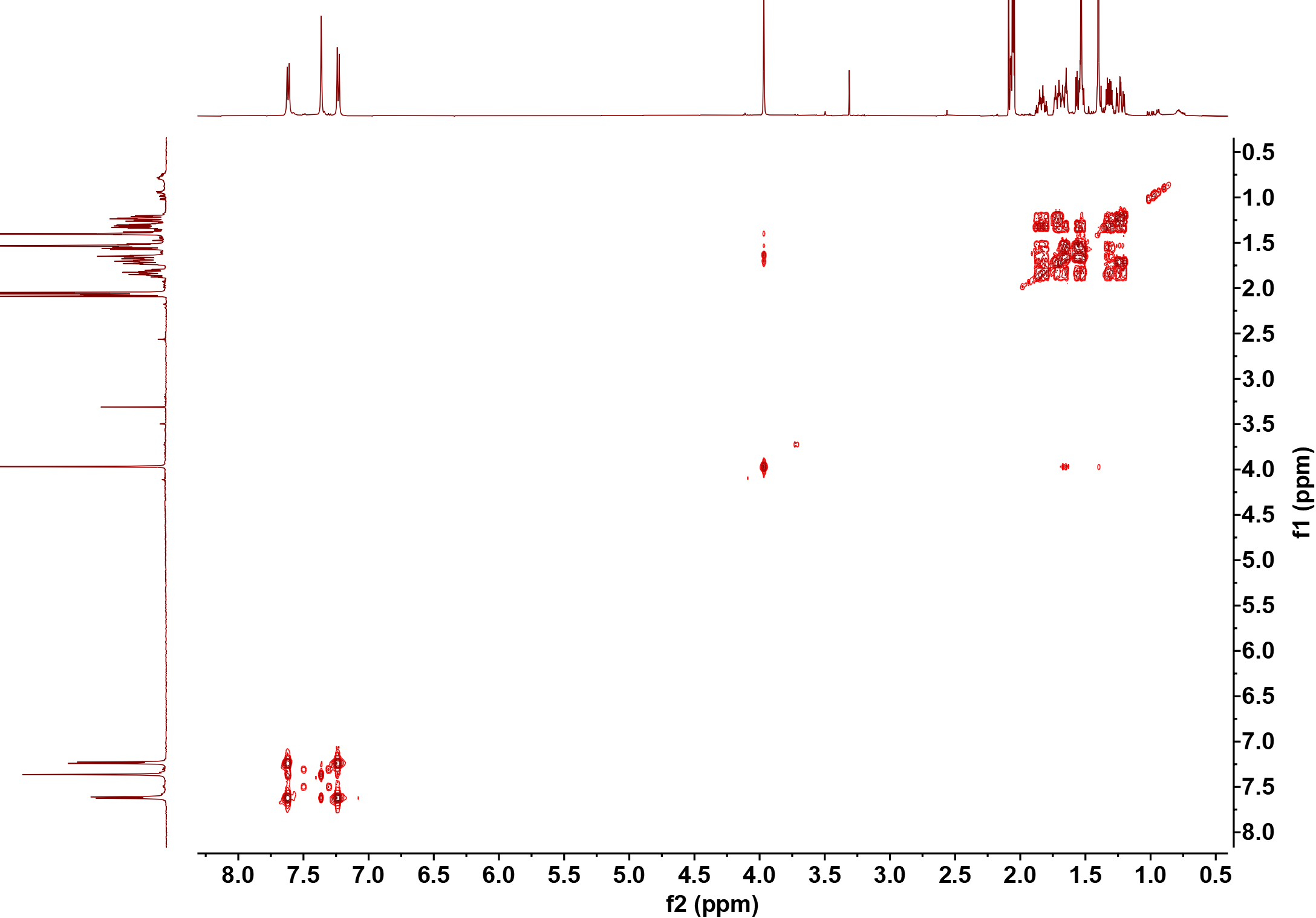


**Figure S13.** ^1^H-^1^H COSY spectrum of aspersydonol B (**2**; 500 MHz, acetone-*d*_6_).


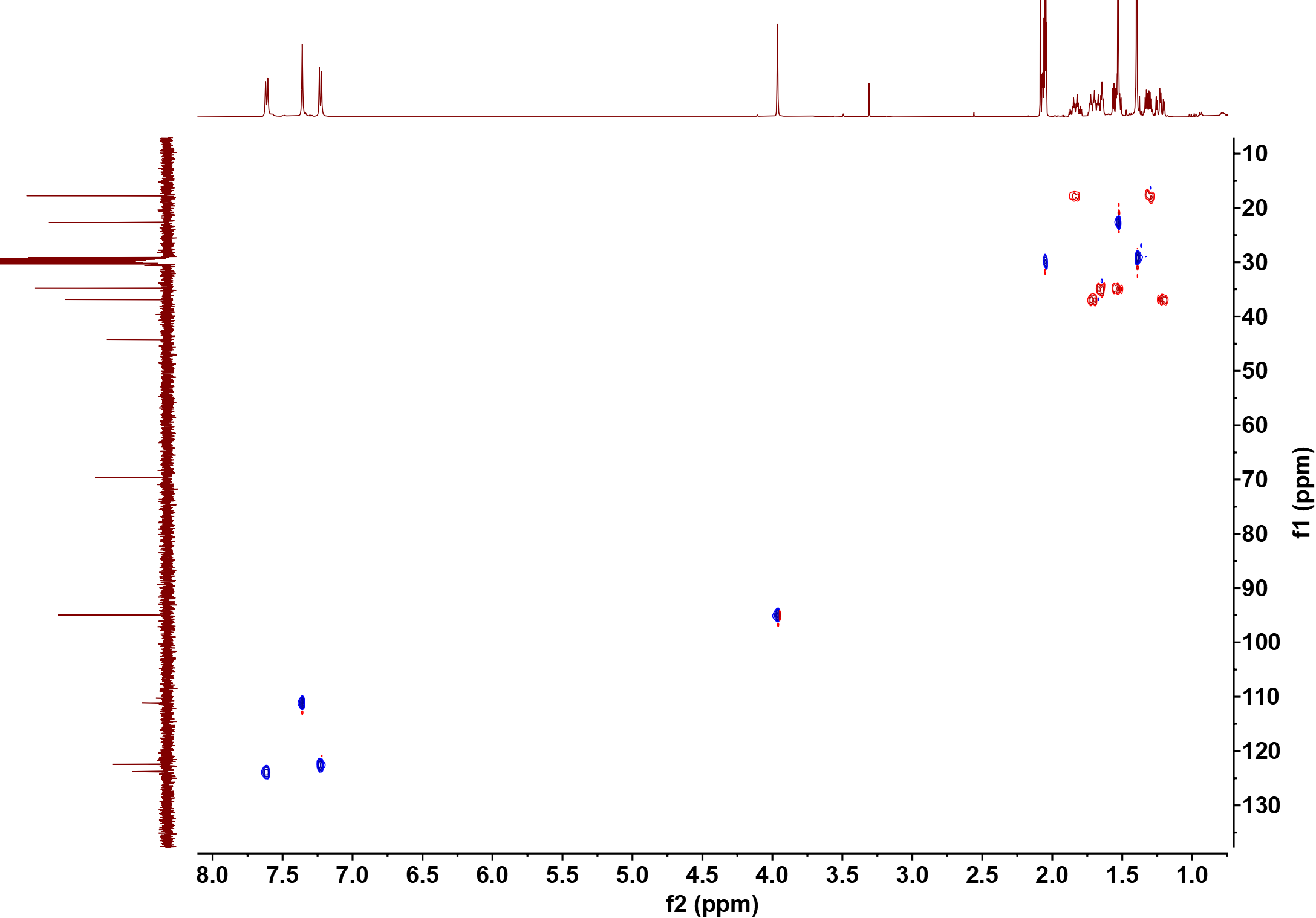


**Figure S14.** HSQC spectrum of aspersydonol B (**2**; 500 MHz, acetone-*d*_6_).


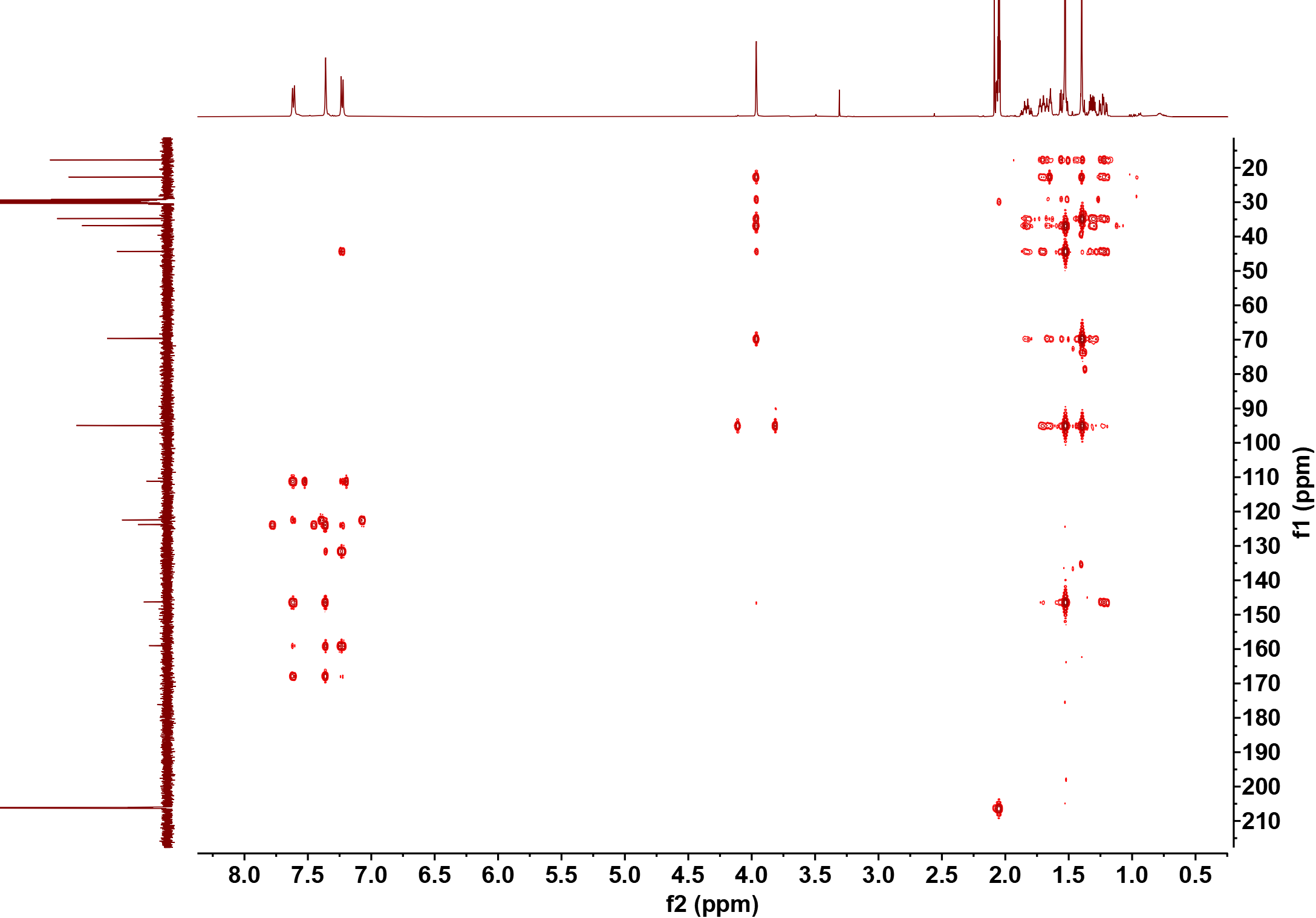


**Figure S15.** HMBC spectrum of aspersydonol B (**2**; 500 MHz, acetone-*d*_6_).


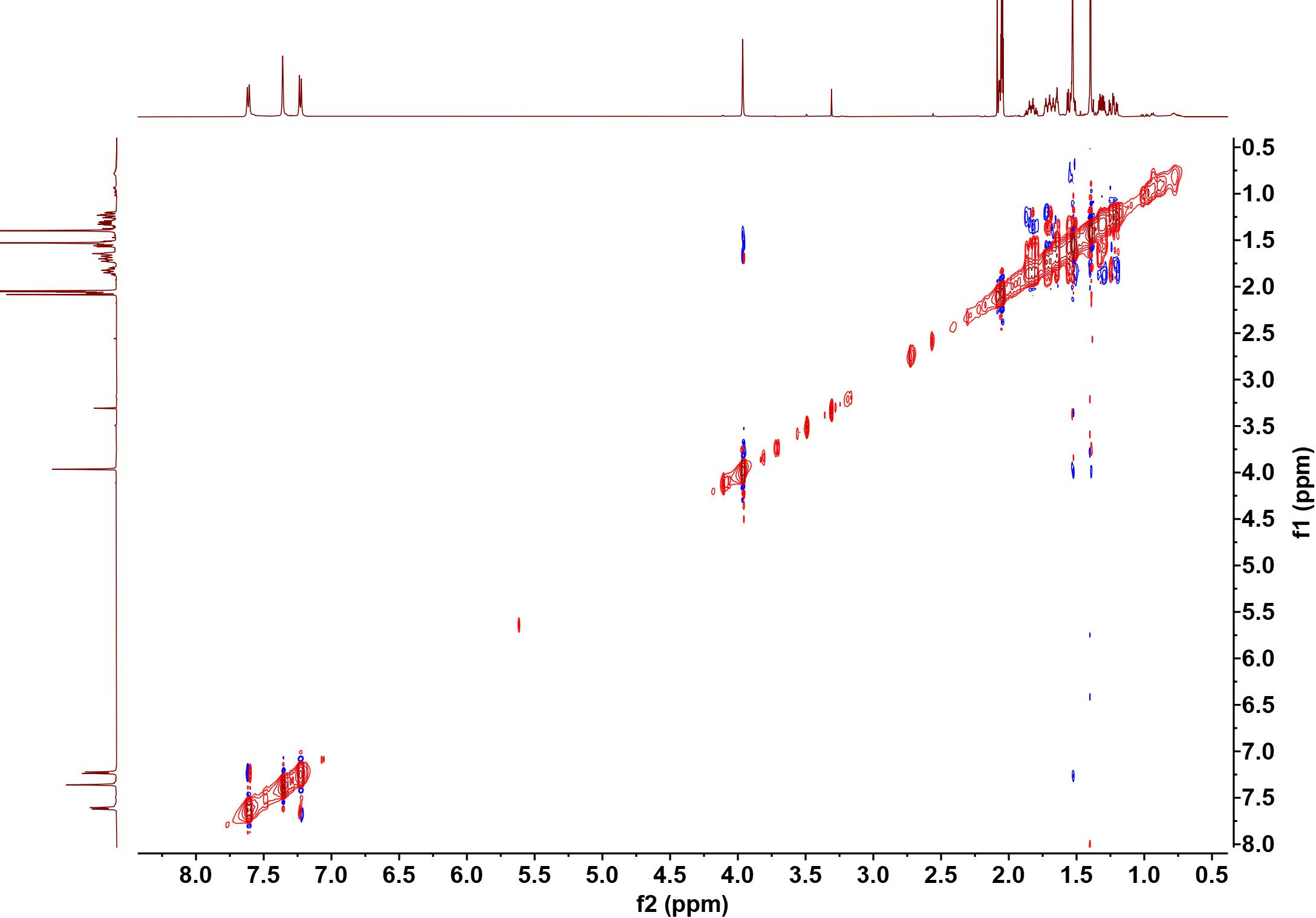


**Figure S16.** ROESY spectrum of aspersydonol B (**2**; 500 MHz, acetone-*d*_6_).

|  | |
| --- | --- |
| Conformers | Populations (%) |
| 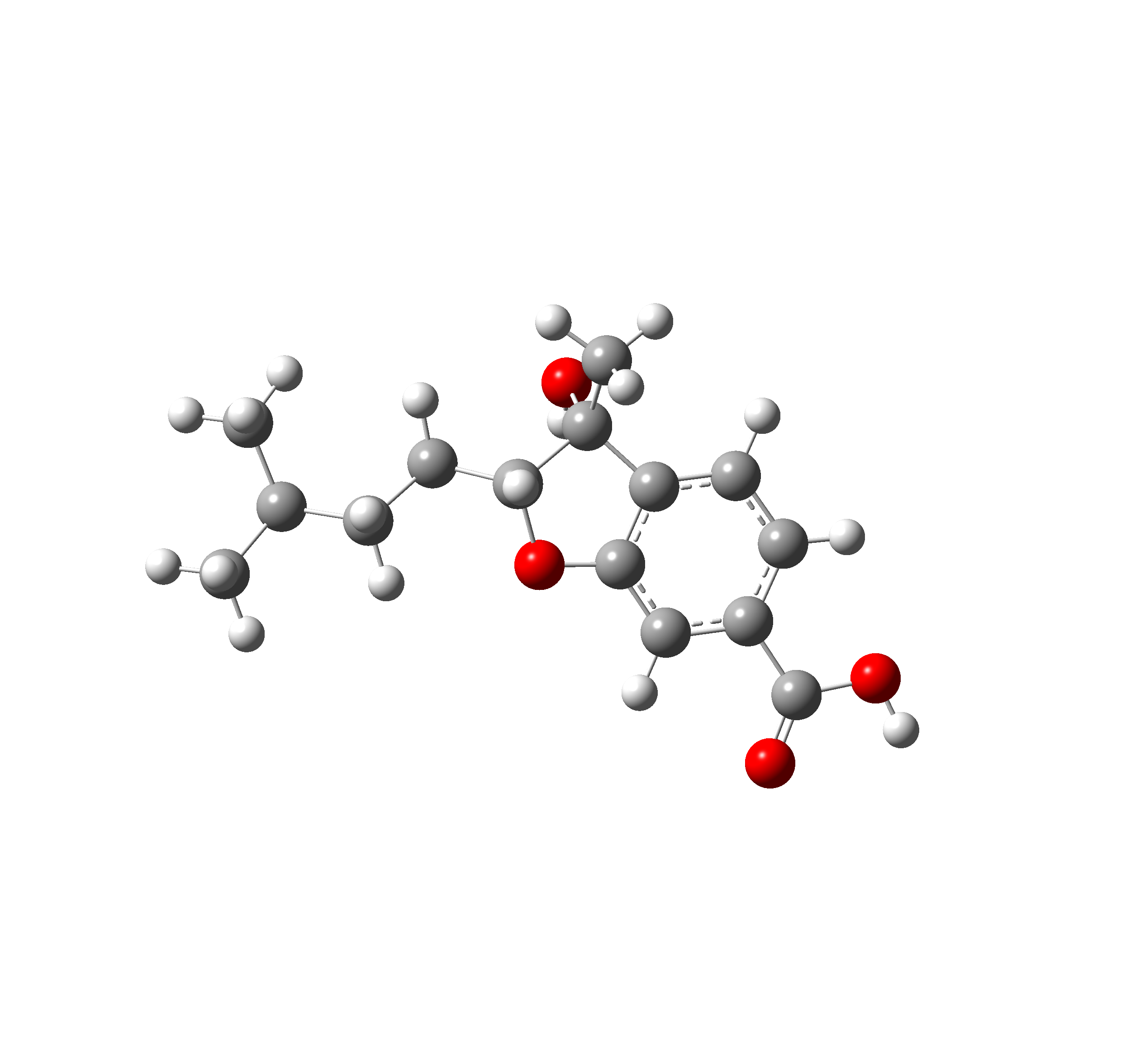 | 7.76 |
| 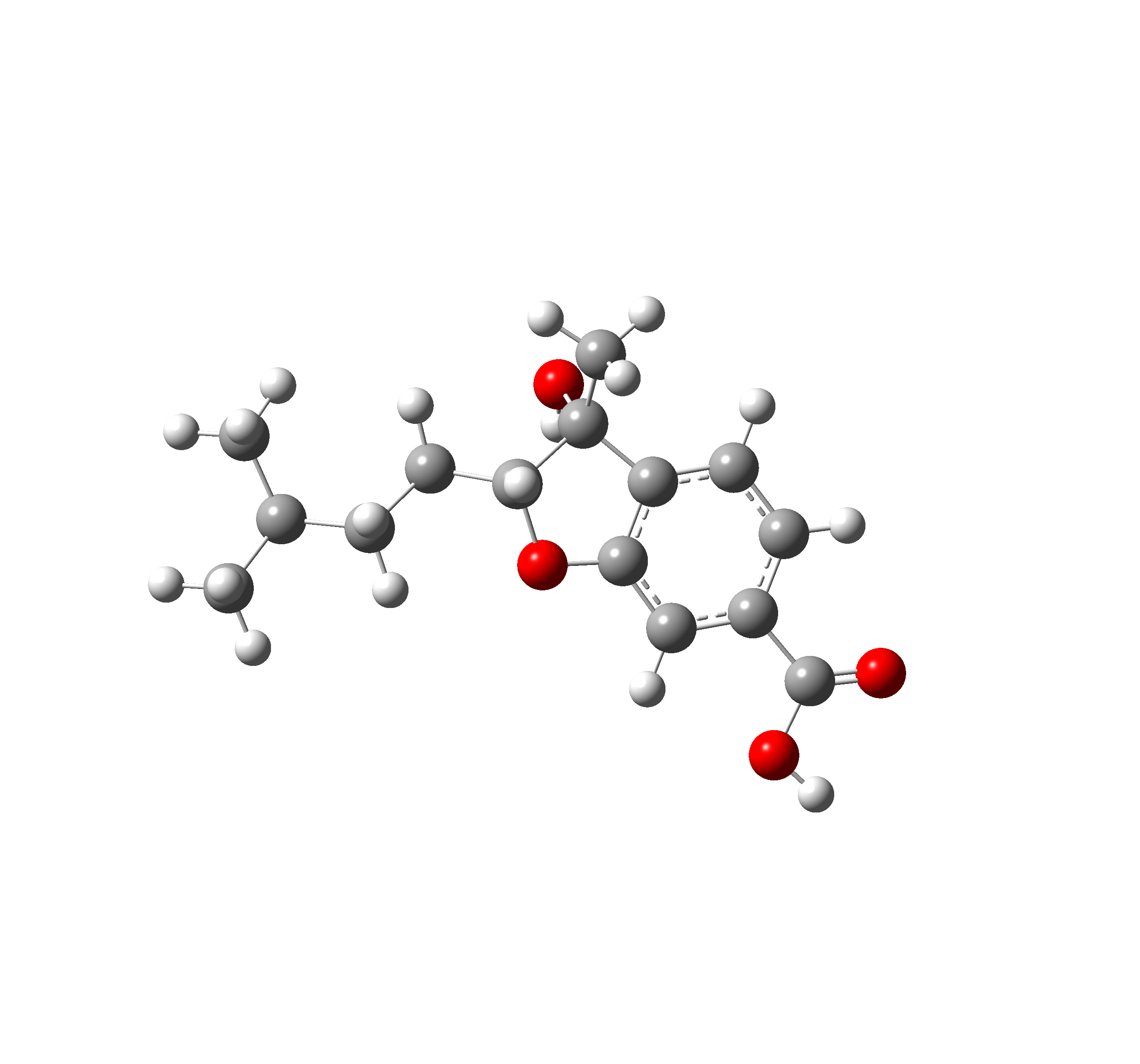 | 8.25 |
| 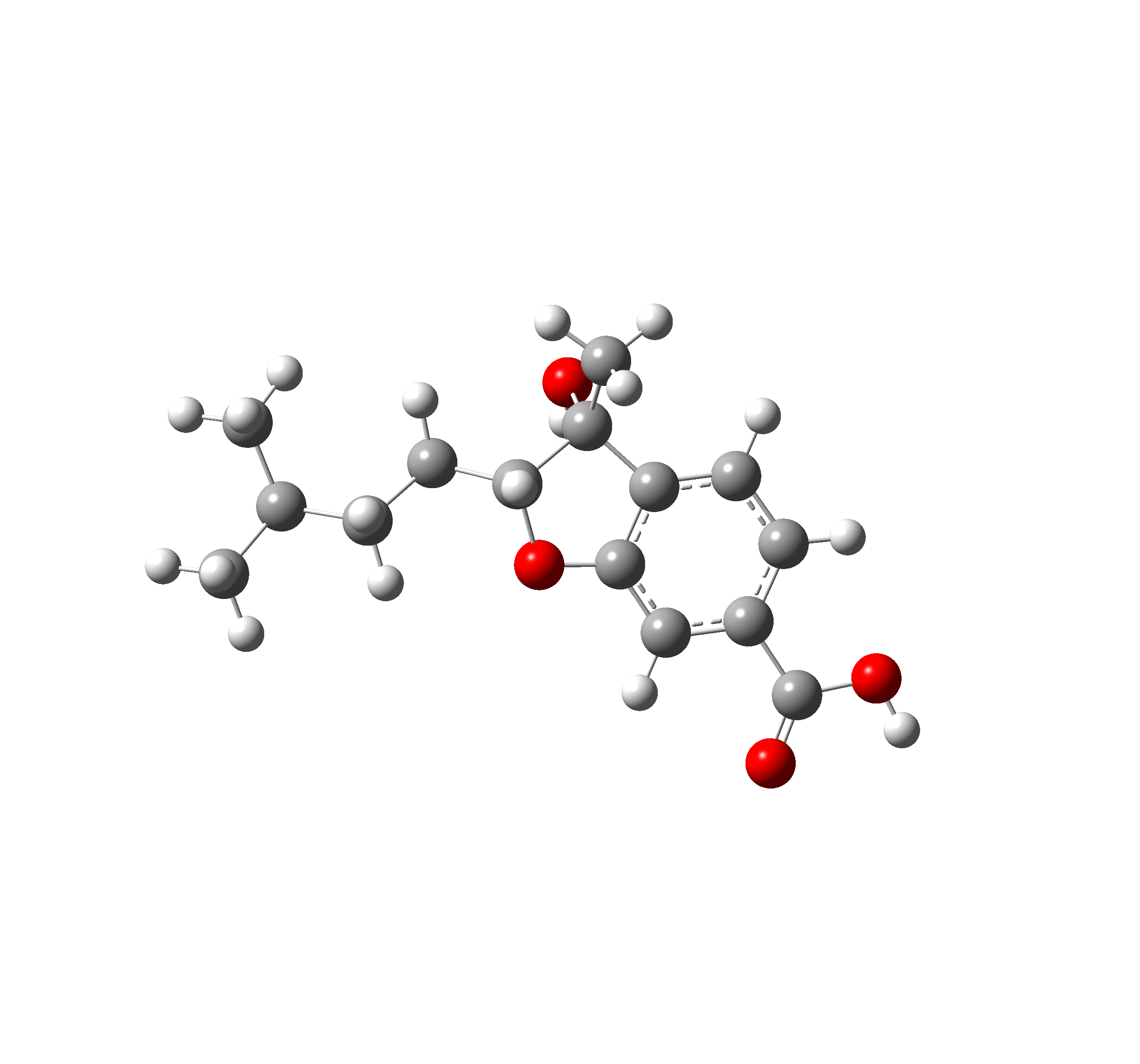 | 7.75 |
| 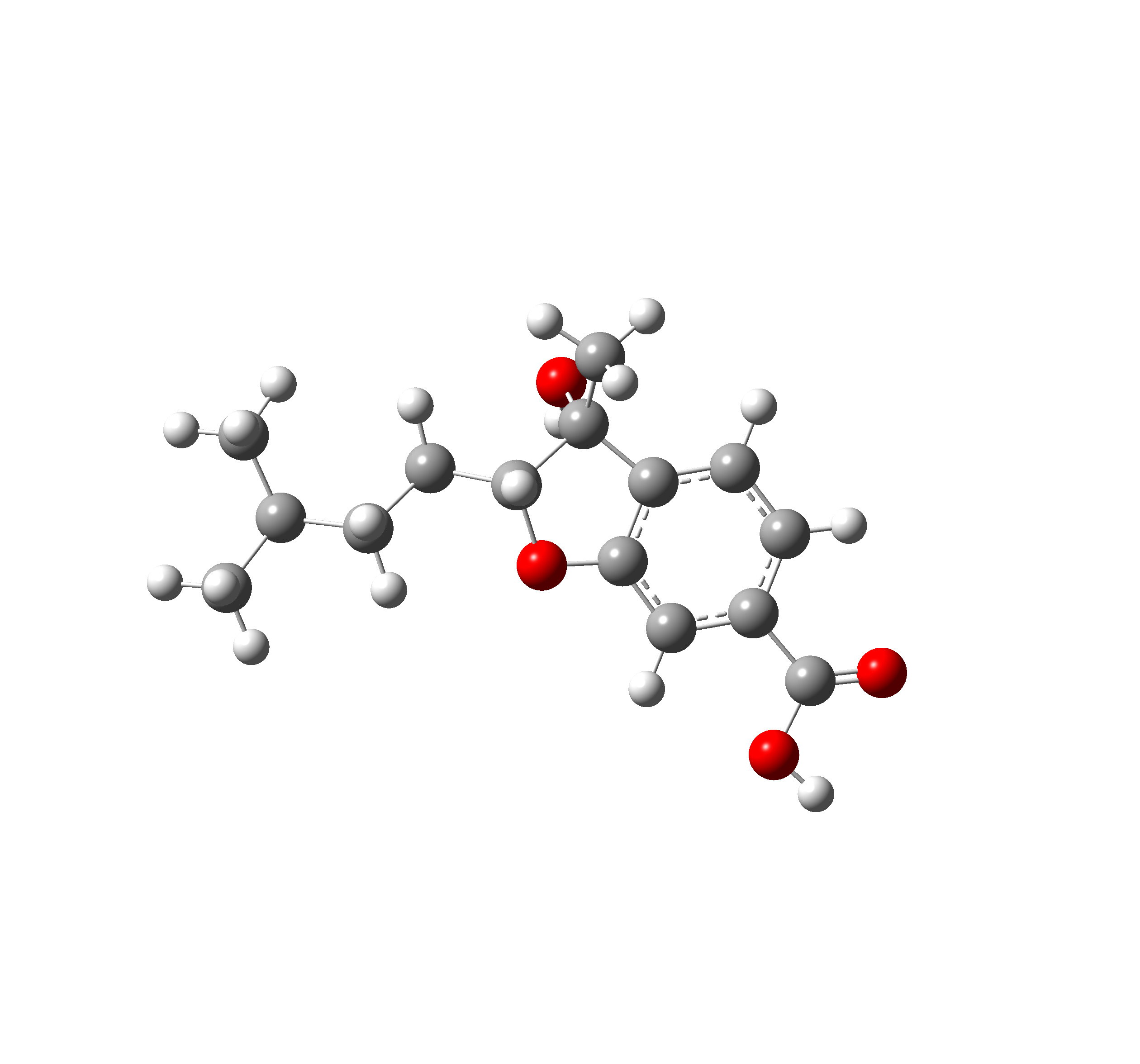 | 8.26 |
| 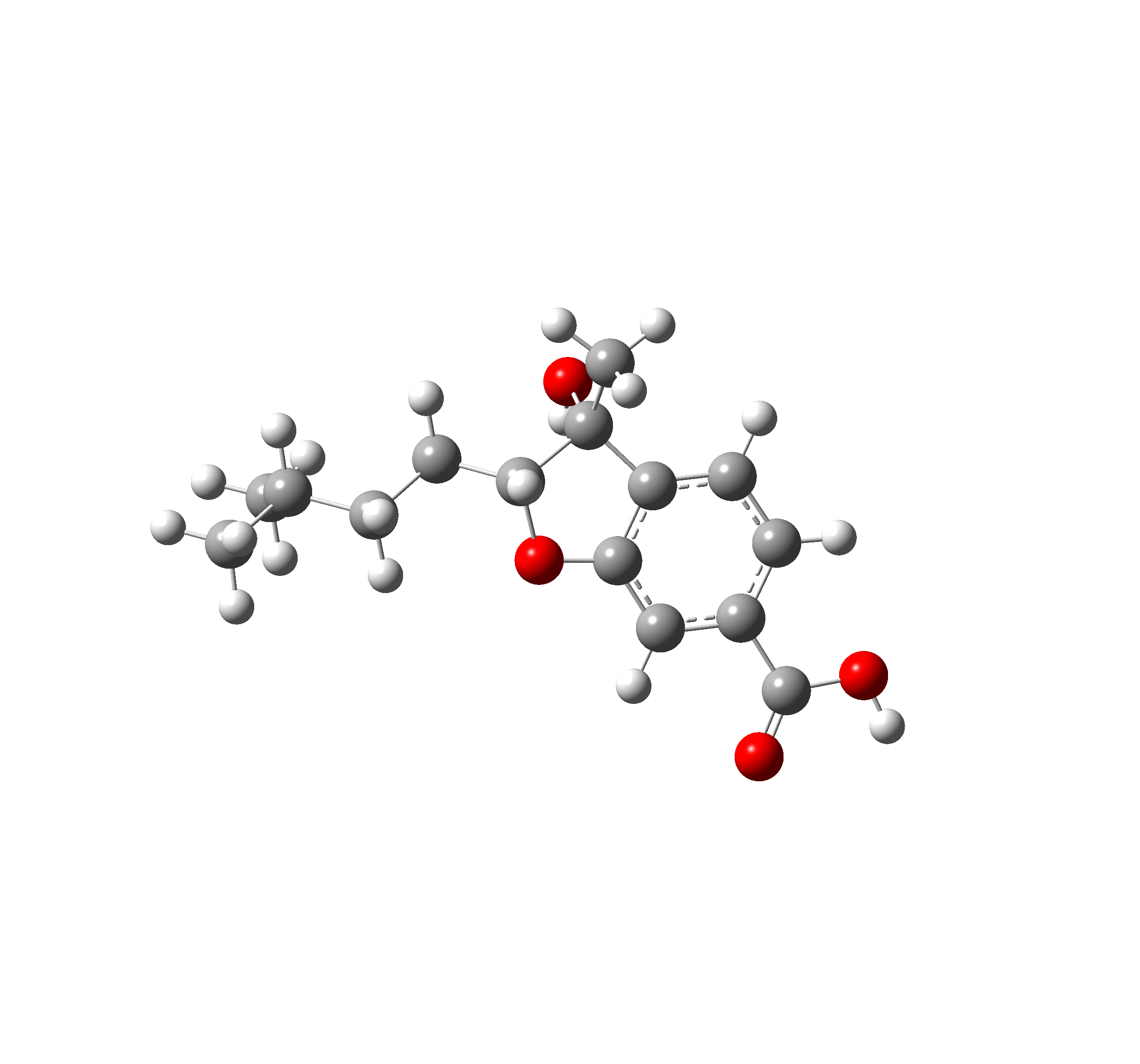 | 7.1 |
| 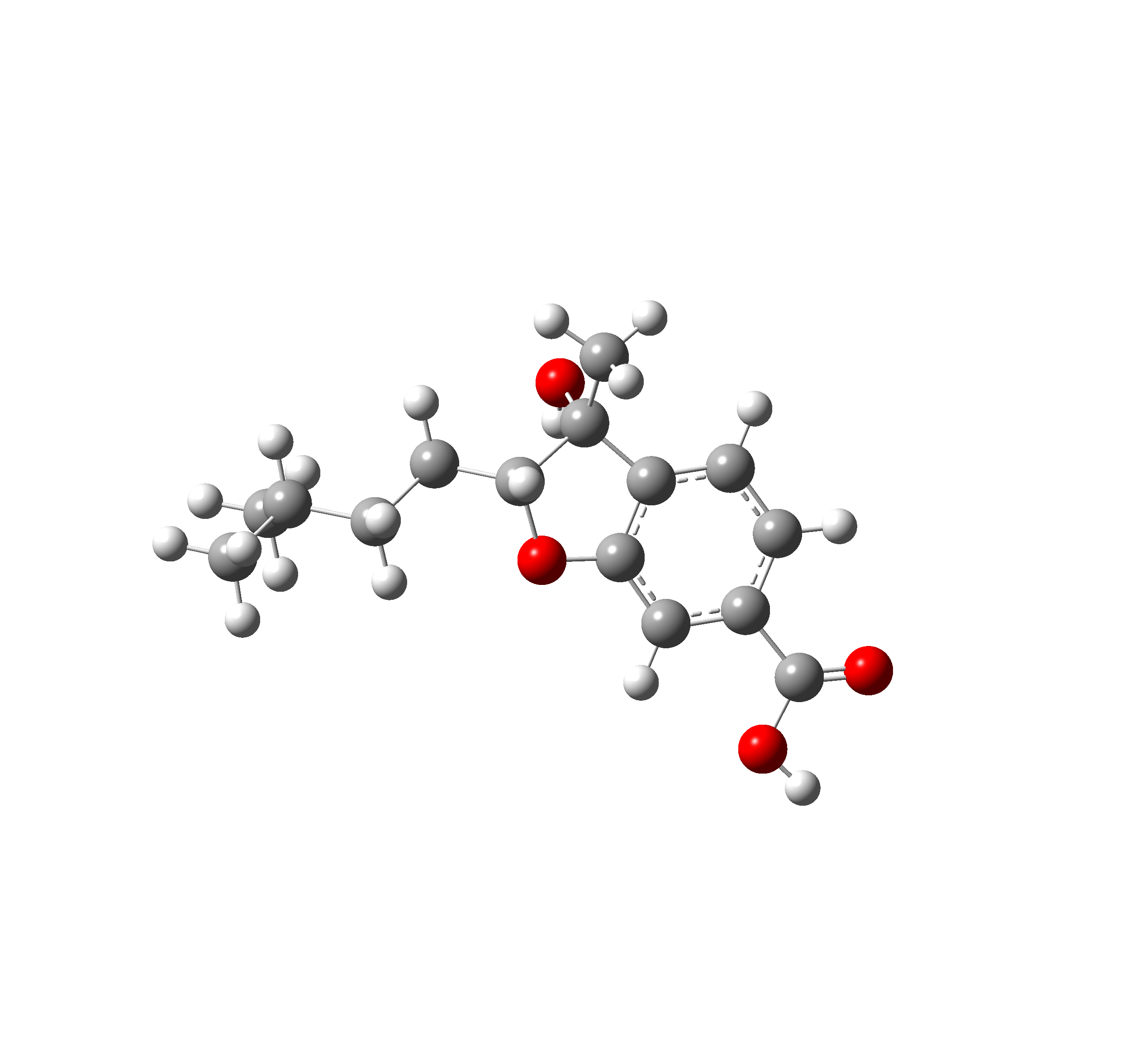 | 7.57 |
| 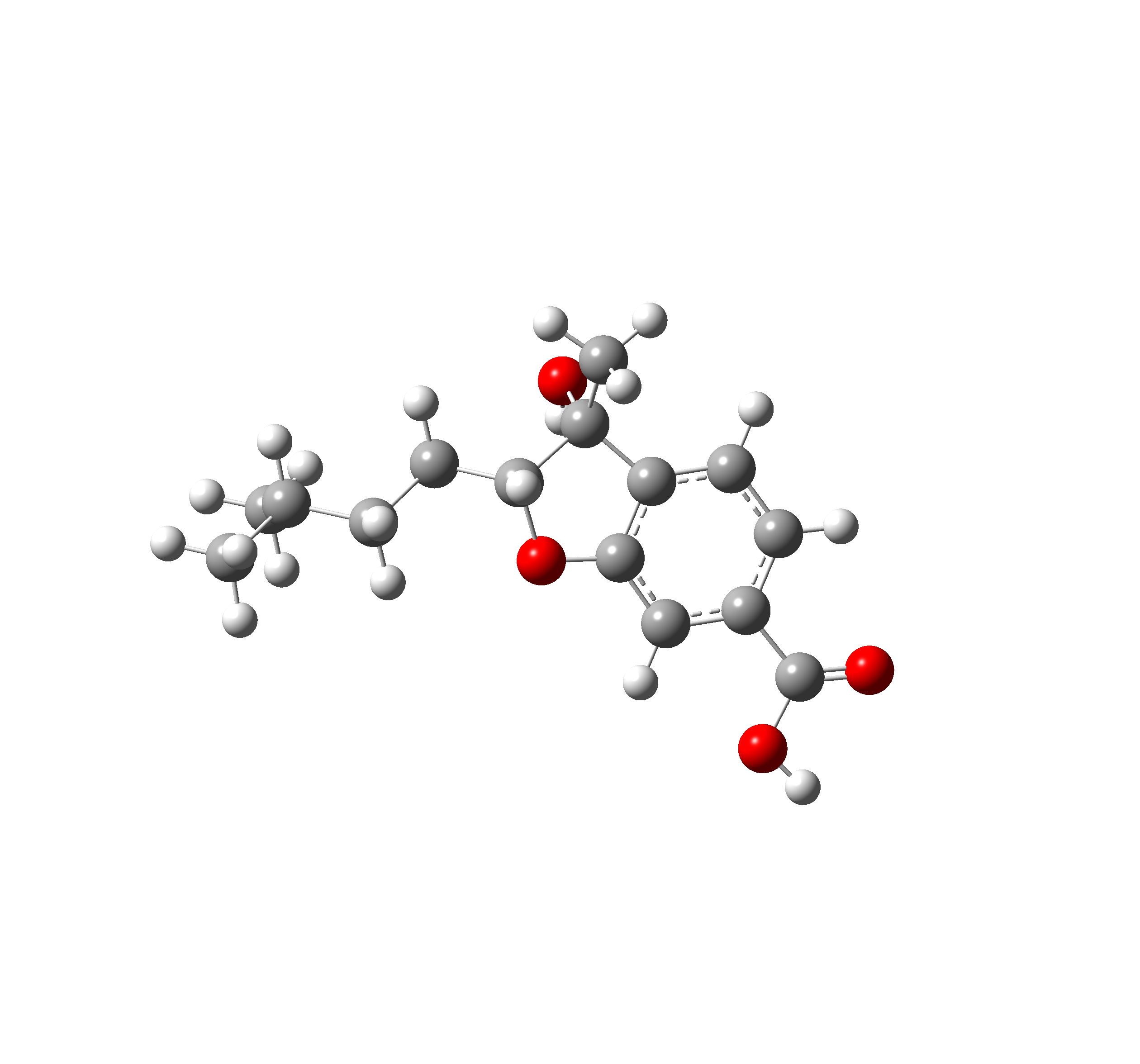 | 7.58 |
| 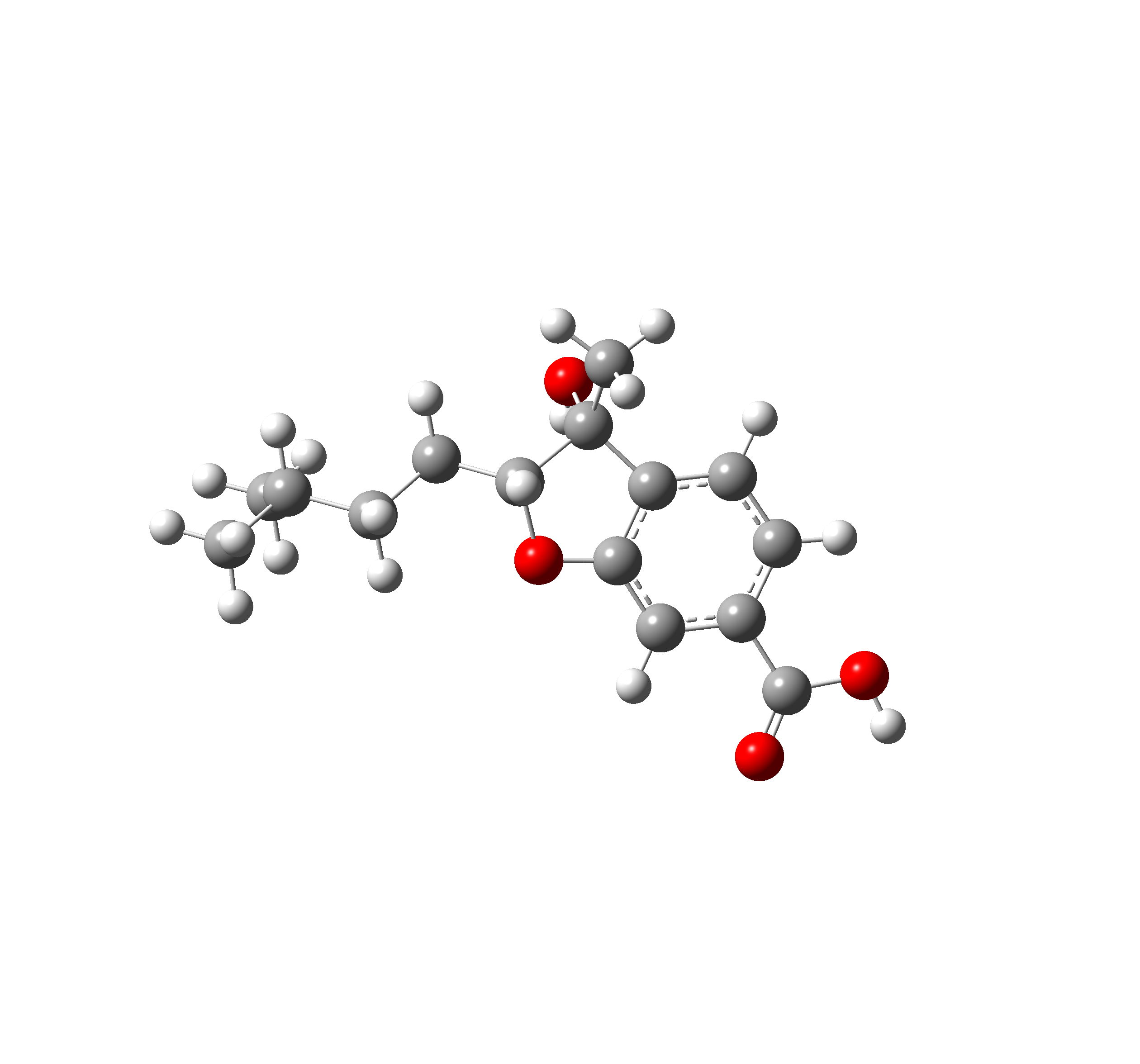 | 7.11 |
| 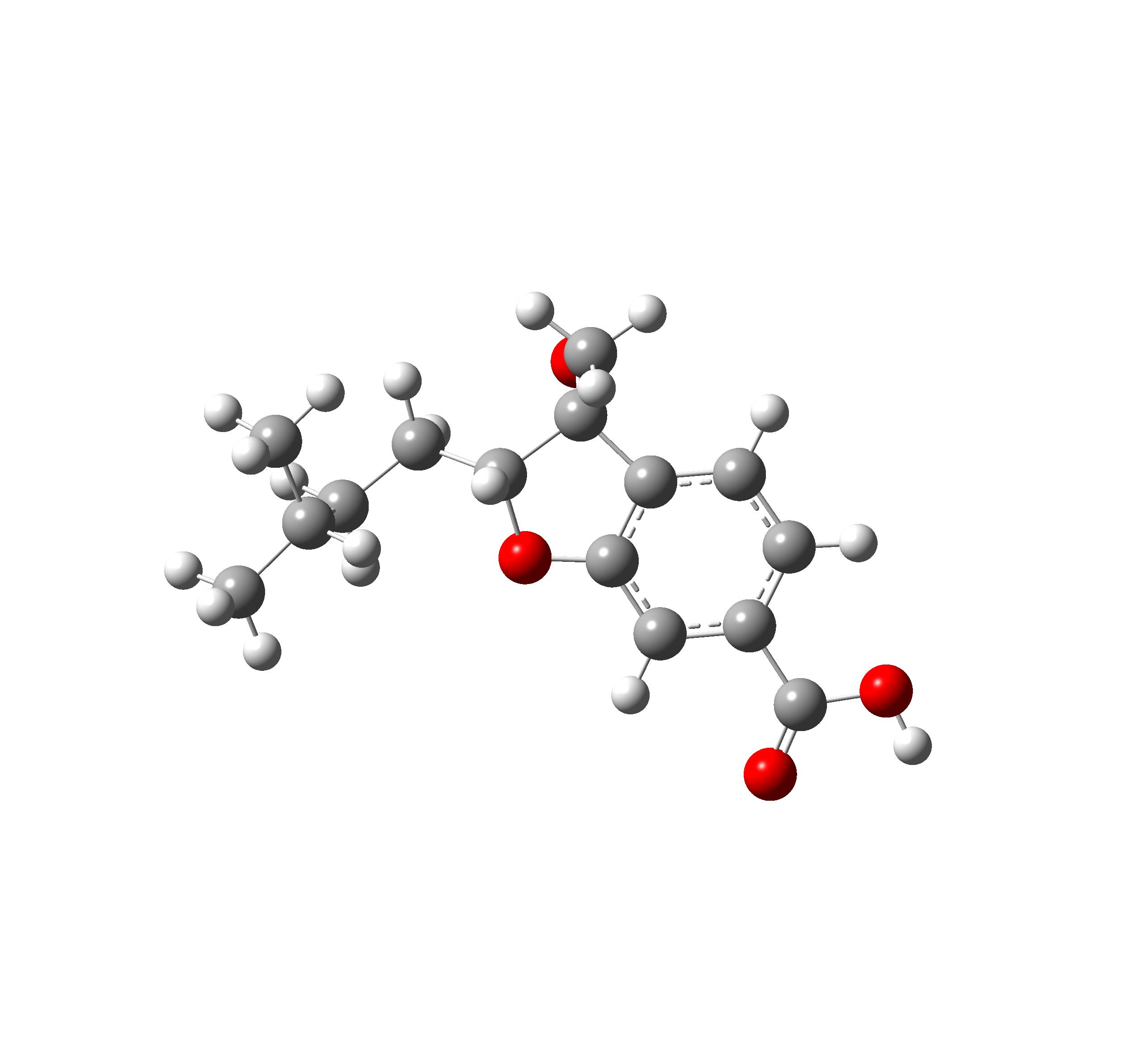 | 1.75 |
| 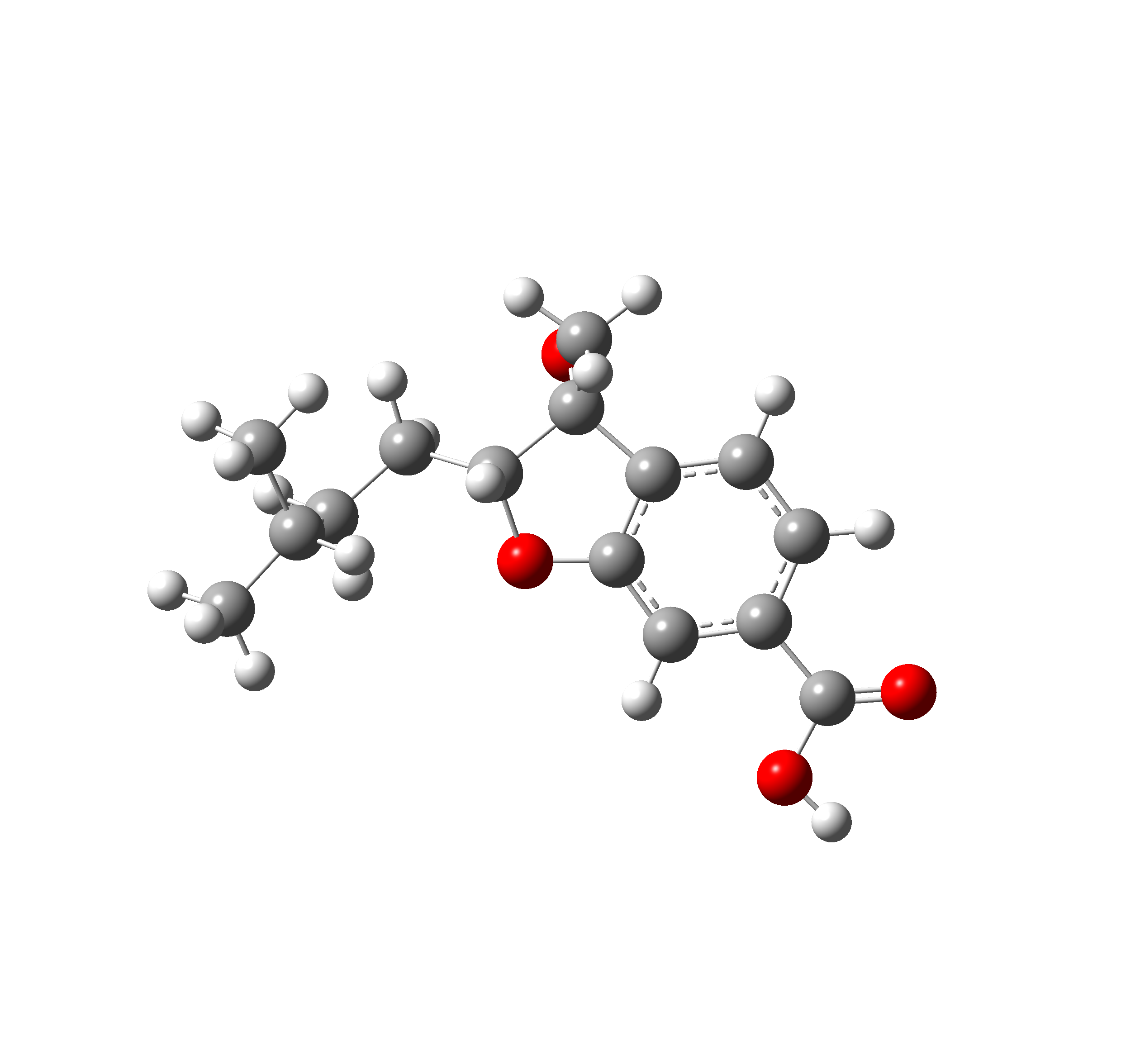 | 1.85 |
| 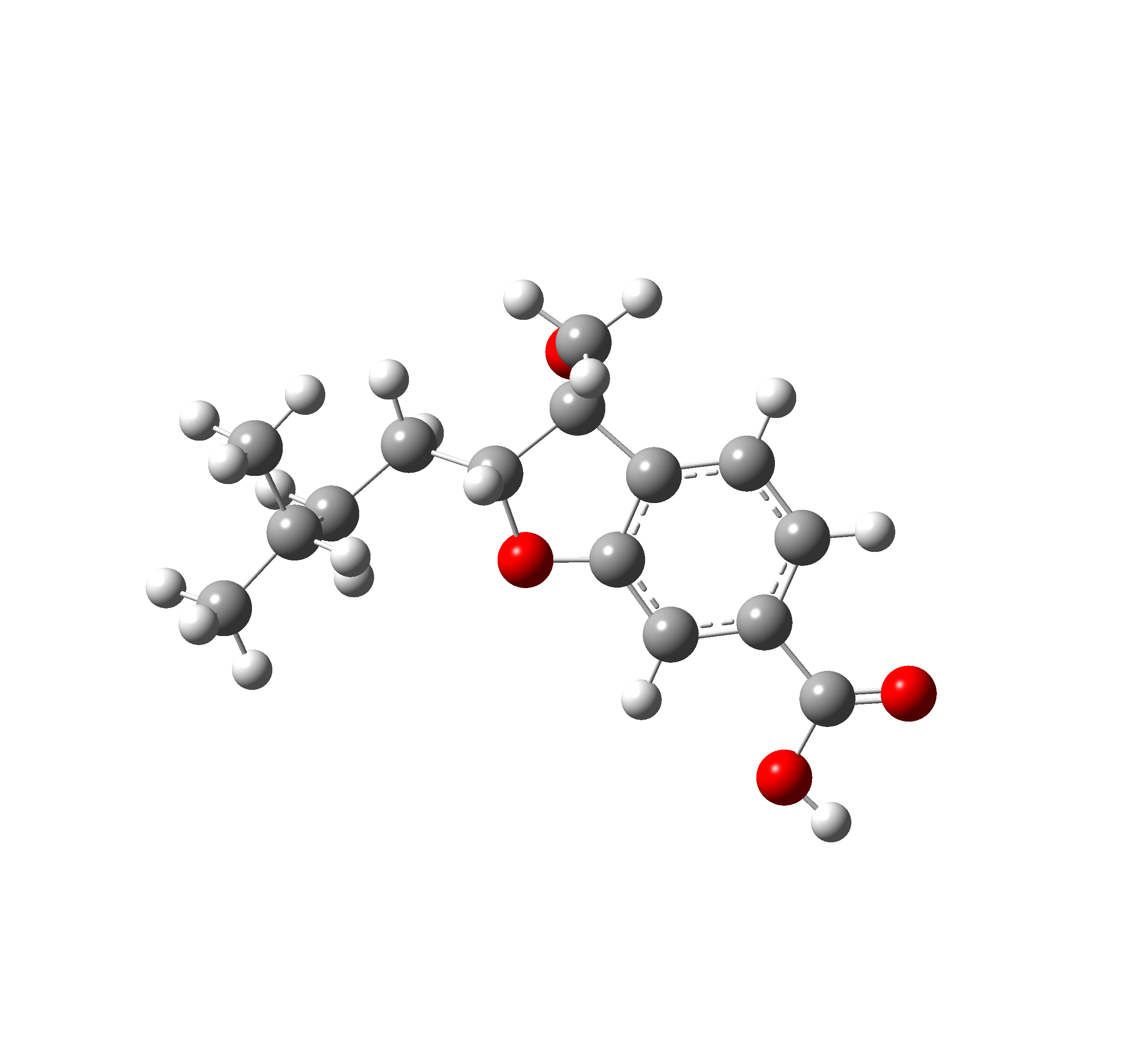 | 1.86 |
| 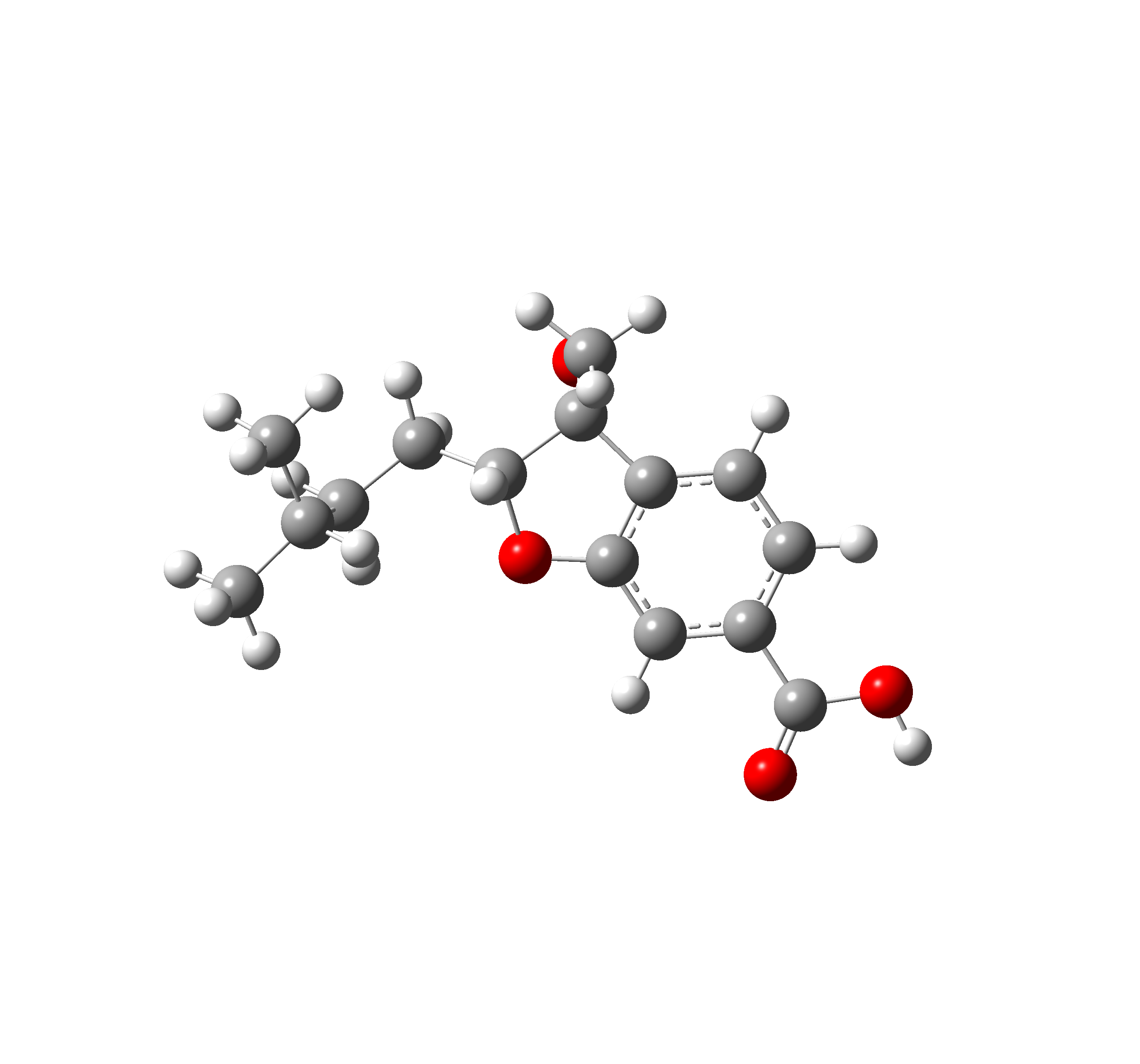 | 1.75 |
| 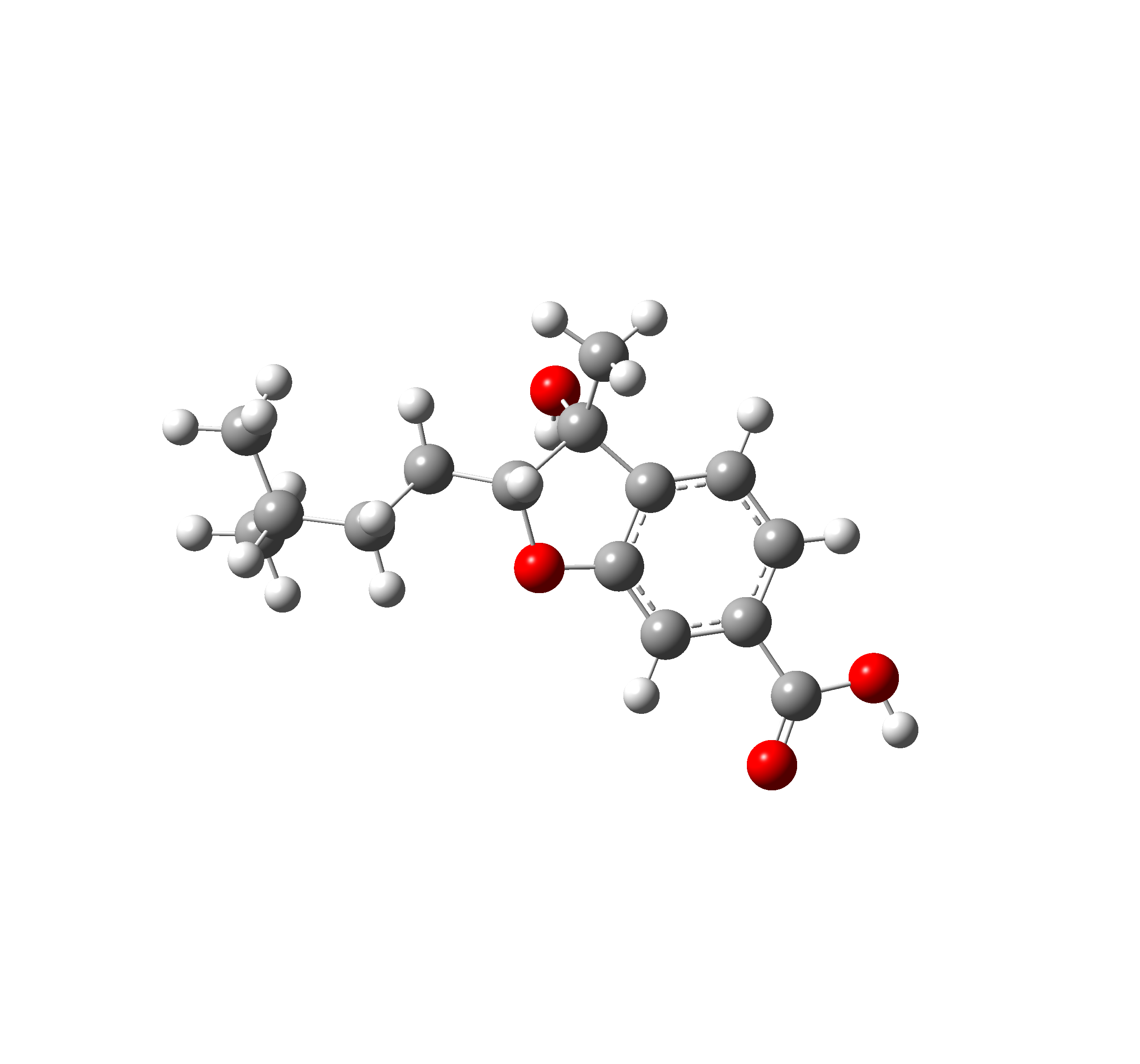 | 1.67 |
| 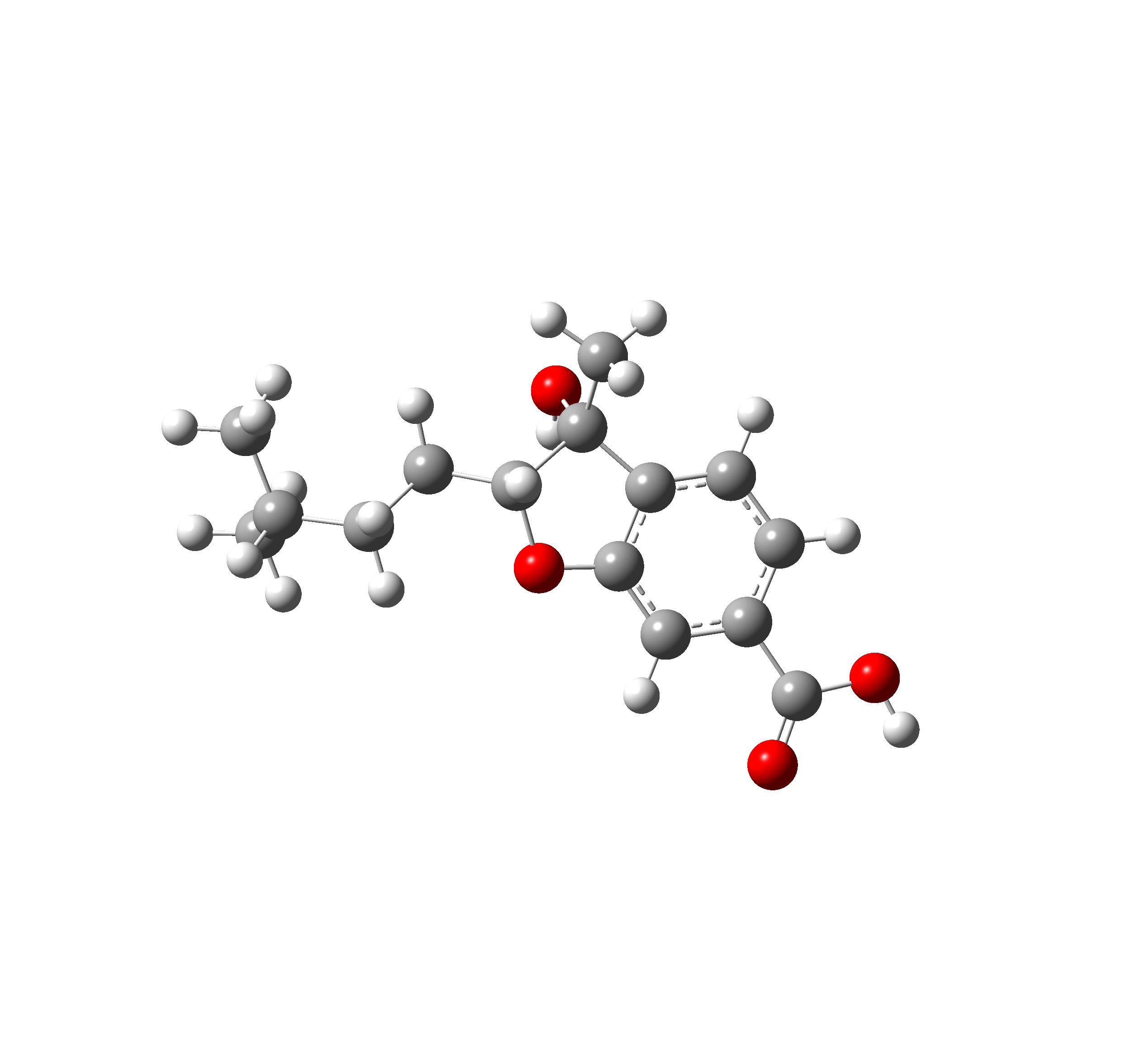 | 1.66 |
| 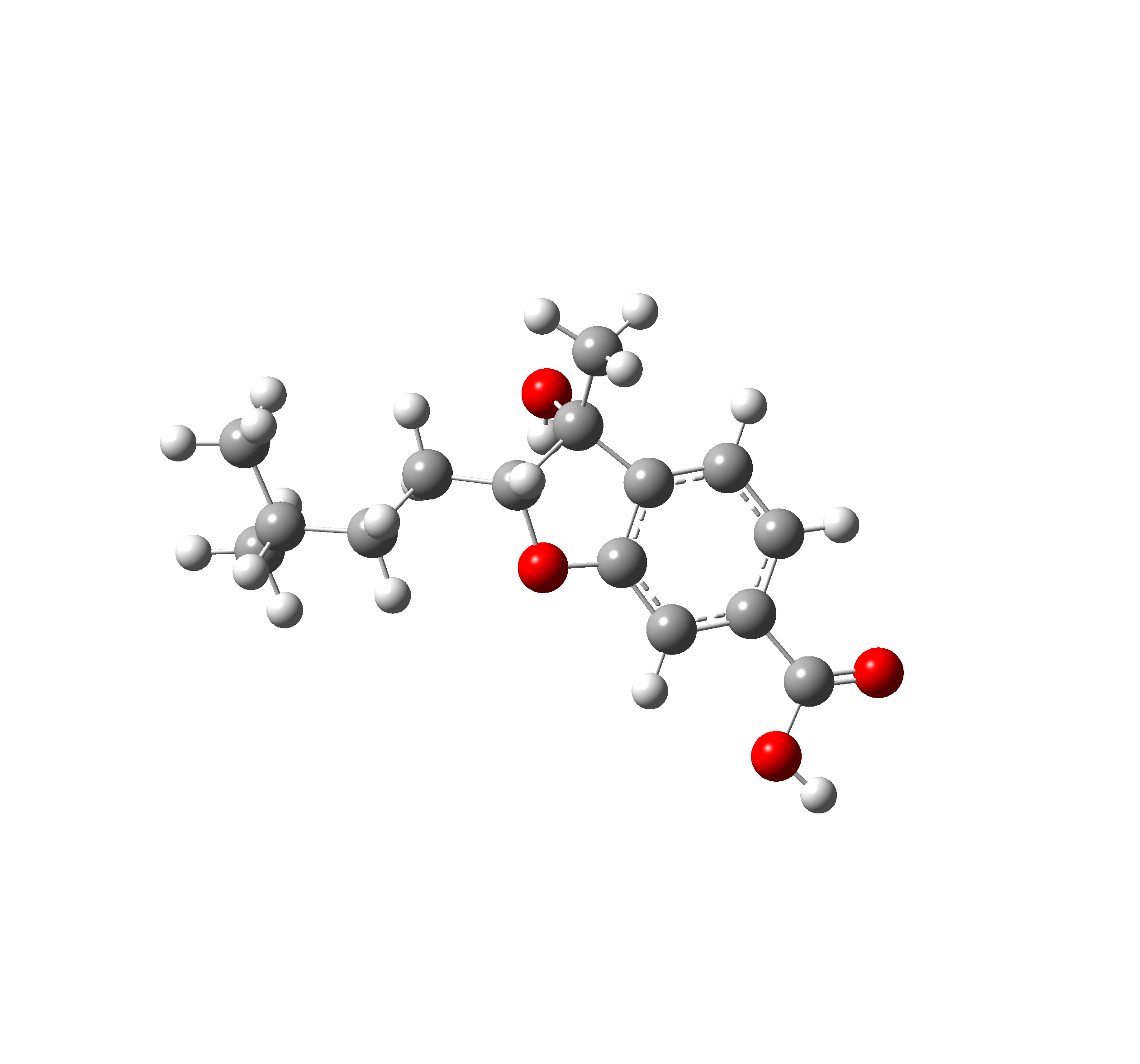 | 1.77 |
| 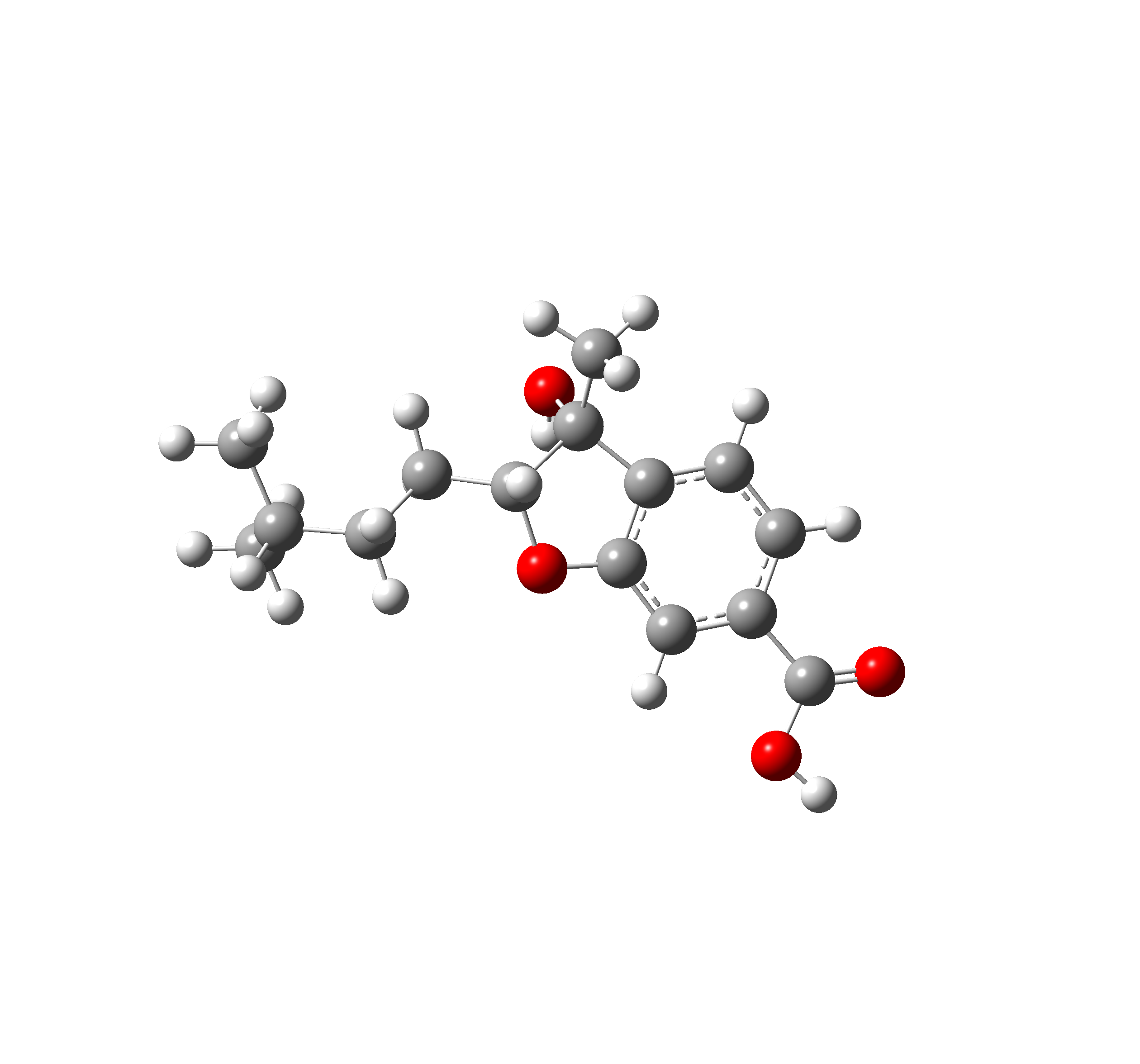 | 1.77 |
| 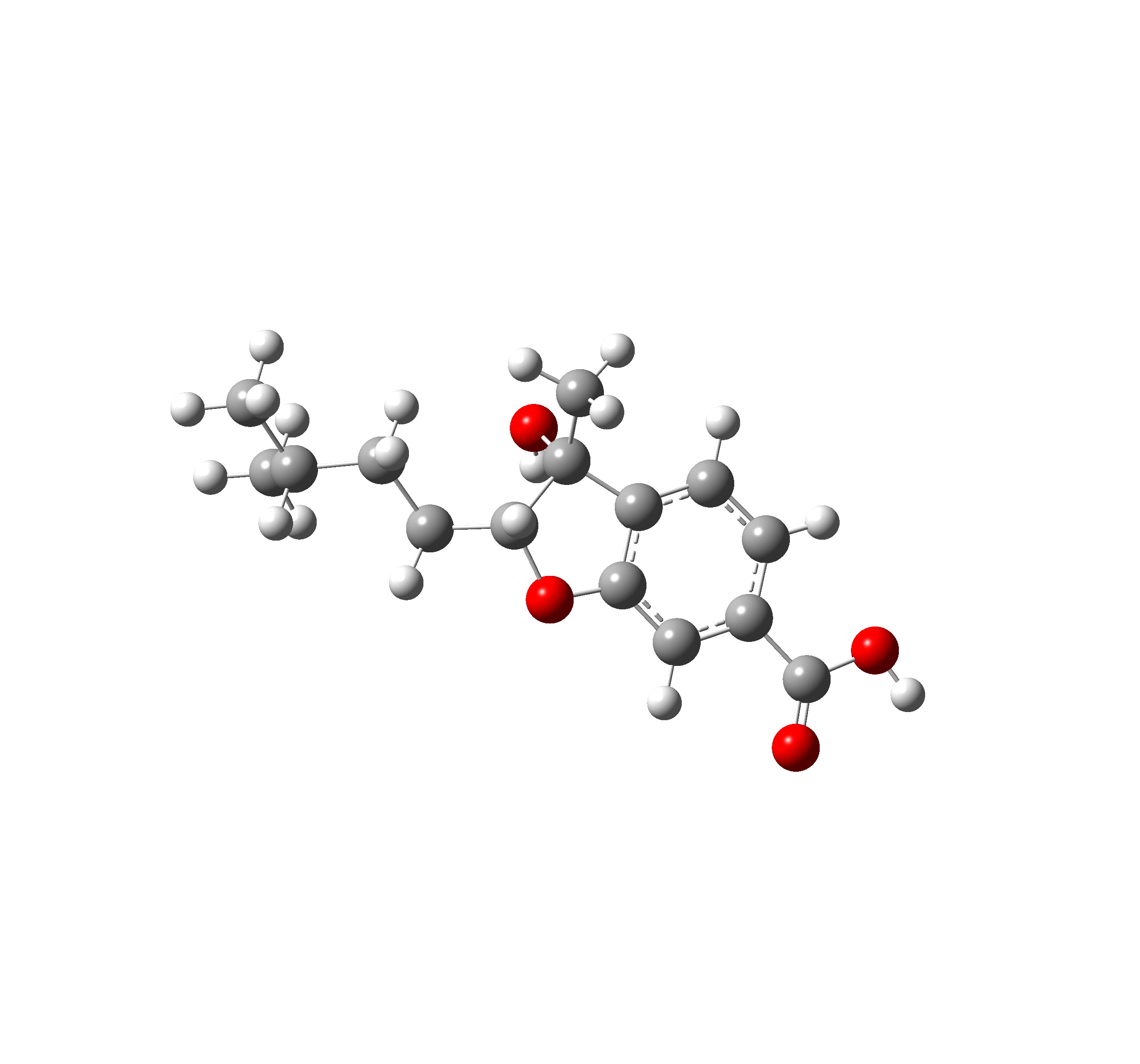 | 2.36 |
| 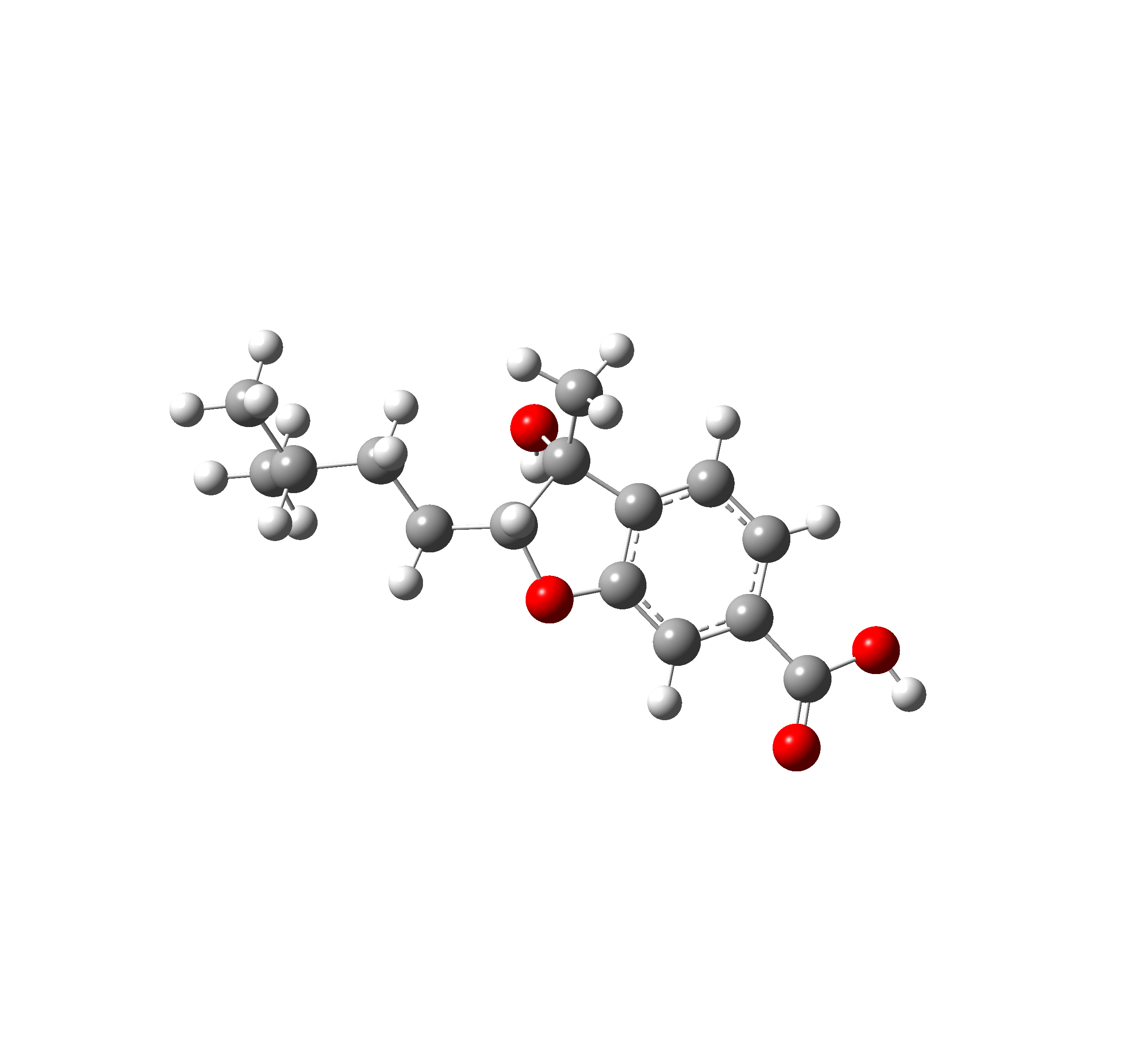 | 2.36 |
| 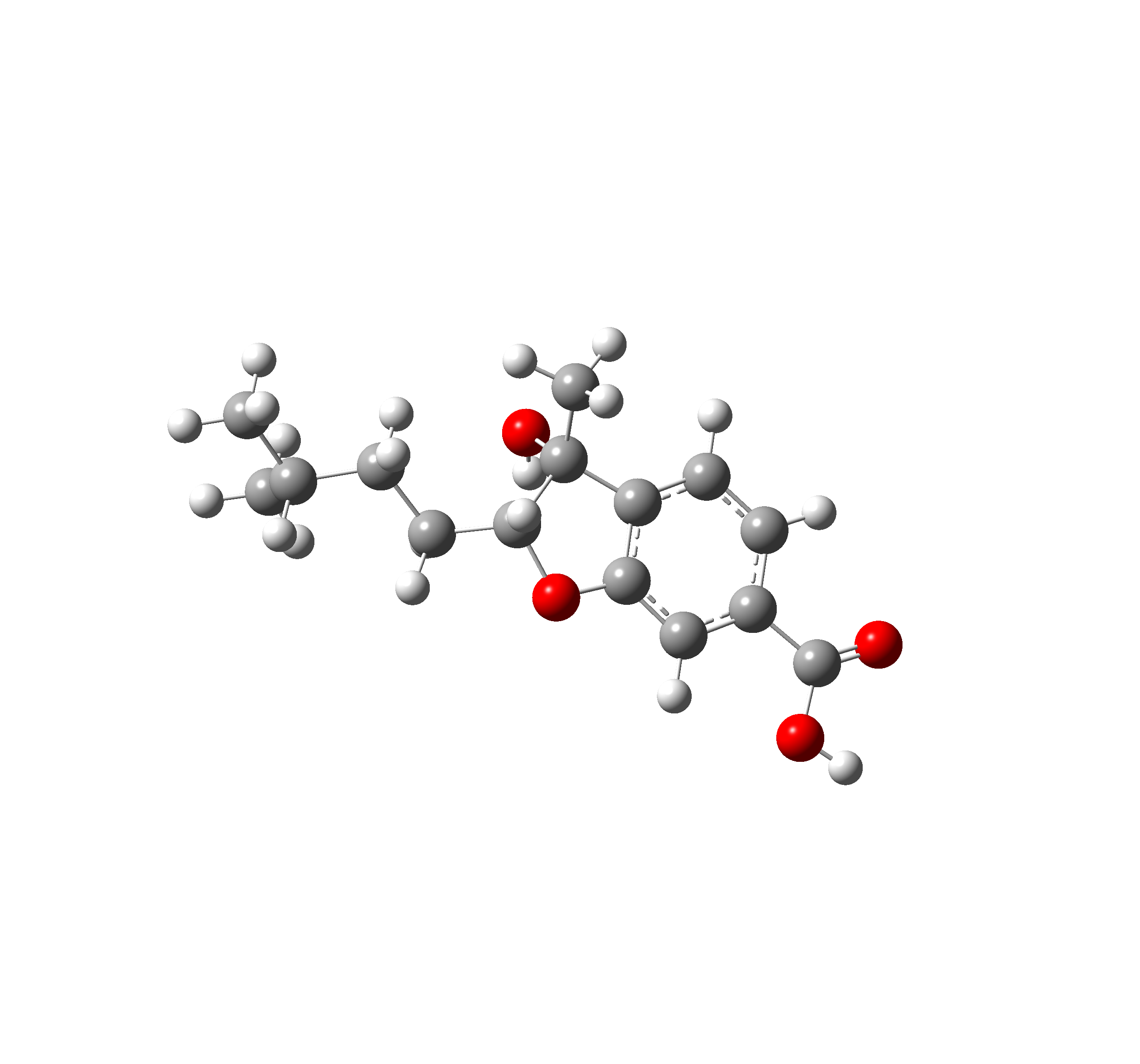 | 2.55 |
| 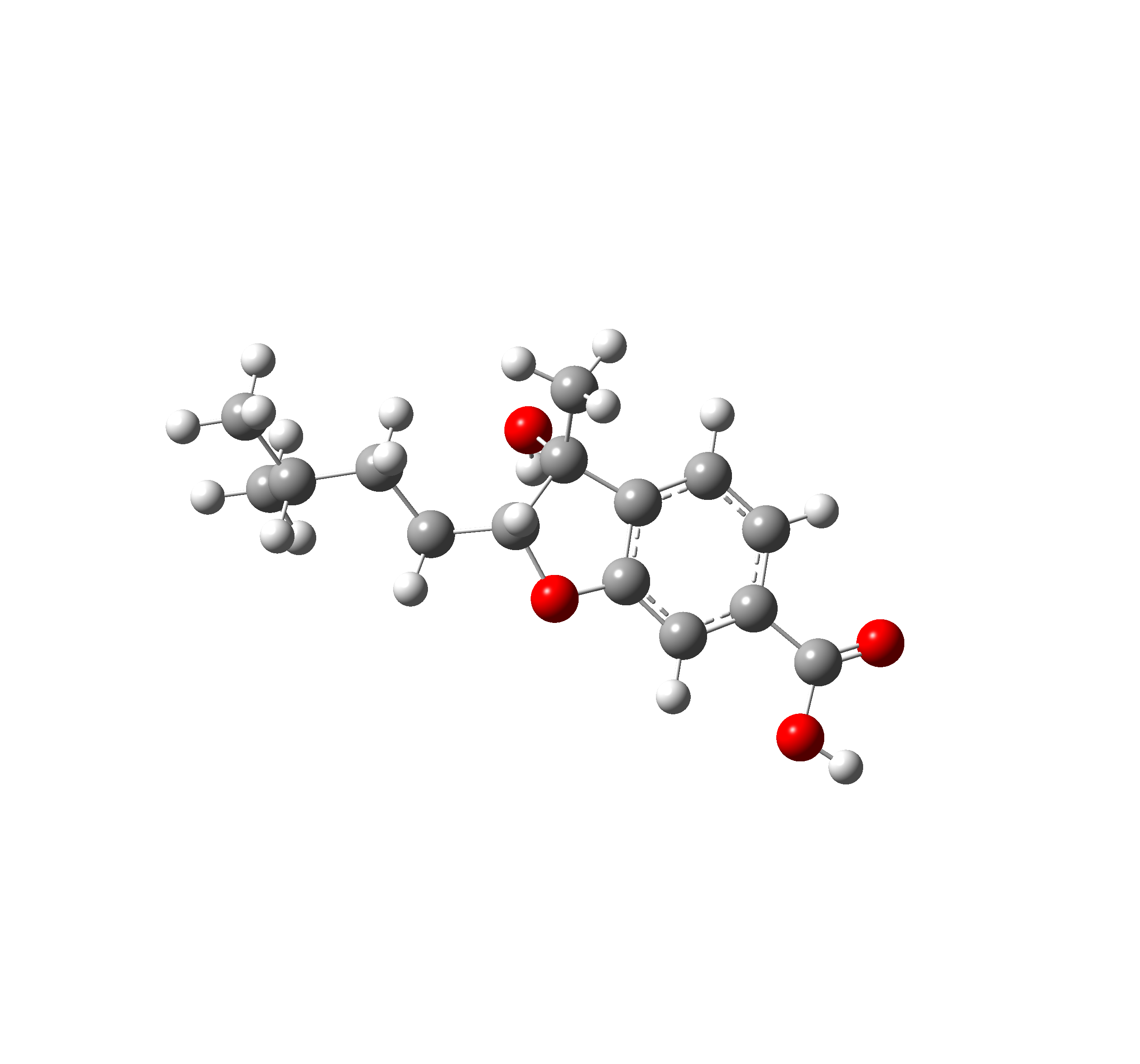 | 2.56 |
| 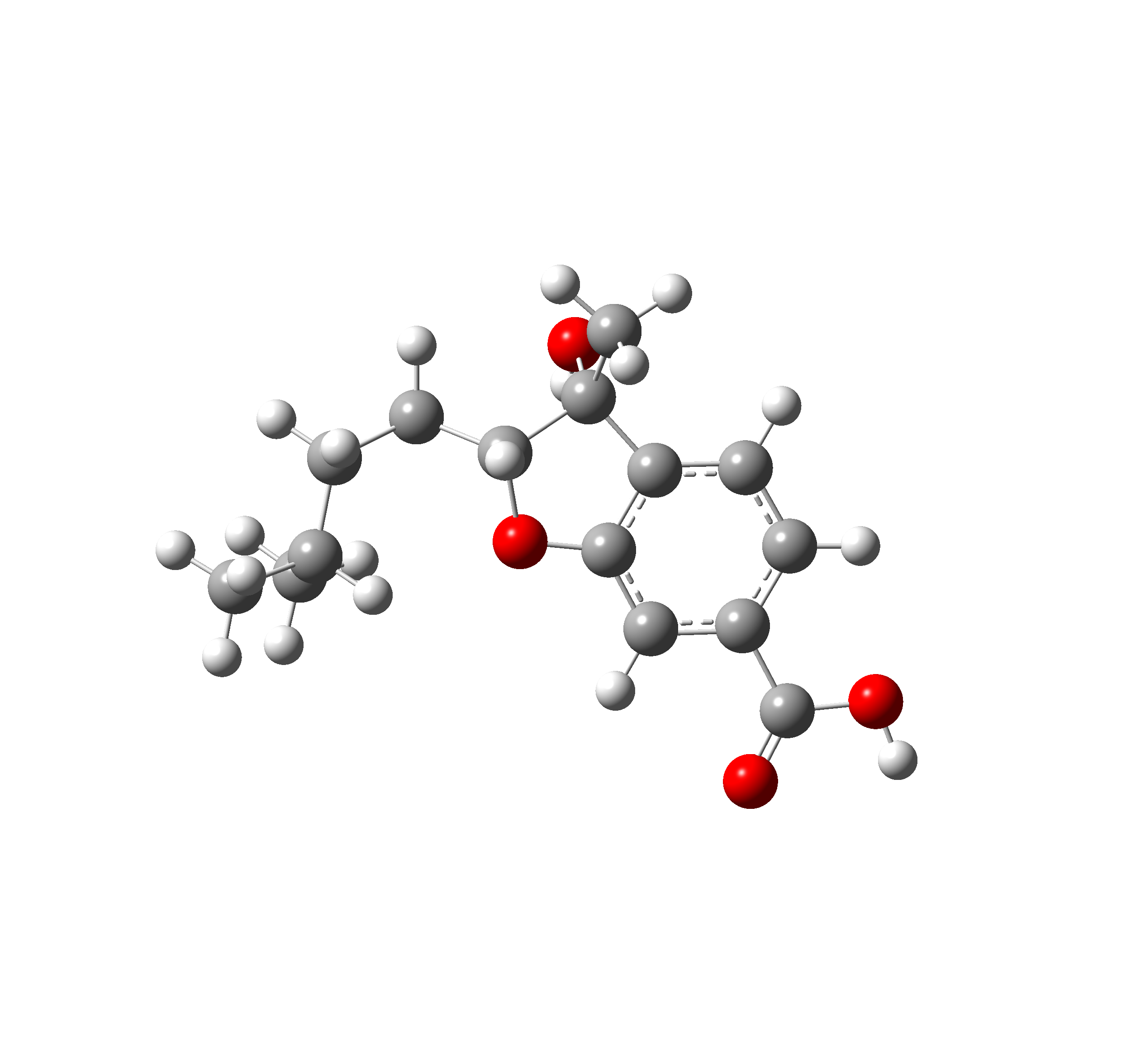 | 1.45 |
| 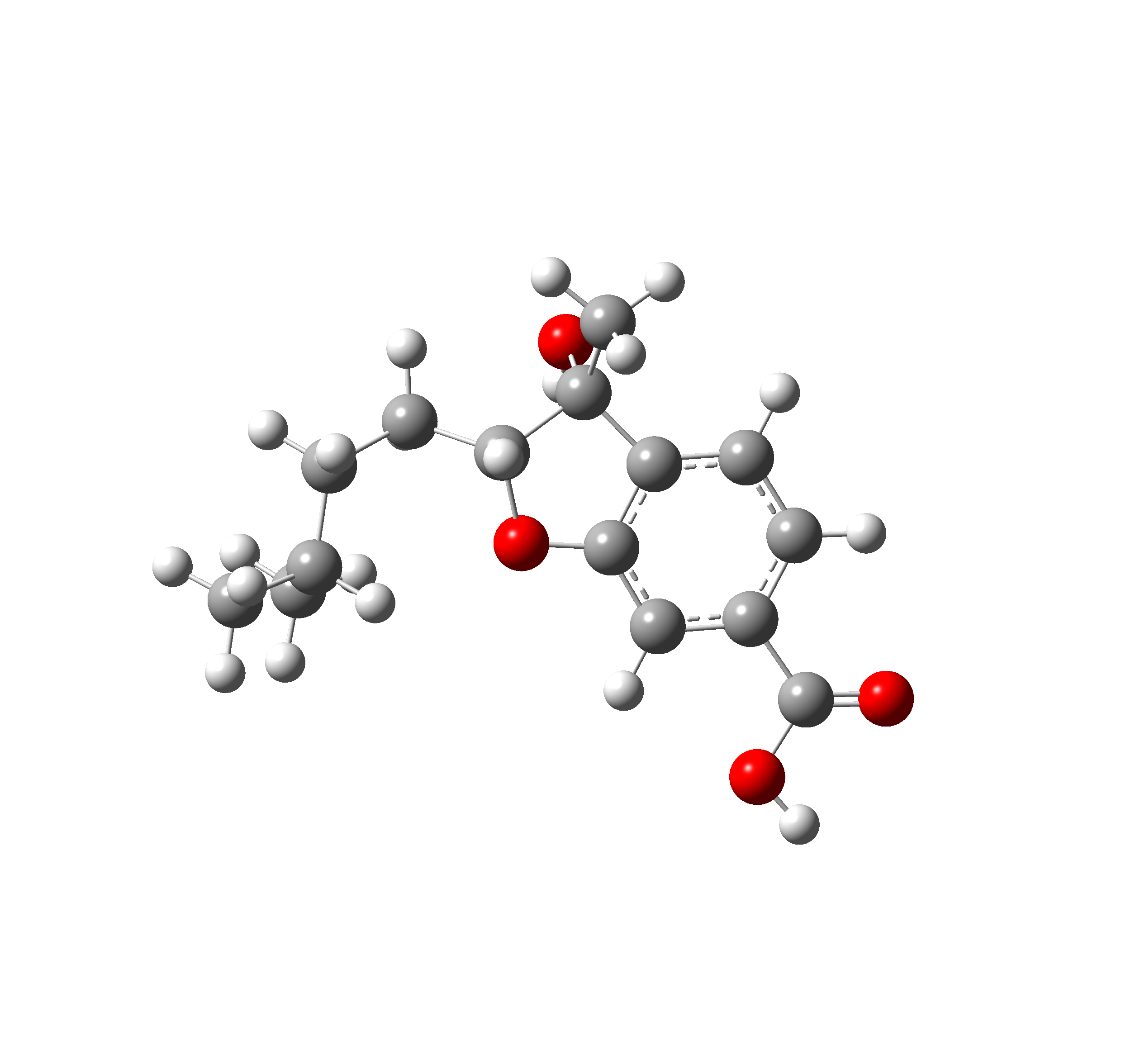 | 1.54 |
| 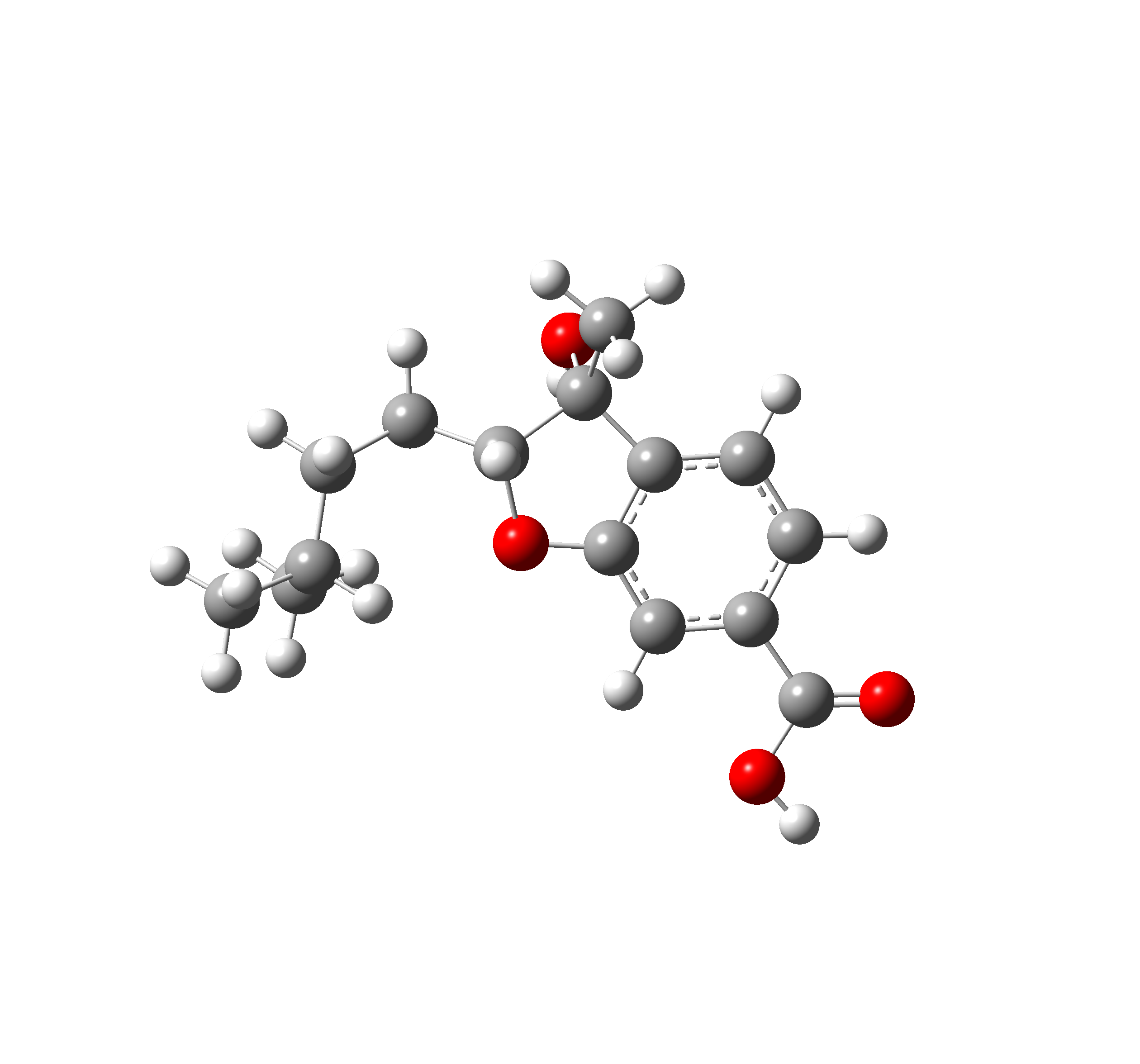 | 1.53 |
| 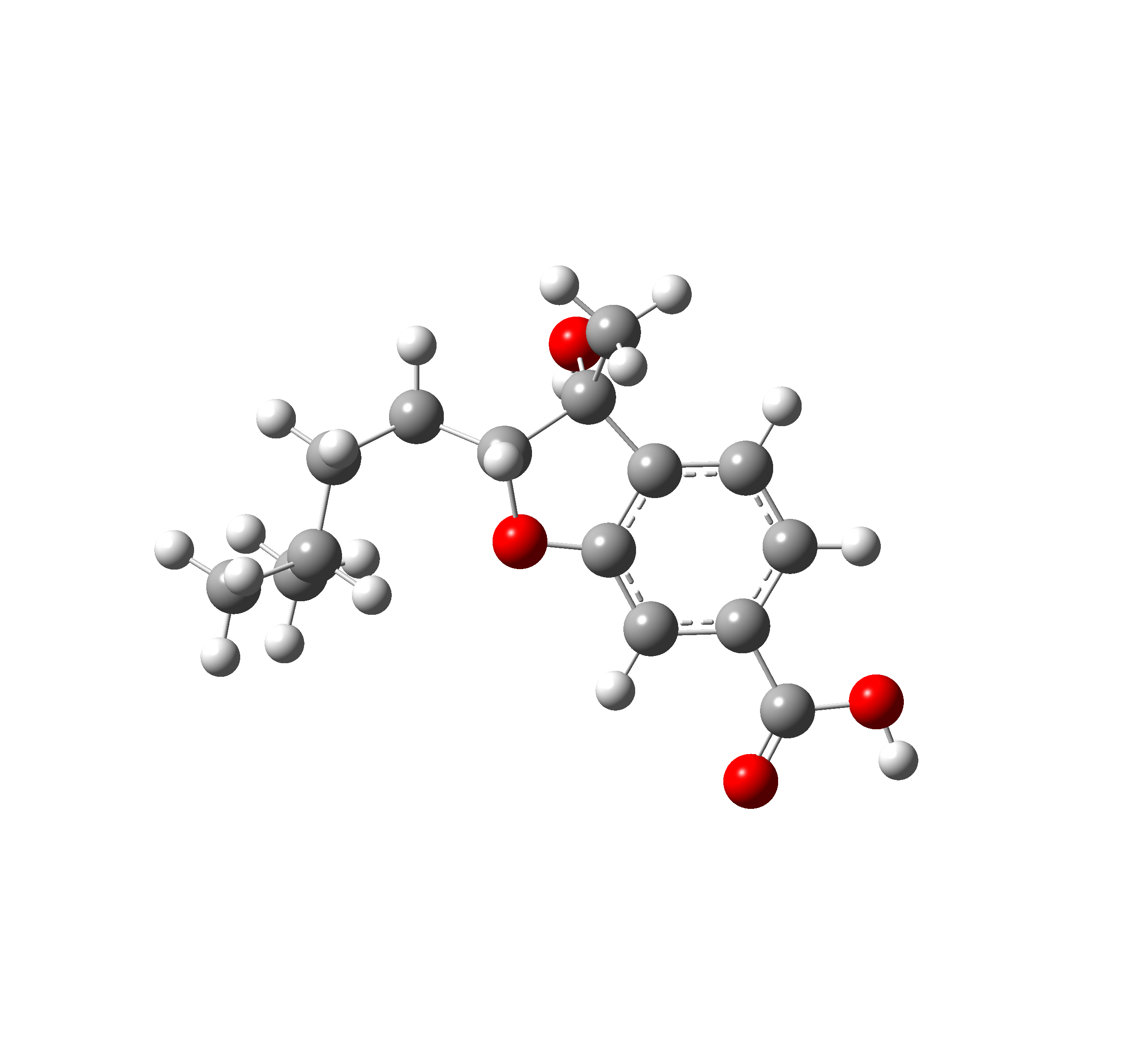 | 1.45 |
| 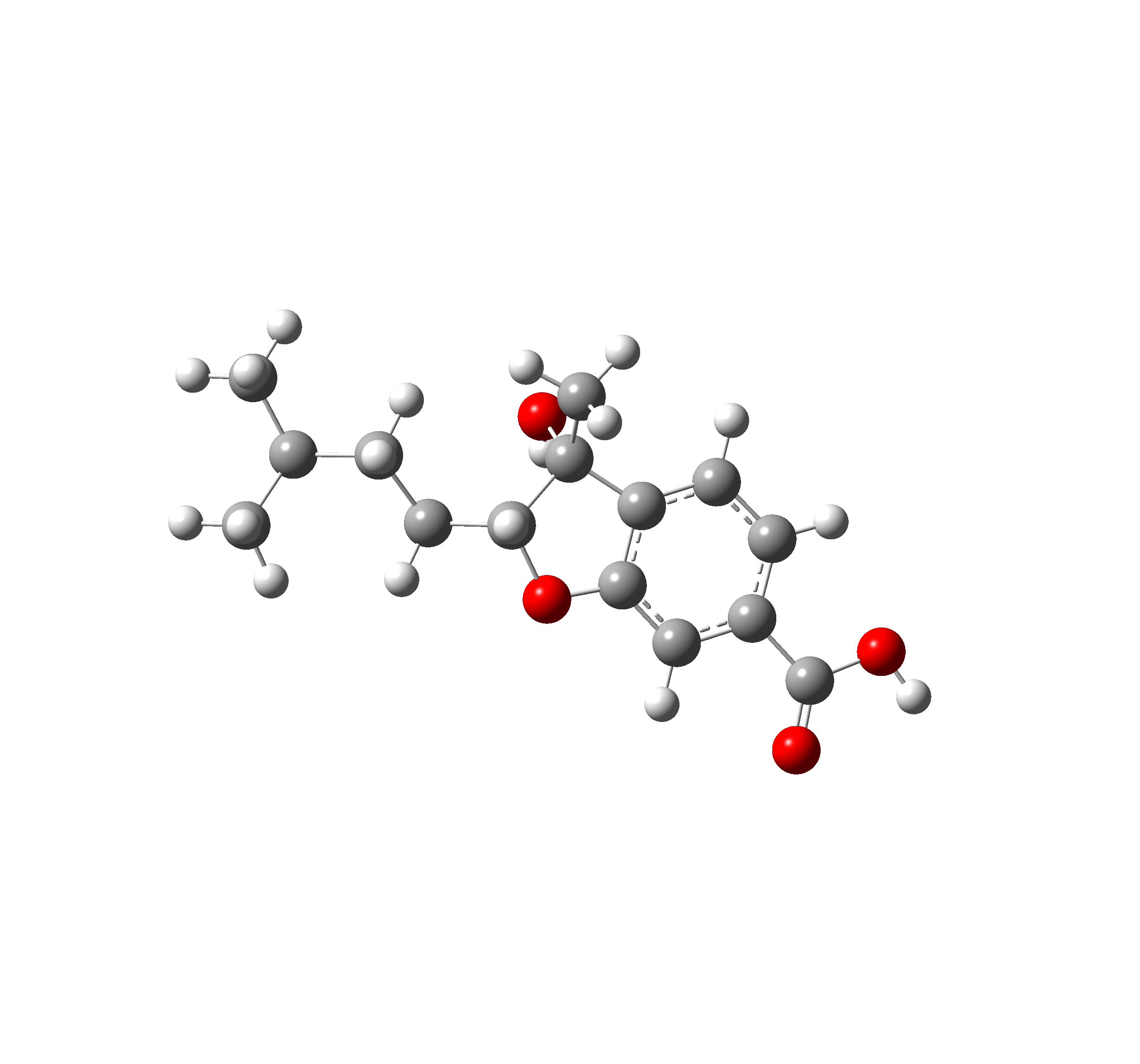 | 2.09 |
| 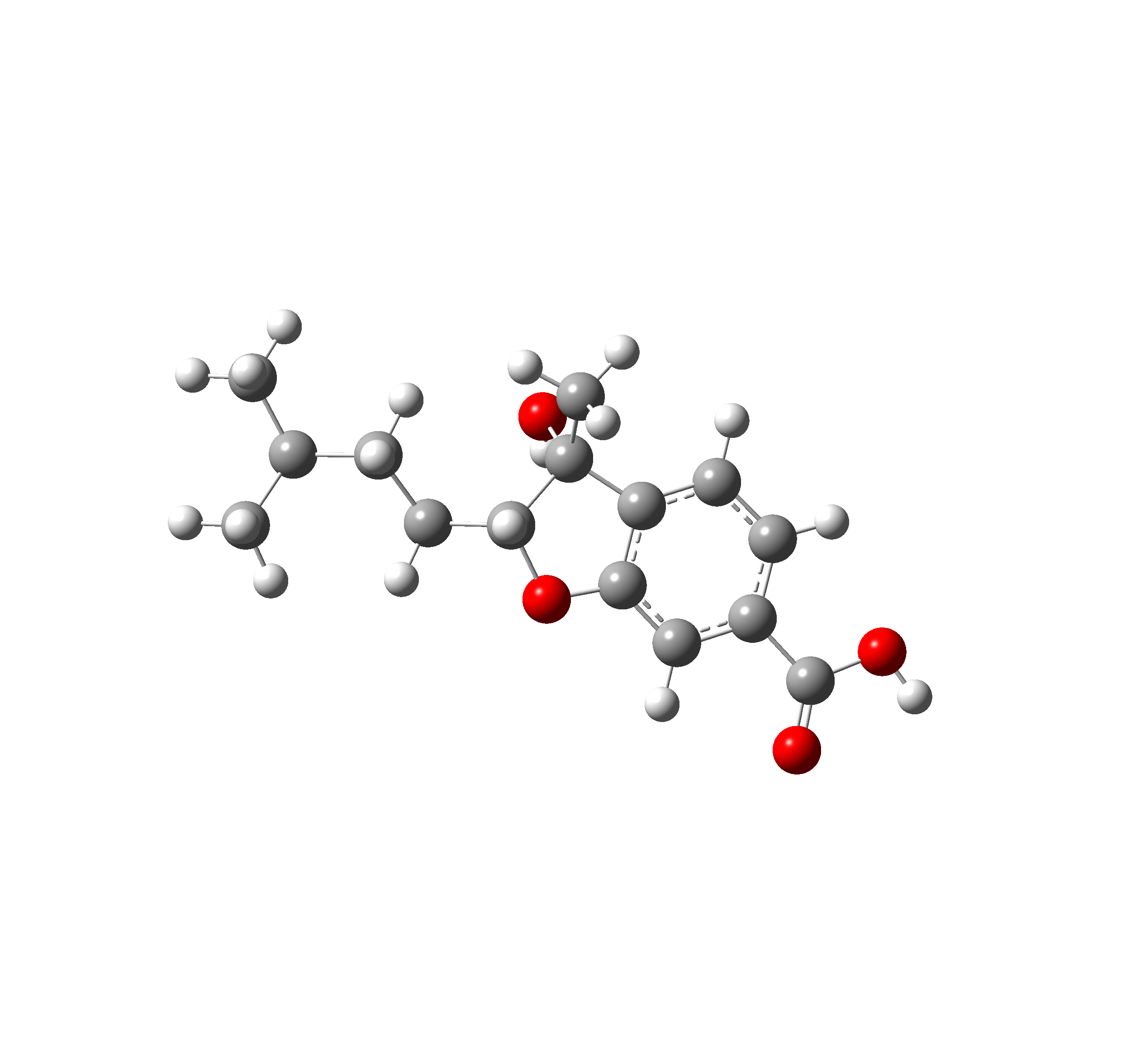 | 2.09 |
| 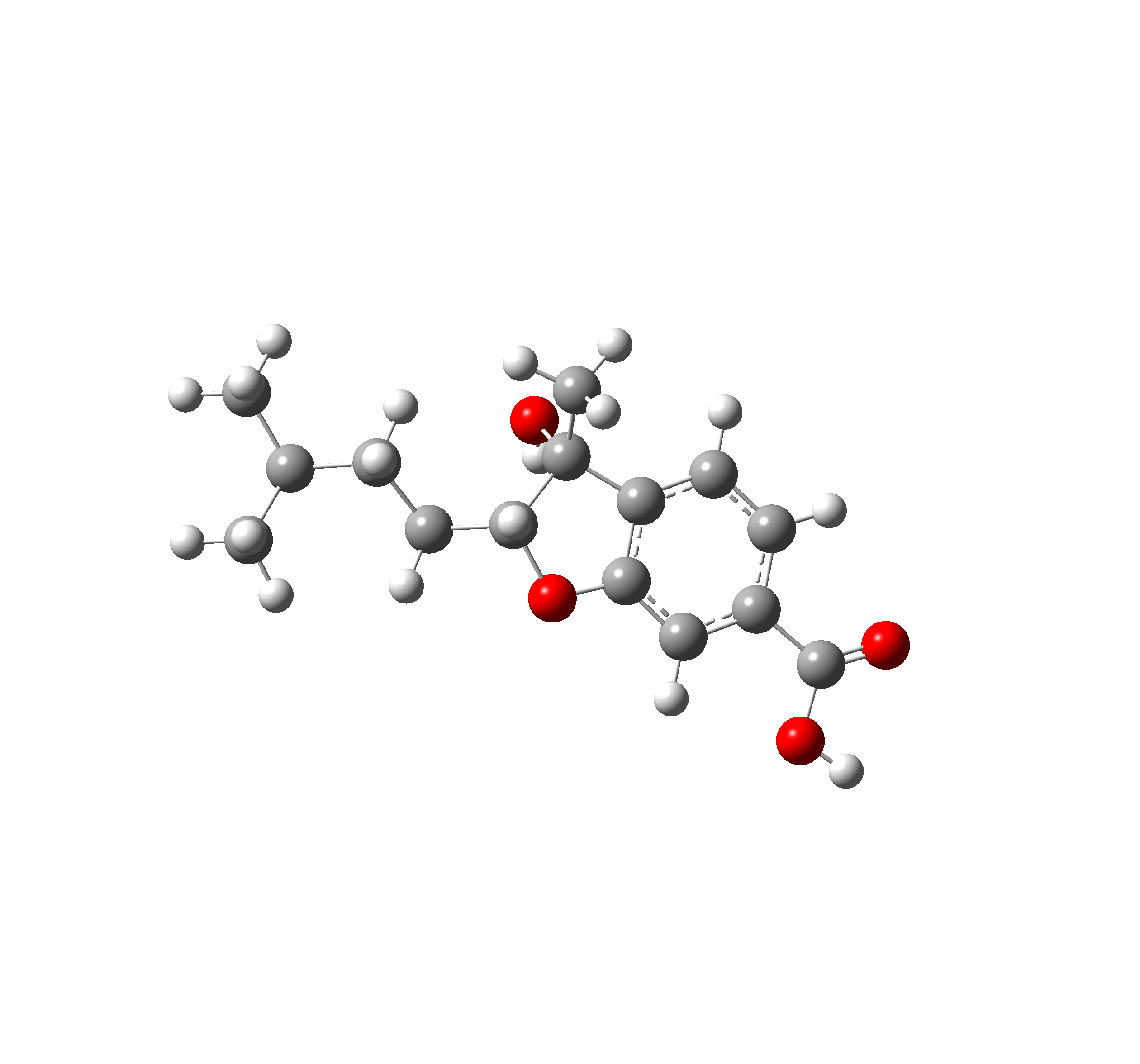 | 2.28 |
| 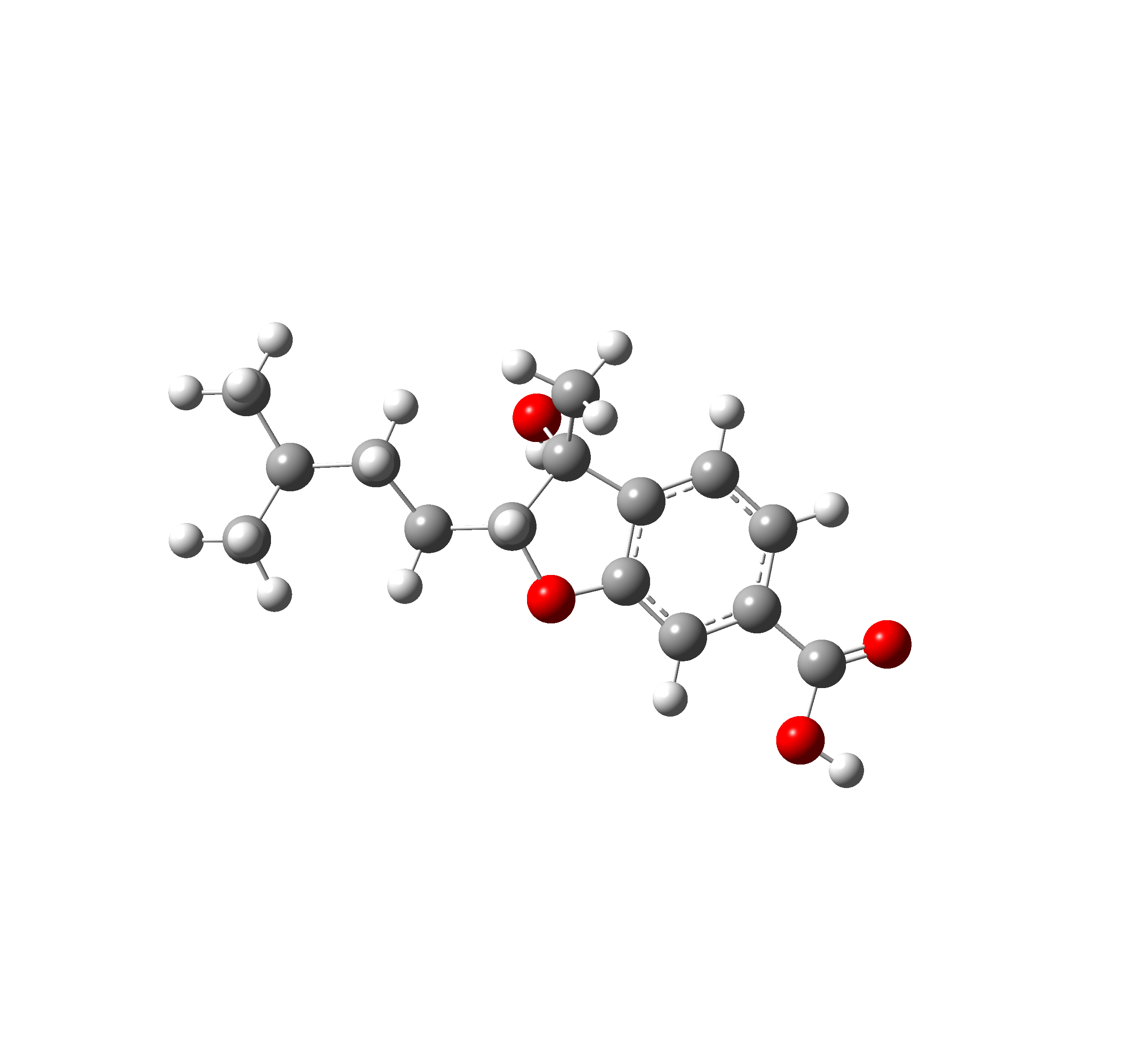 | 2.27 |

**Figure S17.** ECD conformers of aspersydonol A (**1**).

|  | |
| --- | --- |
| Conformers | Populations (%) |
| 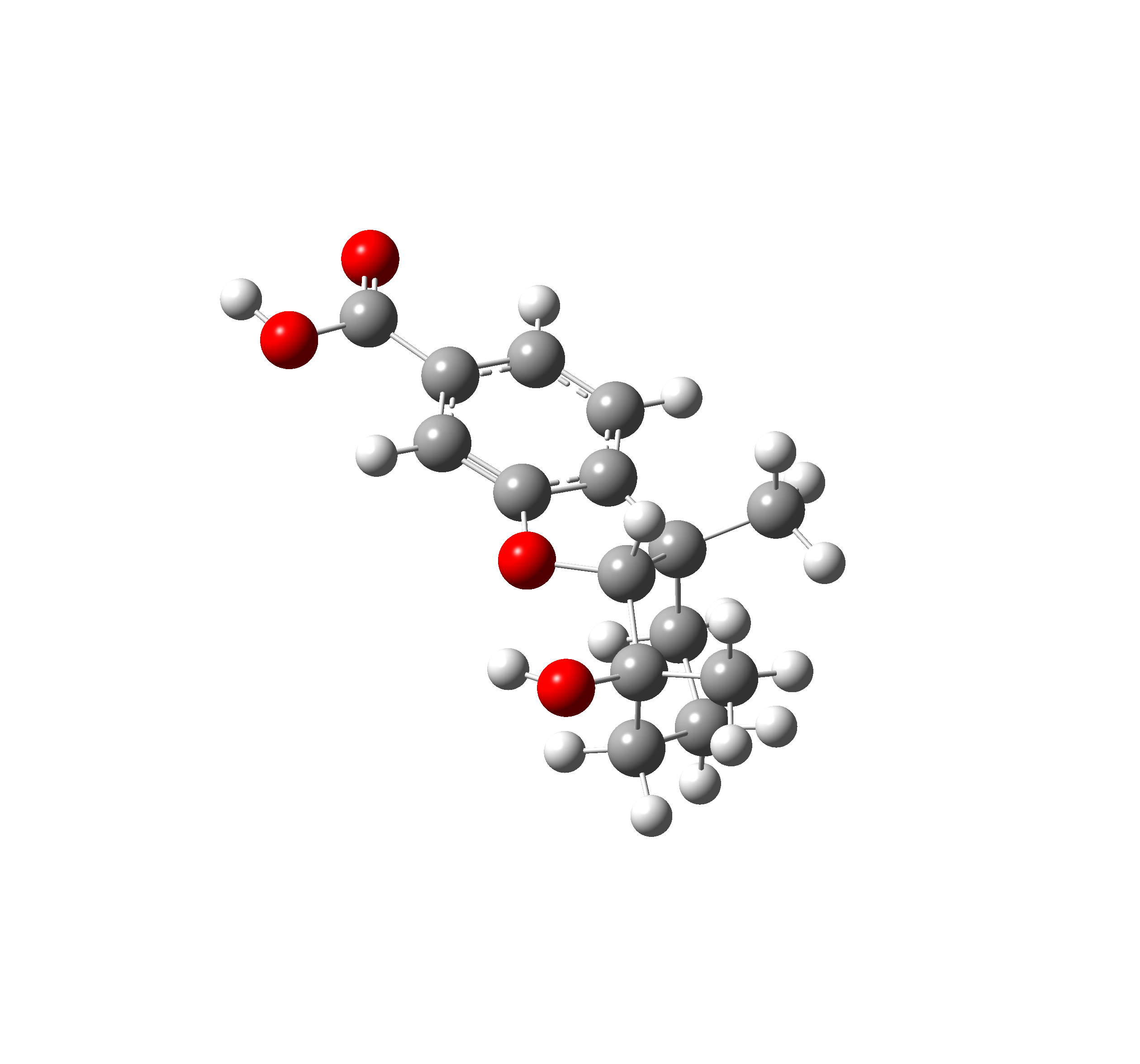 | 5.87 |
| 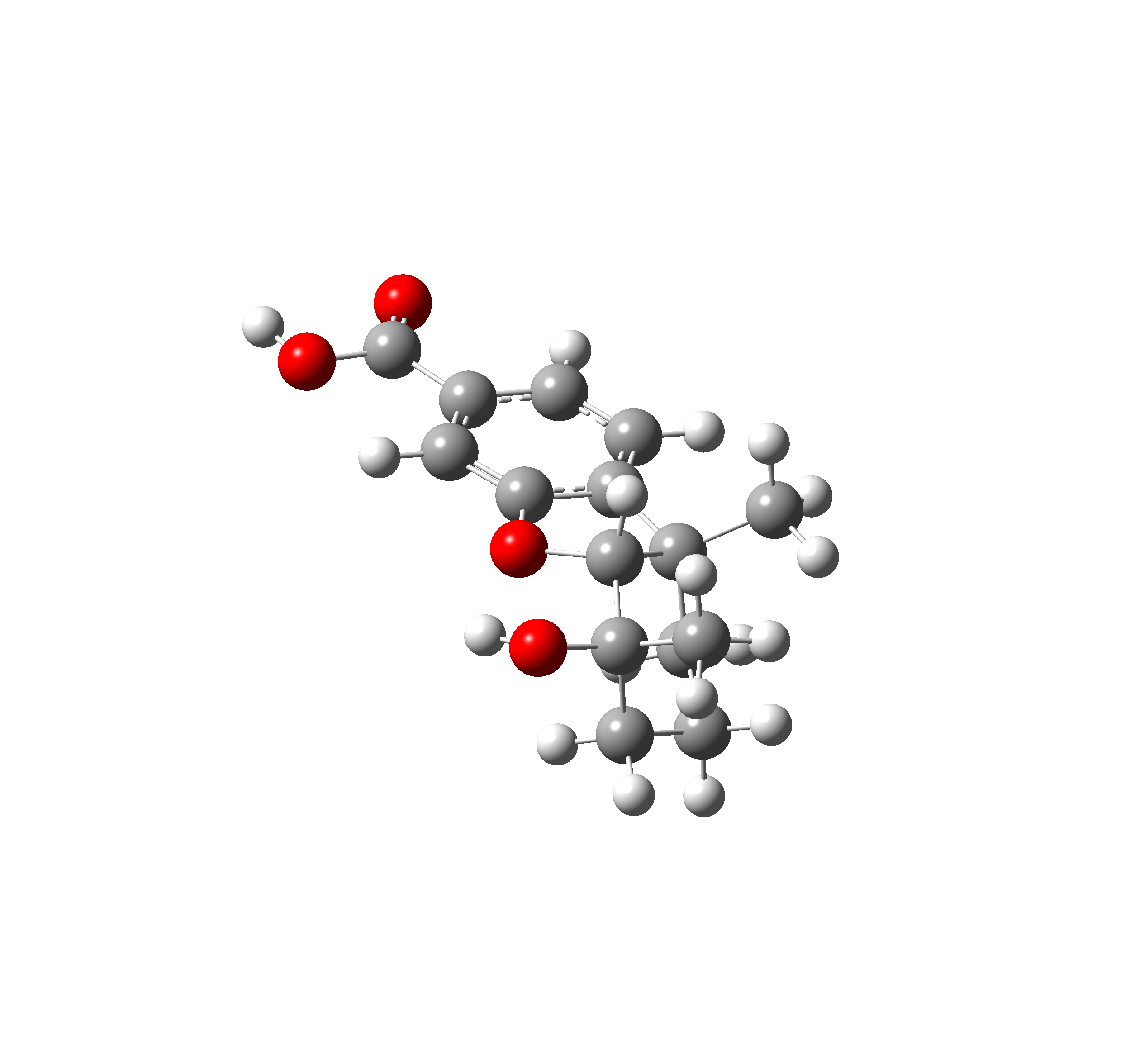 | 5.85 |
| 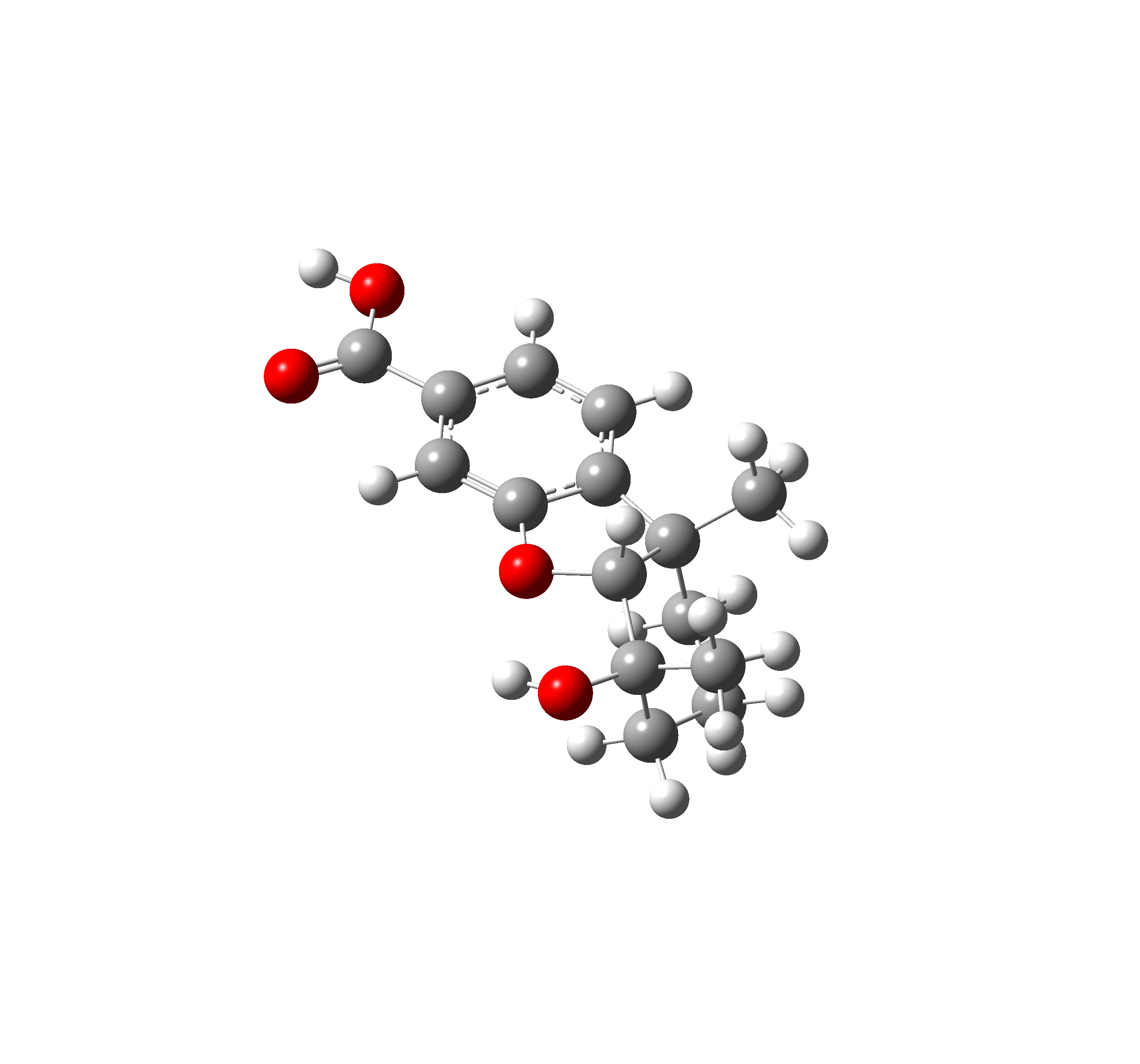 | 5.64 |
| 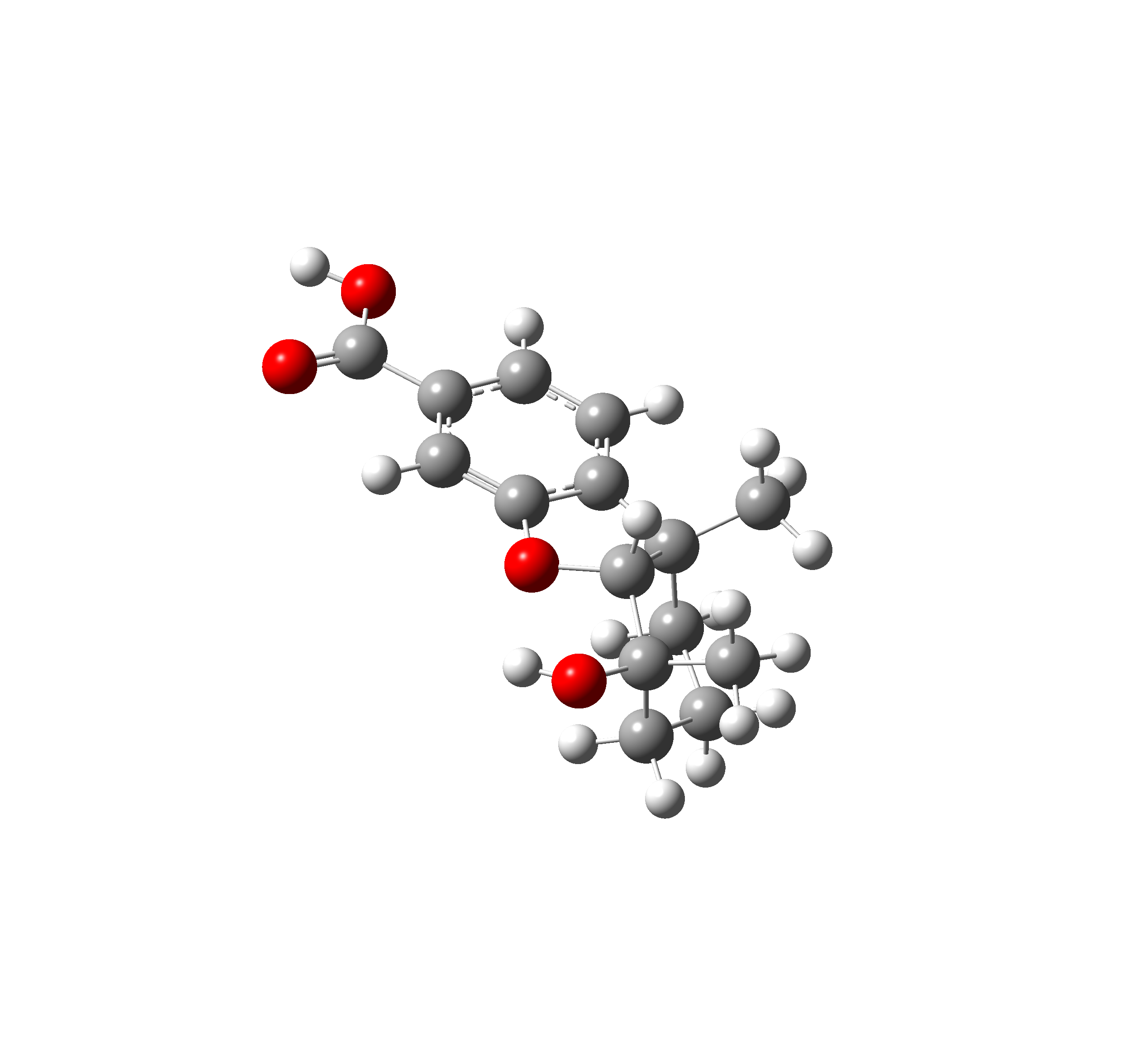 | 5.62 |
| 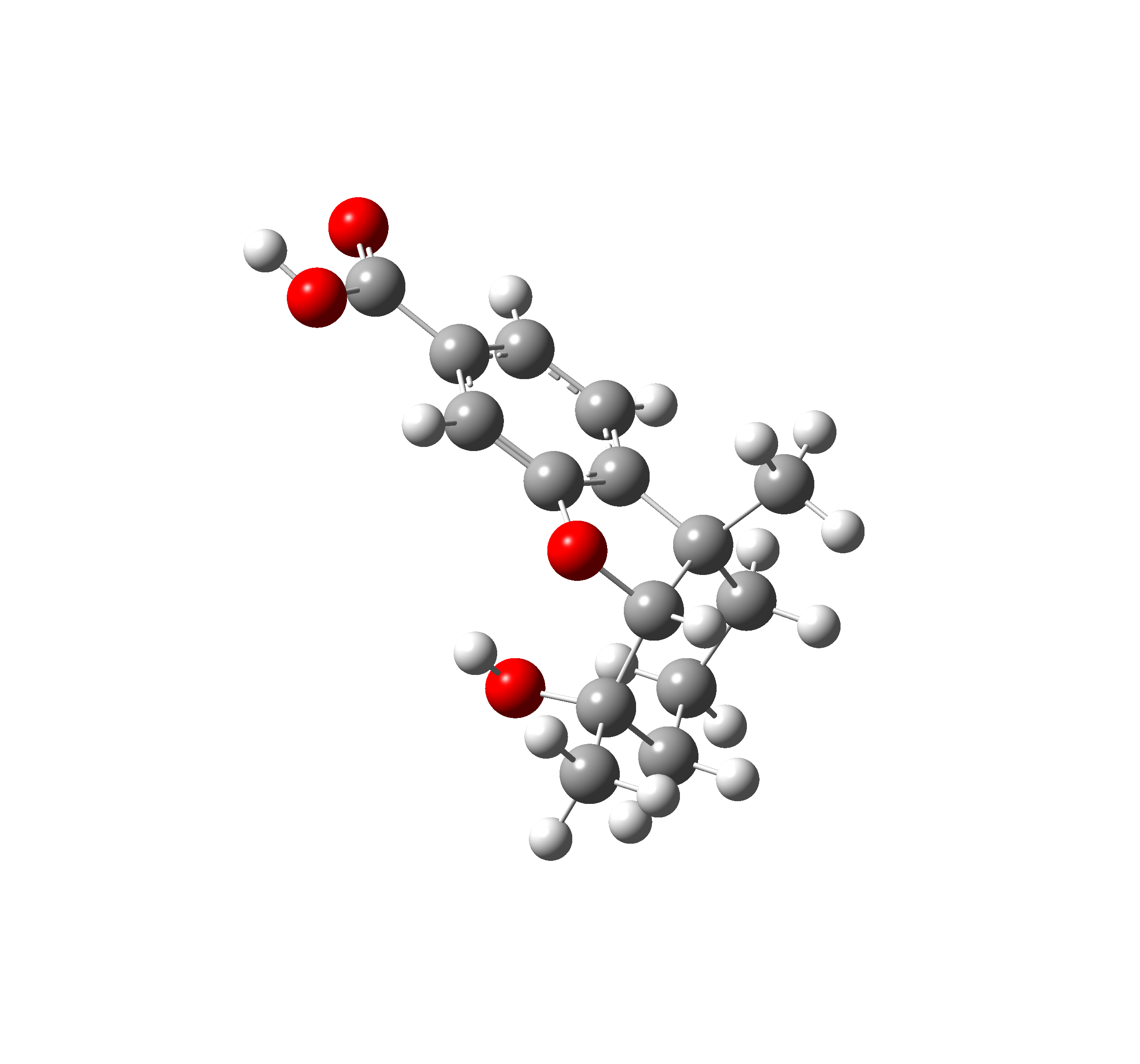 | 9.89 |
| 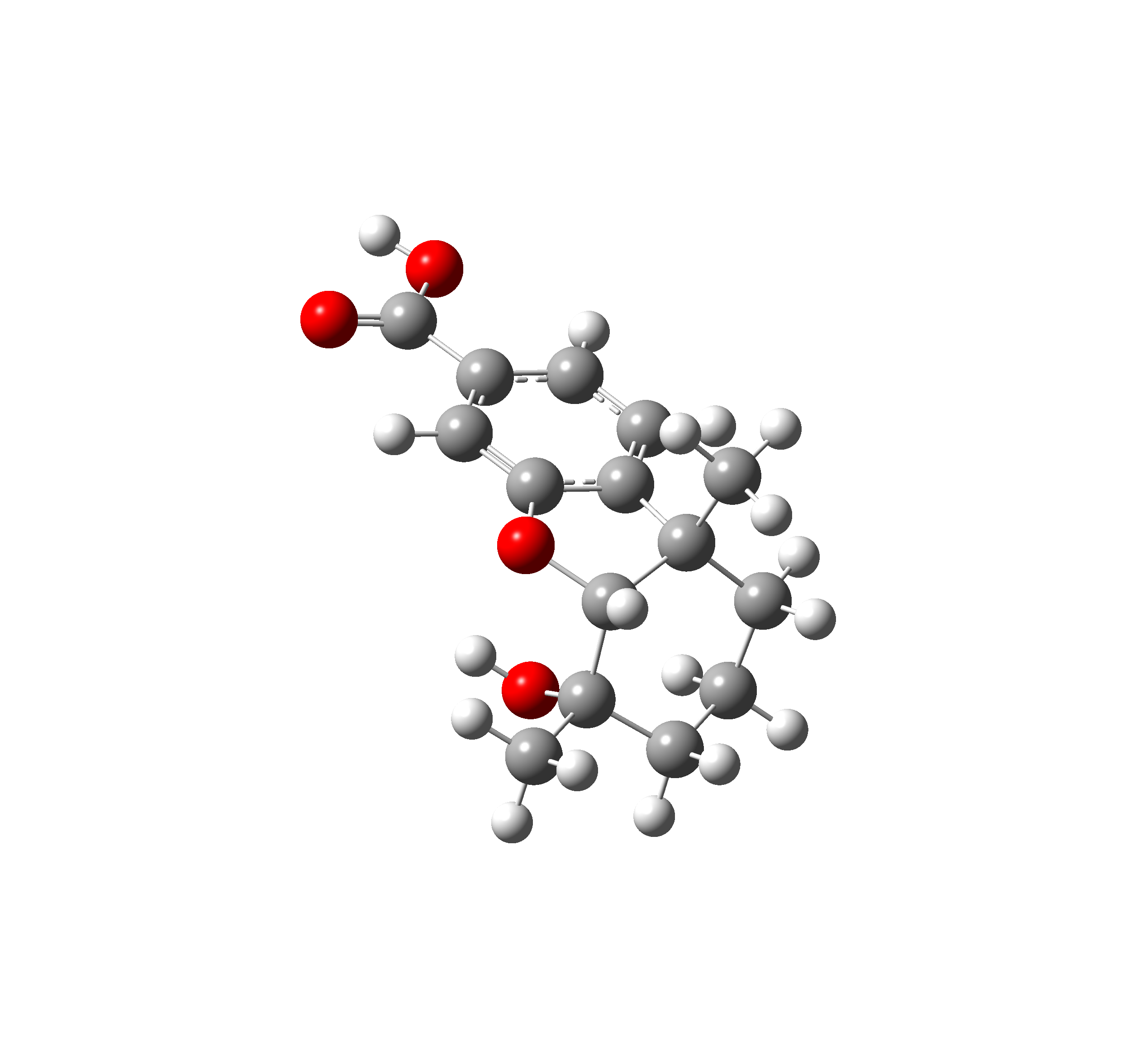 | 10.87 |
| 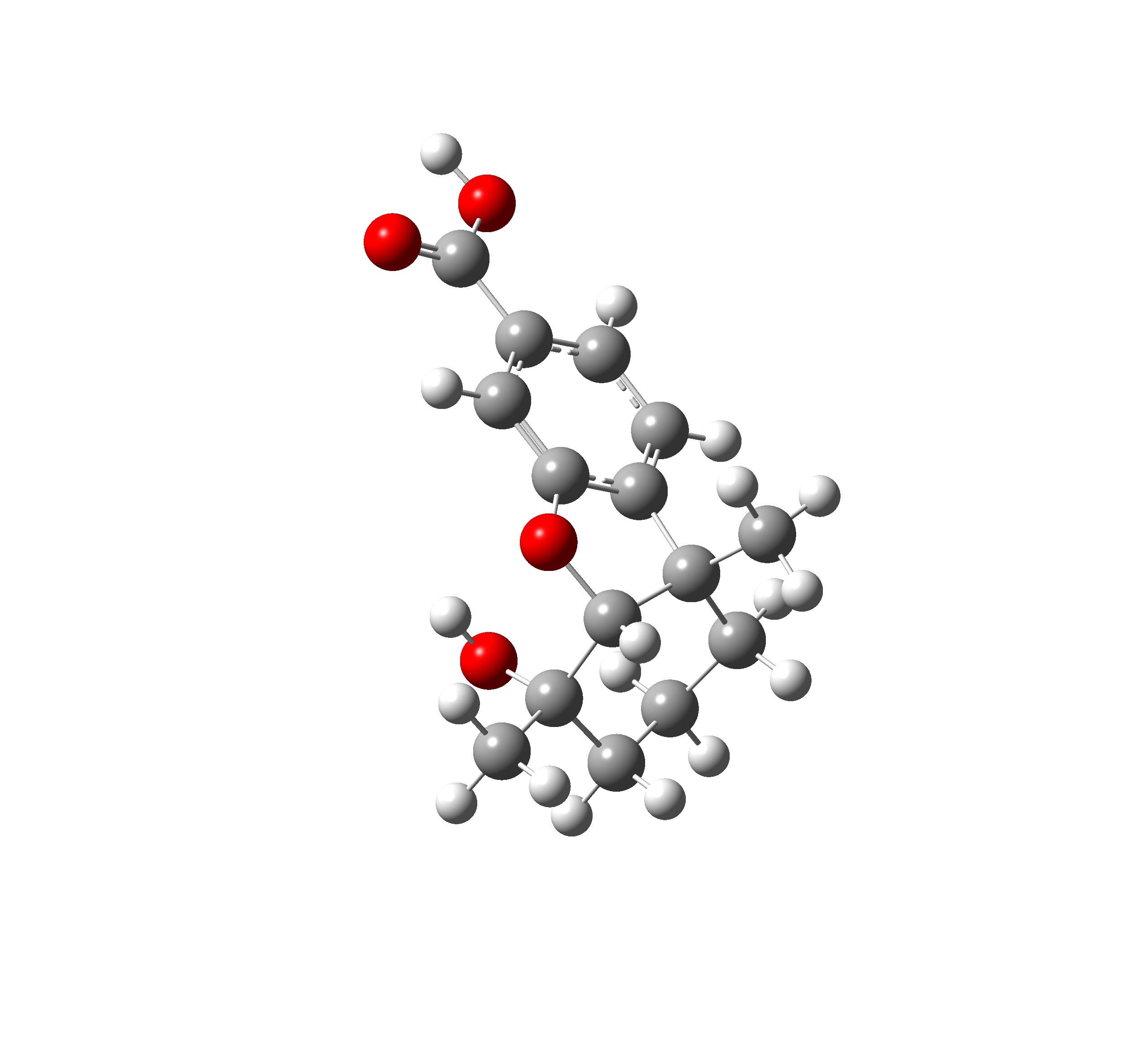 | 10.86 |
| 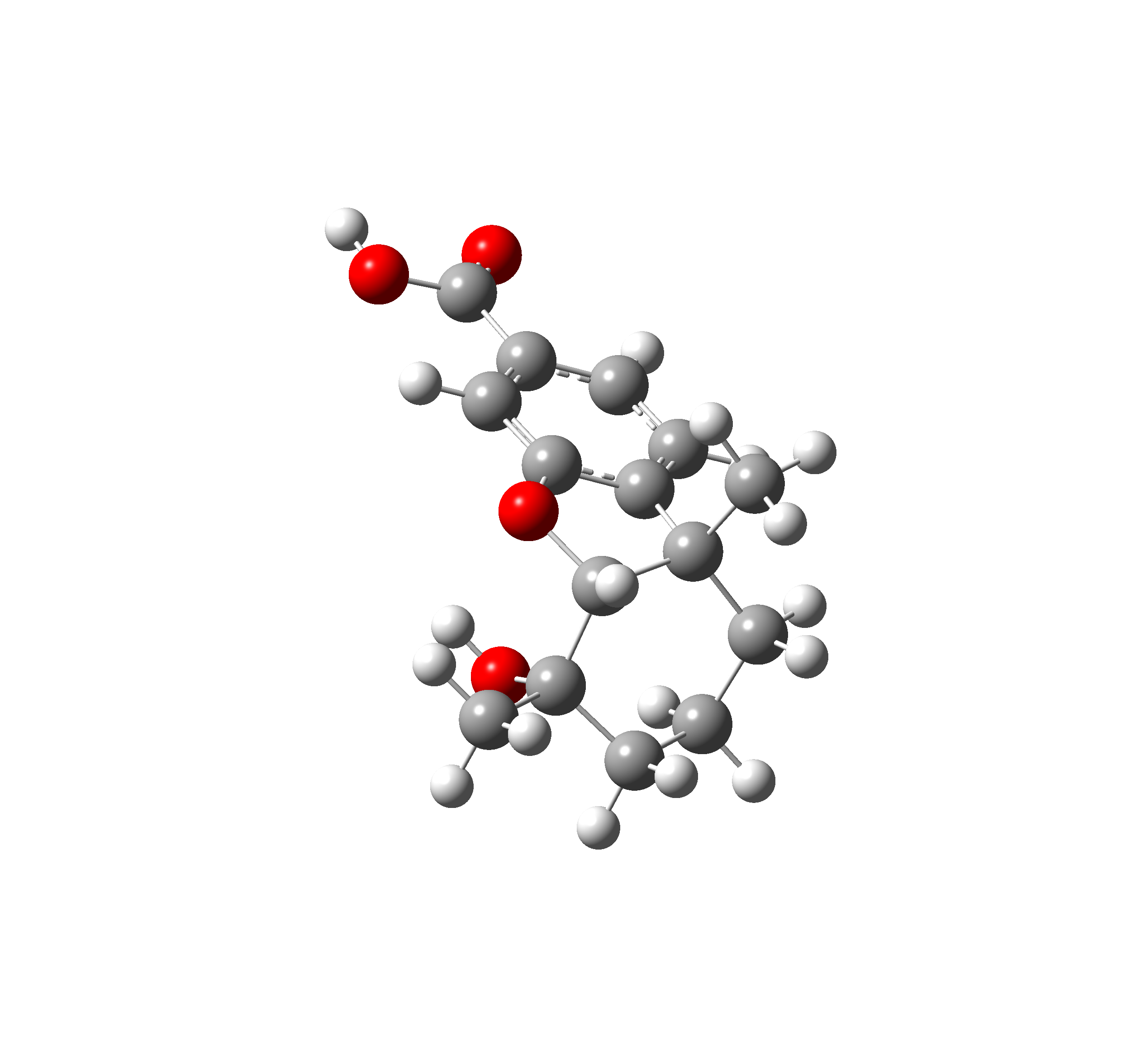 | 9.92 |
| 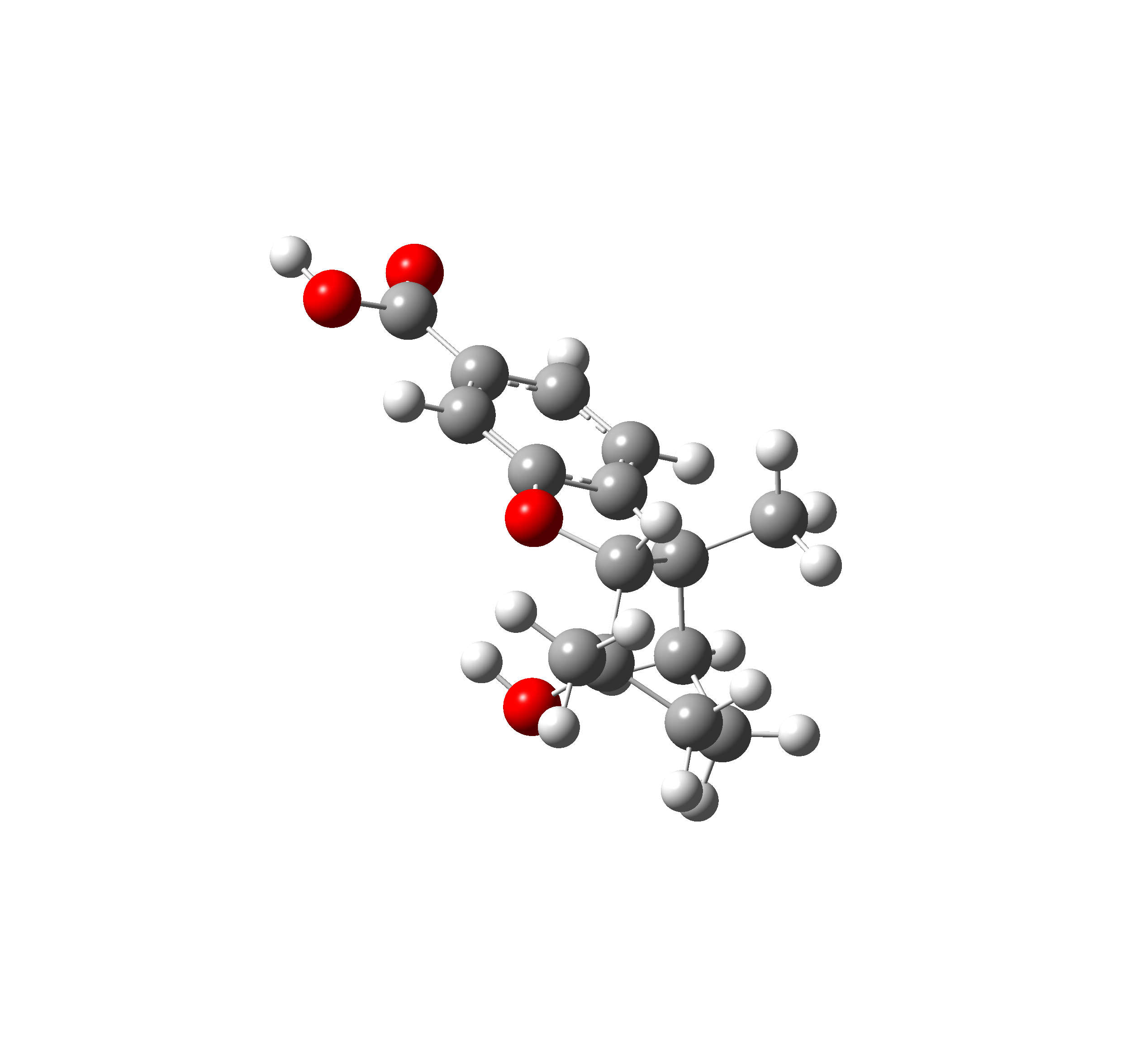 | 8.82 |
| 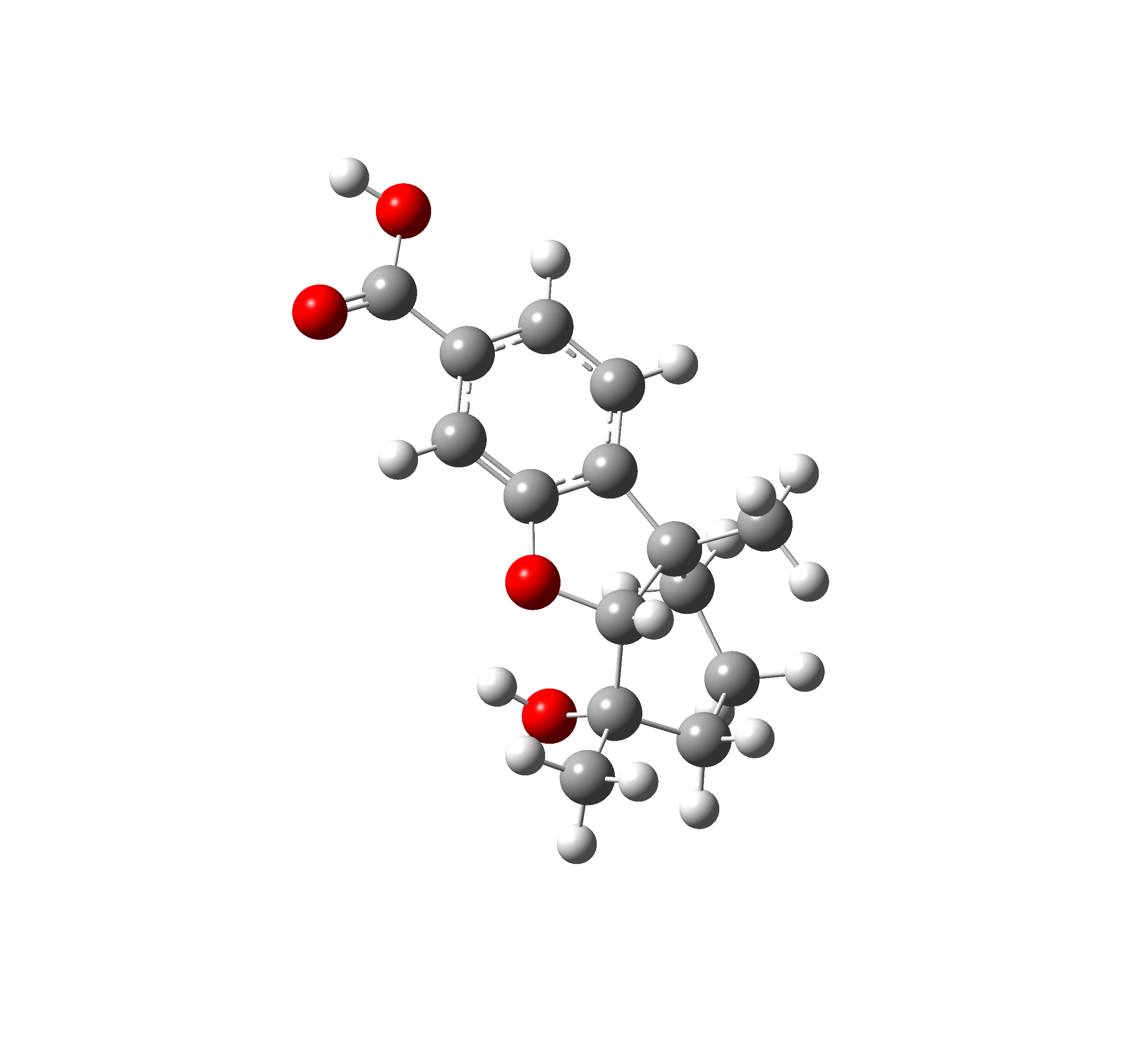 | 8.96 |
| 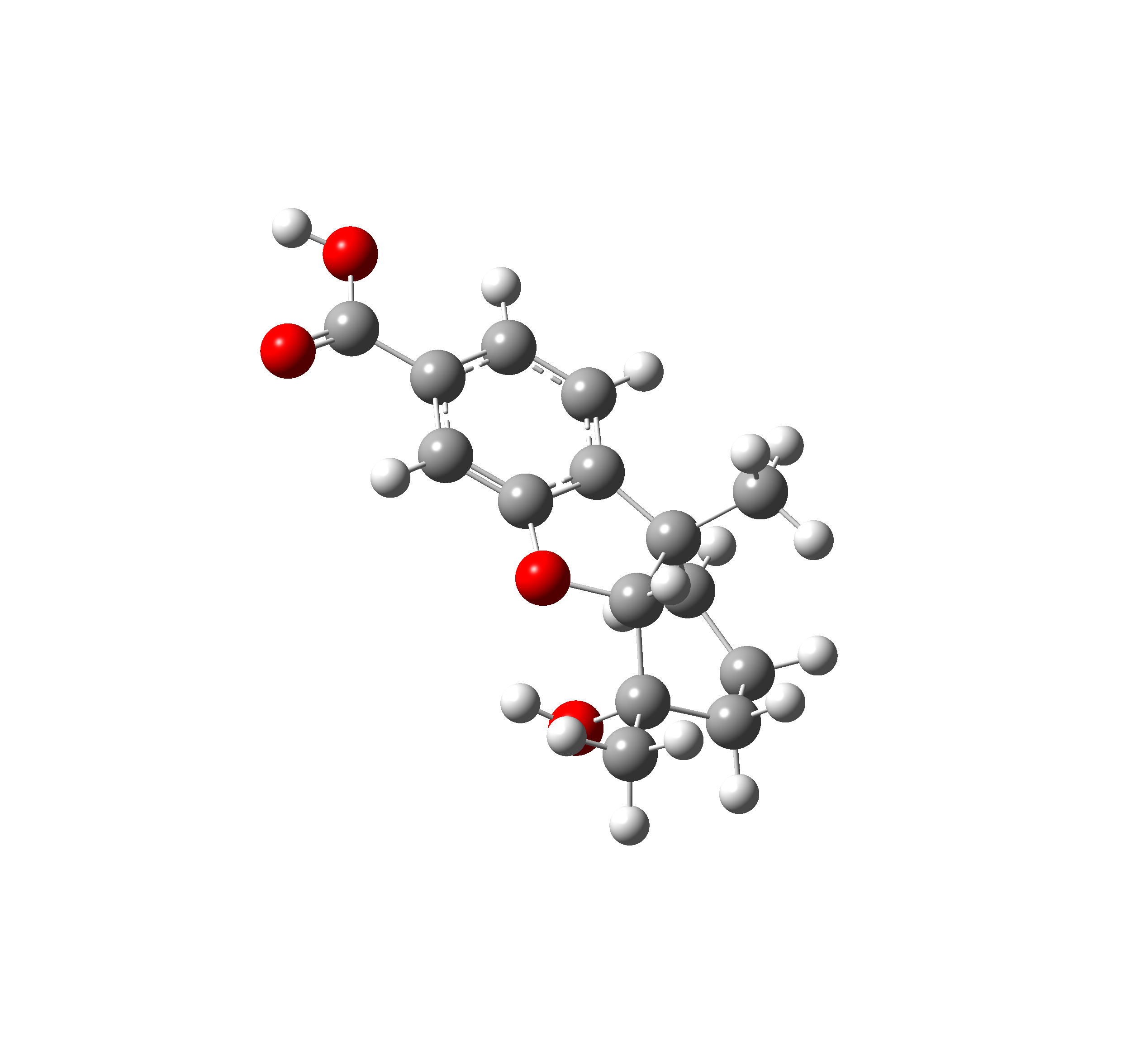 | 8.91 |
| 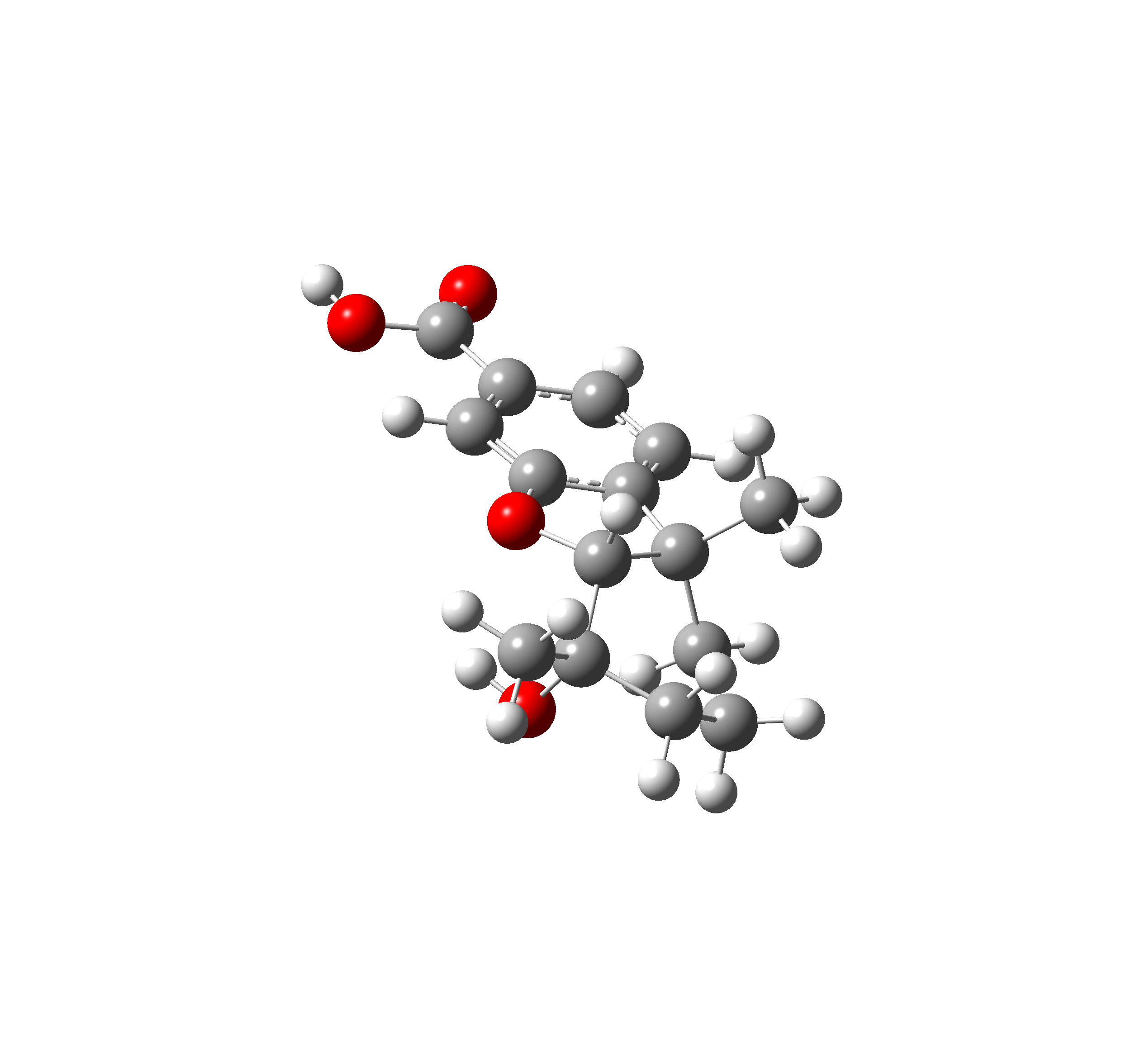 | 8.78 |

**Figure S18.** ECD conformers of aspersydonol B (**2**).
